# Supplementary material for: Prevalence and incidence of physical health conditions in people with intellectual disability – a systematic review
Source: PLoS One. 2021 Aug 24;16(8):e0256294. doi: 10.1371/journal.pone.0256294 (PMC8384165; doi:10.1371/journal.pone.0256294)
Supplement: S1 File — (DOCX) [file pone.0256294.s001.docx]

**Adapted Newcastle-Ottawa Scale (NOS)**

**Table S1. Adapted Newcastle-Ottawa Scale (NOS) for studies without comparator groups**

Note: one asterisk represents one point for the appraisal item and two asterisks represents two points. For example, for representativeness of the ID cohort, if a study meets the criteria with two asterisks, then this study will be assigned two points.

| **Original version of NOS for cohort studies (see** [**http://www.ohri.ca/programs/clinical_epidemiology/oxford.asp**](http://www.ohri.ca/programs/clinical_epidemiology/oxford.asp)**)** | **Adapted version of NOS for longitudinal cohort studies (0-9 points)** | **Adapted version of NOS for cross-sectional studies (0-8 points)** |
| --- | --- | --- |
| **SELECTION**  **(a maximum of one point for each numbered item within this category):** | **(0-4 points)** | **(0-5 points)** |
| **1. Representativeness of the exposed cohort:**  a) Truly representative of the average _______________ (describe) in the community *  b) Somewhat representative of the average ______________ in the community *  c) Selected group of users e.g. nurses, volunteers  d) No description of the derivation of the cohort | **1. Representativeness of the ID cohort:**   1. All individuals in, or randomly sampled from, a region/country (e.g. national/regional survey or national registry) ** 2. All individuals in, or randomly sampled from, disability services in the region or country (e.g. disability service users or providers) * 3. Samples from other convenient sample frames (e.g. volunteers/support group) representative of the local ID population 4. Others | **1. Representativeness of the ID cohort:**   1. All individuals in, or randomly sampled from, a region/country (e.g. national/regional survey or national registry) ** 2. All individuals in, or randomly sampled from, disability services in the region or country (e.g. disability service users or providers) * 3. Samples from other convenient sample frames (e.g. volunteers/support group) representative of the local ID population 4. Others |
| **2. Selection of the non-exposed cohort:**  a) Drawn from the same community as the exposed cohort *  b) Drawn from a different source  c) No description of the derivation of the non-exposed cohort | Not applicable | Not applicable |
|  |  | **2. Response rates:**   1. Response rate ≥ 70% * 2. Response rate <70% |
|  |  | **3. The description of non-respondents:**   1. The characteristics of non-respondents is provided and compared with the respondents, and non-respondents are unlikely to introduce bias * 2. The characteristics of non-respondents is provided and compared with the respondents, and non-respondents are likely to introduce bias 3. The characteristics of non-respondents is not described |
| **3. Ascertainment of exposure:**  a) Secure record (e.g. surgical records) *  b) Structured interview *  c) Written self-report  d) No description | **2. Ascertainment of ID status:**   1. Clinical assessment based on professional standards/extracted from health registries/medical file * 2. Self-report or informant-report 3. No description | **4. Ascertainment of ID status:**   1. Clinical assessment based on professional standards/extracted from health registries/medical * 2. Self-report or informant-report 3. No description |
| **4. Demonstration that outcome of interest was not present at start of study:**  a) Yes *  b) No | **3. Demonstration that outcome of interest was not present at start of study:**  a) Yes *  b) No |  |
| **COMPARABILITY**  **(a maximum of two points for each numbered item within this category)** | **(0-2 points)** | **(0-2 points)** |
| **1. Comparability of cohorts on the basis of the design or analysis:**  a) Study controls for _____________ (select the most important factor) *  b) Study controls for any additional factor (these criteria could be modified to indicate specific control for a second important factor) * | **1. Comparability:**   1. Standardised estimates or stratified estimates based on sex or age provided * 2. Standardised estimates or stratified estimates based on the severity of ID provided * (This point will be assigned only if a) is fulfilled) | **1. Comparability:**   1. Standardised estimates or stratified estimates based on sex or age provided * 2. Standardised estimates or stratified estimates based on the severity of ID provided * (This point will be assigned only if a) is fulfilled) |
| **OUTCOME**  **(a maximum of one point for each numbered item within this category)** | **(0-3 points)** | **(0-1 point)** |
| **1. Assessment of outcome**  a) Independent blind assessment * (e.g. x-rays, medical records, etc.)  b) Record linkage * (e.g. identified through ICD codes on database records)  c) Self-report (i.e. no reference to original medical records or x-rays to confirm the outcome)  d) No description | **1. Ascertainment of outcome:**   1. Clinical assessment based on professional standards (including structured interview in some cases e.g. sleeping disorders)/extracted from health registries/medical file * 2. Self-report or informant-report 3. No description | **1. Ascertainment of outcome:**   1. Clinical assessment based on professional standards (including structured interview in some cases e.g. sleeping disorders)/extracted from health registries/medical file * 2. Self-report or informant-report 3. No description |
| **2. Was follow-up long enough for outcomes to occur:**  a) Yes (select an adequate follow up period for outcome of interest) *  b) No | **2. Was follow-up long enough for outcomes to occur:**  a) Yes (follow-up duration justified or ≥1 year) *  b) No |  |
| **3. Adequacy of follow up of cohorts:**  a) Complete follow up - all subjects accounted for *  b) Subjects lost to follow up unlikely to introduce bias - small number lost - > ____ % (select an  adequate %) follow up, or description provided of those lost) *  c) Follow up rate < ____% (select an adequate %) and no description of those lost  d) No statement | **3. Adequacy of follow up of cohorts:**   1. Complete follow-up – all subjects accounted for * 2. Subjects lost to follow up unlikely to introduce bias (description provided of those lost) * 3. No description of those lost 4. No statement |  |

**Table S2. Adapted Newcastle-Ottawa Scale (NOS) for studies with comparator cohorts (prevalence or incidence)**

Note: one asterisk represents one point for the appraisal item and two asterisks represents two points. For example, for representativeness of the ID cohort, if a study meets the criteria with two asterisks, then this study will be assigned two points.

| **Original version of NOS for cohort studies (see** [**http://www.ohri.ca/programs/clinical_epidemiology/oxford.asp**](http://www.ohri.ca/programs/clinical_epidemiology/oxford.asp)**)** | **Adapted version of NOS for cohort studies**  **(0-11 points)** | **Adapted version of NOS for cross-sectional studies**  **(0-10 points)** |
| --- | --- | --- |
| **SELECTION**  **(a maximum of one point for each numbered item within this category):** | **(0-5 points)** | **(0-6 points)** |
| **1. Representativeness of the exposed cohort:**  a) Truly representative of the average _______________ (describe) in the community *  b) Somewhat representative of the average ______________ in the community *  c) Selected group of users e.g. nurses, volunteers  d) No description of the derivation of the cohort | **1. Representativeness of the ID cohort:**   1. All individuals in, or randomly sampled from, a region/country (e.g. national/regional survey or national registry) ** 2. All individuals in, or randomly sampled from, disability services in the region or country (e.g. disability service users or providers) * 3. Samples from other convenient sample frames (e.g. volunteers/support group) representative of the local ID population 4. Others | **1. Representativeness of the ID cohort:**   1. All individuals in, or randomly sampled from, a region/country (e.g. national/regional survey or national registry) ** 2. All individuals in, or randomly sampled from, disability services in the region or country (e.g. disability service users or providers) * 3. Samples from other convenient sample frames (e.g. volunteers/support group) representative of the local ID population 4. Others |
| **2. Selection of the non-exposed cohort:**  a) Drawn from the same community as the exposed cohort *  b) Drawn from a different source  c) No description of the derivation of the non-exposed cohort | **2. Selection of the non-ID cohort:**   1. Non-ID population or the general population drawn from the same source population as the ID cohort (e.g. in the same community) * 2. Drawn from a different source (e.g. published data) 3. no description of the derivation of the non-exposed cohort | **2. Selection of the non-ID cohort:**   1. Non-ID population or the general population drawn from the same source population as the ID cohort (e.g. in the same community) * 2. Drawn from a different source (e.g. published data) 3. no description of the derivation of the non-exposed cohort |
|  |  | **3. Response rates:**   1. Response rate ≥ 70% * 2. Response rate <70% |
|  |  | **4. The description of non-respondents:**   1. The characteristics of non-respondents is provided and compared with the respondents, and non-respondents are unlikely to introduce bias * 2. The characteristics of non-respondents is provided and compared with the respondents, and non-respondents are likely to introduce bias 3. The characteristics of non-respondents is not described |
| **3. Ascertainment of exposure**  a) Secure record (e.g. surgical records) *  b) Structured interview *  c) Written self-report  d) No description | **3. Ascertainment of ID status:**   1. Clinical assessment based on professional standards/extracted from health registries/medical file * 2. Self-report or informant-report 3. No description | **5. Ascertainment of ID status:**   1. Clinical assessment based on professional standards/extracted from health registries/medical * 2. Self-report or informant-report 3. No description |
| **Demonstration that outcome of interest was not present at start of study:**  a) Yes *  b) No | **4. Demonstration that outcome of interest was not present at start of study:**  a) Yes *  b) No |  |
| **COMPARABILITY**  **(a maximum of two points for each numbered item within this category)** | **(0-2 points)** | **(0-2 points)** |
| **1. Comparability of cohorts on the basis of the design or analysis**  a) Study controls for _____________ (select the most important factor) *  b) Study controls for any additional factor ¯ (these criteria could be modified to indicate specific control for a second important factor) * | **1. Comparability** ^§^**^:^**   1. Study controls for sex or age during study design or statistical analyses * 2. Study controls for additional factor – socioeconomic status * (This point will be assigned only if a) is fulfilled) | **1. Comparability**^§^**:**   1. Study controls for sex or age during study design or statistical analyses * 2. Study controls for additional factor – socioeconomic status * (This point will be assigned only if a) is fulfilled) |
| **OUTCOME**  **(a maximum of one point for each numbered item within this category)** | **(0-4 points)** | **(0-2 points)** |
| **1. Assessment of outcome:**  a) Independent blind assessment * (e.g. x-rays, medical records, etc.)  b) Record linkage * (e.g. identified through ICD codes on database records)  c) Self-report (i.e. no reference to original medical records or x-rays to confirm the outcome)  d) No description | **1. Ascertainment of outcome:**   1. Clinical assessment based on professional standards (including structured interview in some cases e.g. sleeping disorders)/extracted from health registries/medical file * 2. Self-report or informant-report 3. No description | **1. Ascertainment of outcome:**   1. Clinical assessment based on professional standards (including structured interview in some cases e.g. sleeping disorders)/extracted from health registries/medical file * 2. Self-report or informant-report 3. No description |
| **2. Was follow-up long enough for outcomes to occur**  a) Yes (select an adequate follow up period for outcome of interest) *  b) No | **2. Was follow-up long enough for outcomes to occur**  a) Yes (follow-up duration justified or ≥1 year) *  b) No |  |
| **3. Adequacy of follow up of cohorts**  a) Complete follow up - all subjects accounted for *  b) Subjects lost to follow up unlikely to introduce bias - small number lost - > ____ % (select an  adequate %) follow up, or description provided of those lost) *  c) Follow up rate < ____% (select an adequate %) and no description of those lost  d) No statement | **3. Adequacy of follow up of cohorts**   1. Complete follow-up – all subjects accounted for * 2. Subjects lost to follow up unlikely to introduce bias (description provided of those lost) * 3. No description of those lost 4. No statements |  |
|  | **4. Statistical model:**   1. Appropriate statistical model is chosen to estimate the effect of ID* 2. The statistical model is inappropriate 3. No statement | **2. Statistical model:**   1. Appropriate statistical model is chosen to estimate the effect of ID * 2. The statistical model is inappropriate 3. No statement |

§ When assessing comparability, socioeconomic status was selected as the second important factor. This is based on evidence showing that some of the disparity in reported health status between people with and without ID can be explained by differences in socioeconomic status.^1^

Reference:

1. Emerson E, Hatton C. Contribution of socioeconomic position to health inequalities of British children and adolescents with intellectual disabilities. *Am J Ment Retard*. 2007 Mar; 112(2):140-50. doi: 10.1352/0895-8017(2007)112[140:COSPTH]2.0.CO;2.

**Table S3. Classification applied to the adapted Newcastle-Ottawa Scale (NOS) study quality scores**

|  | Low | Moderate | High | Maximum |
| --- | --- | --- | --- | --- |
| Prevalence/Incidence |  |  |  |  |
| Cross-sectional | 0-4 | 5-6 | 7-8 | 8 |
| Longitudinal | 0-5 | 6-7 | 8-9 | 9 |
| Relative risk |  |  |  |  |
| Cross-sectional | 0-6 | 7-8 | 9-10 | 10 |
| Longitudinal | 0-7 | 8-9 | 10-11 | 11 |

* In each study, the estimates of prevalence and/or incidence were assigned a quality score ranging from 0 to 8 and 0 to 9, respectively. This was estimate-based because one study could have multiple estimates generated by different methods. Studies, regardless of their study design, that also reported relative risks (e.g., prevalence ratio) or odds ratios were able to earn two additional points. This involved an appraisal of the control selection and the choice of statistical models, resulting in a total score of 0 to 10 for cross-sectional and 0 to 11 for longitudinal cohort studies.
