# Supplementary material for: Prevalence and incidence of physical health conditions in people with intellectual disability – a systematic review
Source: PLoS One. 2021 Aug 24;16(8):e0256294. doi: 10.1371/journal.pone.0256294 (PMC8384165; doi:10.1371/journal.pone.0256294)
Supplement: S2 File — (DOCX) [file pone.0256294.s002.docx]

**Characteristics of included studies**

**Table S4. Characteristics of included studies that examined one or more physical disorders in people with ID of any aetiology**

| Author (year) | Location | ID population source | ID sample size | ID levels | ID age range (years) | ID ascertainment | ID classification | Health outcome(s) | Quality* |
| --- | --- | --- | --- | --- | --- | --- | --- | --- | --- |
| Ashman et al. (1996)[1] | Australia | Multiple sources incl. disability service and word-of-mouth | 446 | All | ≥55 | Self or informant-reported | AAMR | Multiple | 4 |
| Bhaumik et al. (2008)[2] | UK | Regional registry | 1119 | NR | ≥25 | Medical record/registry | ICD-10 | Multiple | 4 |
| Boyle et al. (2010)[3] | UK | GP, disability, and health services | 1023 | All | ≥16 | Medical record/registry | NR | Multiple | 6 |
| Burke et al. (2017)[4] | Ireland | National registry | 753 | All | ≥40 | NR | NR | Multiple | 4 |
| Carey et al. (2016)[5] | UK | Primary care database | 14751 | NR | 18-84 | Medical record/registry | QOF | Multiple | 7 |
| Cooper et al. (2018)[6] | UK | Primary care database | 721 | All | 18-92 | Medical record/registry | NR | Multiple | 6 |
| Cooper et al. (2015)[7] | UK | Primary care database | 8014 | NR | ≥18 | Medical record/registry | QOF | Multiple | 7 |
| Finlayson et al. (2010)[8] | UK | Primary care database | 511 | All | 16-79 | Medical record/registry/ clinical examination | ICD-10 for some | Multiple | 7 |
| Folch-Mas et al. (2017)[9] | Spain | National survey | 431 | Mild | NR | Self or informant-reported | NR | Multiple | 3 |
| Haveman et al. (2011)[10] | Europe | Disability service providers | 1253 | All | 19-90 | NR | NR | Multiple | 2 |
| Kinnear et al. (2018)[11] | UK | Primary care database and disability services | 1023 | All | 16-83 | Medical record/registry | ICD-10 | Multiple | 4 |
| McCarron et al. (2017)[12] | Ireland | National registry | 478 | NR | ≥50 | NR | NR | Multiple | 4 |
| McCarron et al. (2013)[13] | Ireland | National registry | 753 | All | ≥40 | NR | NR | Multiple | 4 |
| Melville et al. (2008)[14] | UK | Primary care database and disability services | 945 | All | ≥16 | Medical record/registry | ICD-10 DCR | Multiple | 6 |
| Morin et al. (2012)[15] | Canada | Multiple sources incl. health services | 789 | All | ≥15 | NR | AAIDD | Multiple | 3 |
| Wee et al. (2014)[16] | Singapore | Disability service providers | 227 | NR | ≥40 | NR | NR | Multiple | 4 |
| Beange et al. (1995)[17] | Australia | Local agencies | 202 | All | 20-50 | Clinical examination | AAMR (1983) | Multiple | 6 |
| Atladottir et al. (2015)[18] | Denmark | National birth registry | 6592 | NR | 0-16 | Medical record/registry | ICD-10  (F70-F79) | Neonatal and congenital disorders | 7 |
| Christianson et al. (2002)[19] | South Africa | Regional survey | 43 | Severe to profound | 2-9 | Clinical examination | TQQ, GSMD and GIQ | Congenital and neurological disorders | 7 |
| Wellesley et al. (1992)[20] | Australia | Disability service and health care providers | 1602 | All | 6-16 | Clinical examinations | AAMD | Neurological and sensory disorders | 8 |
| Arvio et al. (2003)[21] | Finland | Disability services | 461 | Severe to profound | 1-72 | Medical record/registry | NR | Neurological and sensory disorders | 5 |
| Hand et al. (1996)[22] | New Zealand | Disability service providers | 1063 | NR | >50 | NR | NR | Neurological disorders and visual impairment | 4 |
| Lin et al. (2006)[23] | Taiwan | National registry | 1071 | All | 25.5 (13.6) (mean, SD) | Medical record/registry | The Protection Law for the Disabled | Neurological disorders and visual impairment | 2 |
| Richdale et al. (2000)[24] | Australia | Disability service providers | 52 | All | 1.8-19 | NR | NR | Epilepsy and asthma | 2 |
| Gale et al. (2009)[25] | UK | Primary care database | 1097 | NR | ≥16 | Medical record/registry | NR | Obesity and asthma | 7 |
| Segal et al. (2016)[26] | US | National survey | 672 | NR | 10-17 | Self or informant-reported | NR | Obesity and asthma | 3 |
| Slevin et al. (2014)[27] | Northern Ireland | Schools (5 special and 13 mainstream) | 228 | NR | ≤19 | Non-health administrative registry | NR | Obesity and epilepsy | 4 |
| Stancliffe et al. (2012)[28] | US | Disability service providers | 12381 | All | 18-100 | Self or informant-reported | NR | Overweight and visual impairment | 3 |
| Lunsky et al. (2017)[29] | Canada | Disability support and health record | 64008 | NR | 18-64 | Medical record/registry | NR | HIV | 7 |
| Patja et al. (2001)[30] | Finland | National survey | 2173 | All | 7-69 | Clinical examination | ICD | Cancer | 7 |
| Sullivan et al. (2004)[31] | Australia | Disability service providers | 9409 | NR | 30.8 (16.3) | Medical record/registry | Heber classifications | Cancer | 6 |
| Hove et al. (2004)[32] | Norway | Disability service providers | 282 | All | ≥18 | Medical registry or clinical checklist | ICD-10 for some | Obesity | 6 |
| Simila et al. (1991)[33] | Finland | Birth cohort | 112 | All | 20 | NR | NR | Obesity | 6 |
| Mikulovic et al. (2011)[34] | France | Schools and institutes | 410 | NR | 11-20 | Medical record/registry | NR | Obesity | 5 |
| Carter et al. (2009)[35] | UK | Regional registry | 58 | All | <18 | NR | NR | Obesity | 4 |
| Lin et al. (2005)[36] | Taiwan | National registry | 279 | All | 4-18 | Medical record/registry | The Protection Law for the Disabled | Obesity | 3 |
| Moore et al. (2004)[37] | Australia | Disability service providers | 93 | Mild to severe | 18-63 | NR | NR | Obesity | 3 |
| Stancliffe et al. (2011)[38] | US | Disability service providers | 8911 | All | 20-93 | Self or informant-reported | NR | Obesity | 3 |
| Yen et al. (2005)[39] | Taiwan | National registry | 516 | All | 18-80 | NR | The Protection Law for the Handicapped of 1997 in Taiwan | Obesity | 3 |
| Van de Louw et al. (2009)[40] | Netherlands | Disability service providers | 213 | All | 18-91 | Medical record/registry | NR | Hyper-tension | 4 |
| Tyrer et al. (2020)[41] | UK | Regional registry | 1091 | NR | 18-80 | NR | NR | Diabetes | 4 |
| Benassi et al. (1990)[42] | Italy | Mainstream schools | 90 | Severe to profound | 6-13 | Medical record/registry | IQ<50 | Neurological disorders | 7 |
| Gustavson et al. (1977)[43] | Sweden | Disability service registry (regional) | 122 | Severe to profound | 5-16 | Clinical examination | IQ<50 | Neurological disorders | 7 |
| McQueen et al. (1987)[44] | Canada | Regional census | 221 | Moderate to profound | 7-10 | NR | NR | Neurological disorders | 6 |
| Janicki et al. (1984)[45] | US | Disability service providers | unclear | Mild to moderate | 73-99 | NR | NR | Neurological disorders | 5 |
| Yen et al. (2009)[46] | Taiwan | National registry | 1419 | All | 12-17 | Medical record/registry | The ‘PMDCPA (2004)’ of Taiwan | Neurological disorders | 4 |
| Matthews et al. (2008)[47] | UK | Primary care database | 317 | NR | 17-86 | Medical record/registry | NR | Epilepsy | 6 |
| Morgan et al. (2003)[48] | UK | Health and disability services | 1595 | NR | ≥15 | Non-health/health administrative registry | ICD-9 | Epilepsy | 6 |
| Forsgren et al., 1990[49] | Sweden | Multiple sources incl. regional disability registry | 1479 | All | 0-79 | Clinical examination | ICD-9 and DSM-III-R | Epilepsy | 5 |
| McGrother et al. (2006)[50] | UK | Regional registry | 2393 | NR | ≥20 | NR | ICD | Epilepsy | 5 |
| Shepherd et al. (1989)[51] | UK | Schools for ID or special units in mainstream schools | 643 | Mild to severe | 5-16 | Non-health administrative registry/medical record/registry | NR | Epilepsy | 5 |
| Lin et al. (2003)[52] | Taiwan | National registry | 1071 | NR | 25.5 (13.6) (mean, SD) | Medical record/registry | ICD-8 | Epilepsy | 4 |
| Lund (1985)[53] | Denmark | National services registry | 302 | NR | ≥20 | NR | ICD-10 | Epilepsy | 4 |
| McCarron et al. (2014)[54] | Ireland | National registry | 753 | All | ≥40 | NR | NR | Epilepsy | 4 |
| McGrother et al. (1996)[55] | Leicestershire, UK | Regional registry | 2117 | NR | ≥20 | NR | ICD | Epilepsy | 4 |
| Warburg (2001)[56] | Denmark | Pension recipients | 837 | Moderate to profound | 20-99 | NR | NR | Visual impairment and other eye disorders | 4 |
| van Splunder et al. (2003)[57] | Netherlands | Disability service providers | 505 | NR | 20.1-88.7 | Medical record/registry | AAMR | Eye disorders | 3 |
| Meuwese- Jongejeugd et al. (2006)[58] | Netherlands | Disability service providers | 1215 | All | 20.19-88.73 | Medical record/registry | NR | Hearing loss | 5 |
| Pradhan et al. (2009)[59] | Australia | Disability service providers | unclear | NR | 18-44 | NR | NR | Dental caries | 3 |
| Burke et al. (2019)[60] | Ireland | National registry | 575 | All | ≥43 | NR | NR | Osteoporosis | 5 |
| AlMutairi et al. (2020)[61] | Ireland | National registry | 632 | All | ≥44 | NR | NR | Constipation | 3 |
| Bishop et al. (2020)[62] | US | National registry | 21167 | NR | ≥21 | Medical record/registry | ICD-9 or ICD-10 | Multiple | 7 |
| Cuypers et al. (2021)[63] | Netherlands | Disability service providers | 21203 | NR | All ages | Medical record/registry | NR | Diabetes | 6 |
| Garcia et al. (2020)[64] | Spain | Disability service providers | 1040 | All | 44-88 | Medical record/registry | NR | Multiple | 2 |
| McMahon et al. (2021)[65] | Jersey | Disability service providers | 217 | All | 18-85 | NR | NR | Cancer | 4 |
| Monaghan et al. (2021)[66] | Ireland | National registry | 609 | All | 48-95 | NR | NR | Epilepsy | 4 |
| O'Brien (2020)[67] | Ireland | National registry | 551 | All | 44-92 | NR | NR | Multiple | 4 |
| Tyrer et al. (2020)[41] | UK | Regional registry | 1091 | NR | ≥18 | NR | NR | Multiple | 4 |
| Xie et al. (2020)[68] | US | National survey | 699 | NR | <18 | Self- or informant-report | NR | Asthma | 3 |

* The adapted Newcastle-Ottawa Scale (NOS) quality scores are the highest quality score for that study if multiple outcomes and scores exist.

Abbreviations used in the table:

AAIDD: American Association on Intellectual and Developmental Disabilities

AAMR: American Association on Mental Retardation

BPA6: The British Paediatric Association Classification of Diseases modified by the California Birth Defects Monitoring Program (CBDMP) in this case

CPS: The cognitive performance Scale

DCR: The Diagnostic Criteria for Research (accompanying ICD)

DSM: Diagnostic and Statistical Manual of Mental Disorders

GIQ: General intelligence quotient

GSMD: Griffiths Scale of Mental Development

ICD: International Classification of diseases

IQ: Intelligence quotient

NR: Not reported

PMDCPA: The Physically and Mentally Disabled Citizens Protection Act

QOF: Quality Outcomes Framework

TQQ: Ten Question Questionnaire (the internationally validated)

**Table S5. Characteristics of included studies that examined one or more physical disorders in people with genetic syndromes**

| Author (year) | Location | ID population source | ID sample size | ID levels | ID age range (years) | ID ascertainment | ID classification | Health outcome(s) | Quality* |
| --- | --- | --- | --- | --- | --- | --- | --- | --- | --- |
| Down syndrome | |  |  |  |  |  |  |  |  |
| Baccichetti et al. (1990)[69] | Italy | Regional registry | 102 | NR | NR | Medical record/registry | NR | Multiple | 4 |
| Henderson et al. (2007)[70] | UK | Disability service providers | 64 | NR | 18-61 | Medical record/registry | NR | Multiple | 6 |
| Kapell et al. (1998)[71] | US | Regional registry | 131 | All | ≥45 | NR | NR | Multiple | 6 |
| Leonard et al. (1999)[72] | Australia | Disability services and birth defect registry | 211 | NR | 5-17 | Medical record/registry | NR | Multiple | 7 |
| Määttä et al. (2011)[73] | Finland | Disability service registry (regional) | 84 | All | 0.6-59.2 | Medical record/registry | NR | Multiple | 5 |
| McGrother et al. (1990)[74] | UK | Regional registry | 107 | NR | 1-10 | Medical record/registry | NR | Multiple | 7 |
| Pikora et al. (2014)[75] | Australia | Regional Down syndrome database | 197 | NR | 16.3-31.9 | Medical record/registry | NR | Multiple | 4 |
| Prasher et al. (2014)[76] | UK | Primary care database | 130 | NR | 18-43 | Medical record/registry | NR | Multiple | 4 |
| Roizen et al. (2014)[77] | US | Surveillance program | 440 | NR | 3-14 | Medical record/registry | NR | Multiple | 4 |
| Thomas et al. (2011)[78] | Australia | Disability service providers | 208 | NR | 5.02-17.98 | Clinical examination | NR | Multiple | 4 |
| Startin et al. (2020)[79] | UK | Multiple sources incl. disability and healthcare services | 115 | NR | 0-73 | Clinical examination | NR | Multiple | 4 |
| Arnell et al. (2012)[80] | Sweden | Hospital database (birth cohorts) | 206 | NR | Infants | Medical record/registry | NR | Neonatal and congenital disorders | 7 |
| Weijerman et al. (2010)[81] | Netherlands | National registry | 482 | NR | Infants | Medical record/registry | NR | Neonatal and congenital disorders | 5 |
| Fabia et al. (1970)[82] | US | Multiple sources incl. paediatric hospital | 2421 | NR | Young children | Medical record/registry | NR | Congenital malform-ations and leukemia | 7 |
| Hamilton et al. (2016)[83] | UK | Hearing surveillance clinic | 239 | NR | 0-20 | Medical record/registry | NR | Congenital and respiratory disorders | 7 |
| Jaruratanasirikul et al. (2017)[84] | Thailand | Regional registry | 149 | NR | Infants | Medical record/registry | NR | Congenital heart defects and other disorders | 7 |
| Garg et al. (2018)[85] | US | Healthcare organisations (case identification validated) | 11936 | NR | ≥18 | Medical record/registry | SNOMED | Hidradenitis suppurativa and obesity | 6 |
| Haugen et al. (2001)[86] | Norway | Disability service providers | 60 | NR | Children | Medical record/registry | NR | Neurological and eye disorders | 4 |
| Boker et al. (2001)[87] | Israel | National registry | 1864 | NR | <17 | Medical record/registry | NR | Cancer | 8 |
| Hasle et al. (2016)[88] | Denmark | National registry | 3530 | NR | NR | Medical record/registry | NR | Cancer | 8 |
| Bjørge et al. (2008)[89] | Sweden and Norway | National birth registry | S:3201; N:2108 | NR | <40 | Medical record/registry | ICD 8-10 | Cancer | 7 |
| Patja et al. (2006)[90] | Finland | Disability service providers | 3581 | NR | 0-79 | Medical record/registry | ICD-8 | Cancer | 6 |
| Sullivan et al. (2007)[91] | Australia | Disability service providers | 1298 | NR | Baseline: 20.2 (13.9) | Medical record/registry | NR | Cancer | 6 |
| Murphy et al. (2008)[92] | Dublin, Wicklow and Kildare; Ireland | Multiple sources incl. EUROCAT registry | 390 | NR | ≤18 | NR | NR | Disorders of thyroid gland | 4 |
| Melville et al. (2005)[93] | UK | Regional registry | 247 | NR | 20-69 | Medical record/registry | NR | Obesity | 6 |
| Prasher et al. (1995)[94] | UK | Multiple sources incl. health services | 201 | All | 16-76 | Medical record/registry | ICD-10 for the severity of ID | Obesity | 5 |
| Carfi et al. (2019)[95] | USA, Canada and Italy ^**^ | Disability service users | 175 | NR | 18-62 | Clinical examination | CPS | Obesity | 3 |
| Johannsen et al. (1996)[96] | Denmark | National registry | 72 | NR | 14-60 | Medical record/registry | NR | Epilepsy | 6 |
| Barr et al. (2011)[97] | UK | Hearing surveillance clinic | 87 | NR | <6 | Medical record/registry | NR | Disorders of ears | 6 |
| Yaneza et al. (2016)[98] | UK | Multiple sources incl. health services | 102 | NR | 5-12 | NR | NR | Disorders of ears | 6 |
| Austeng et al. (2013)[99] | Norway | National registry | 52 | NR | 8 | Medical record/registry | NR | Disorders of ears | 5 |
| Austeng et al. (2013)[100] | Norway | National registry | 49 | NR | 8 | Medical record/registry | NR | Hearing impairment | 5 |
| Park et al. (2012)[101] | US | Regional birth defects registry | 332 | NR | Infants | Medical record/registry | NR | Hearing impairment | 6 |
| Jansson et al. (1995)[102] | Sweden | Disability service providers | 56 | NR | <18 | NR | NR | Celiac disease | 5 |
| Brodwall et al. (2018)[103] | Norway | National registry | 1251 | NR | 0-5 | Medical record/registry | ICD 8-10 | Congenital malform-ations | 7 |
| Torfs et al. (1998)[104] | US | Birth defect registry | 2894 | NR | Infants (<1) | Medical record/registry | BPA6 | Congenital malform-ations | 7 |
| Kupferman et al. (2009)[105] | US | Regional registry | 3832 | NR | Infants | Medical record/registry | NR | Congenital malform-ations | 6 |
| Bergström et al. (2016)[106] | Sweden | National healthcare registries | 2588 | NR | Infants | Medical record/registry | ICD 8-10 | Congenital heart defects | 7 |
| Freeman et al. (2008)[107] | US | National Down syndrome project | 1469 | NR | Infants | Medical record/registry | NR | Congenital heart defects | 7 |
| Freeman et al. (1998)[108] | US | Birth cohorts | 227 | NR | Infants | Medical record/registry | NR | Congenital heart defects | 7 |
| Kim et al. (2014)[109] | Korea | Medical insurance claim database | 394 | NR | ≤1 | Medical record/registry | ICD-10 | Congenital heart defects | 7 |
| Santoro et al. (2018)[110] | Italy | Regional registry | 230 | NR | Infants | Medical record/registry | NR | Congenital heart defects | 7 |
| Scott et al. (2014)[111] | Jamaica | Birth cohort | 53 | NR | Infants | Clinical examination | Smith’s Recognizable Patterns of Human Malformation | Congenital heart defects | 7 |
| So et al. (2007)[112] | US | Birth cohort | 213 | NR | 0-3 | Medical record/registry | ICD-9 | Congenital heart defects | 7 |
| Irving et al. (2012)[113] | UK | Regional survey | 821 | NR | Infants | NR | NR | Congenital heart defects | 6 |
| Freeman et al. (2009)[114] | US | Regional Down syndrome project databases | 1892 | NR | Infants | Medical record/registry | NR | Congenital malform-ations of the digestive system | 7 |
| Tedeschi et al. (2015)[115] | US | Birth cohort and other referral cases | 109 | NR | Infants | Medical record/registry | NR | Congenital hearing loss | 5 |
| Cho et al. (2020)[116] | South Korea | National survey | 2077 | NR | All ages | Medical record/registry | ICD-10 or specific RDR designation for DS | Congenital heart defects | 6 |
| Kristianslund et al. (2021)[117] | Norway | National registry | 4342 | NR | NR | Medical record/registry | ICD-10 | Keratoconus | 6 |
| O'Brien (2020)[67] | Ireland | National registry | 100 | NR | NR | Self- or informant-report | NR | Hyperten-sion | 4 |
| Ostermaier et al. (2020)[118] | US | Birth cohort | 45 | NR | All ages | Medical record/registry | ICD-9 and HICDA | Celiac disease | 6 |
| Tyrer et al. (2020)[41] | UK | Regional registry | 144 | NR | ≥18 | NR | NR | Diabetes | 4 |
|  |  |  |  |  |  |  |  |  |  |
| Fragile X syndrome | |  |  |  |  |  |  |  |  |
| Schultz-Pedersen et al. (2001)[119] | Denmark | Regional registry | 223 | NR | NR | Medical record/registry | Cytogenetic test | Cancer | 8 |
| Sund et al. (2009)[120] | Finland | Disability service registry | 302 | All | NR | NR | NR | Cancer | 5 |
| Sabaratnam et al. (2001)[121] | UK | Disability service providers | 24 | NR | 13-63 | Medical record/registry | NR | Epilepsy | 4 |
|  |  |  |  |  |  |  |  |  |  |
| Prader-Willi syndrome | | |  |  |  |  |  |  |  |
| Butler et al. (2002)[122] | UK | Disability services & associations | 66 | NR | 0-46 | Medical record/registry | NR | Multiple | 4 |
| Diene et al. (2010)[123] | France | National pediatric database | 142 | NR | ≤18 | Medical record/registry | Genetic tests | Multiple | 7 |
| Patja et al. (2008)[124] | Finland | Disability service providers | 56 | NR | 0-79 | Medical record/registry | ICD-8 | Cancer | 6 |
|  |  |  |  |  |  |  |  |  |  |
| Rett syndrome | |  |  |  |  |  |  |  |  |
| Downs et al. (2008)[125] | Australia | National Rett database | 234 | NR | 2-29 | Medical record/registry | NR | Epilepsy | 3 |
| Freilinger et al. (2014)[126] | Australia | National Rett database | 270 | NR | 3-47 | NR | NR | Cholelith-iasis | 2 |
| Downs et al. (2016)[127] | Australia | National Rett database | 394 | NR | NR | Medical record/registry | NR | Scoliosis | 3 |
|  |  |  |  |  |  |  |  |  |  |
| Bardet-Biedl syndrome | | |  |  |  |  |  |  |  |
| Hjortshøj et al. (2007)[128] | Denmark | National registry | 116 | NR | NR | Medical record/registry | NR | Cancer | 7 |
|  |  |  |  |  |  |  |  |  |  |
| Angelman syndrome | |  |  |  |  |  |  |  |  |
| Thomson et al. (2006)[129] | Australia | Disability service providers | 34 | All | 6.5-39.0 | Medical record/registry | NR | Scoliosis | 5 |
|  |  |  |  |  |  |  |  |  |  |
| Velocardiofacial syndrome and William syndrome | | | |  |  |  |  |  |  |
| Zarchi et al. (2011)[130] | Israel | Medical center | 62 | NR | 6.2-46.9 | Medical record/registry | NR | Hearing loss | 4 |

* The adapted Newcastle-Ottawa Scale (NOS) quality scores are the highest quality score for that study if multiple outcomes and scores exist.

** Only the data from USA met the inclusion criteria and were included in the review.

Abbreviations used in the table:

BPA6: The British Paediatric Association Classification of Diseases modified by the California Birth Defects Monitoring Program (CBDMP) in this case

CPS: The cognitive performance Scale

DCR: The Diagnostic Criteria for Research (accompanying ICD)

HICDA: Hospital Adaptation of the International Classification of Diseases

ICD: International Classification of diseases

NR: Not reported

RDR: Rare Diseases Registry

SNOMED: Systemized Nomenclature of Medicine–Clinical Terms

**Table S6. Characteristics of included studies that examined one or more physical disorders in people with ID of any aetiology and a non-ID population**

| Author (year) | Location | ID sample size | Non-ID sample size | Non-ID population source | Factors controlled for | Quality* |
| --- | --- | --- | --- | --- | --- | --- |
| Atladottir et al. (2015)[18] | Denmark | 6592 | 1106050 | All children born in Denmark during the same time period | None | 8 |
| Carey et al. (2016)[5] | UK | 14751 | 86221 | A subset of people without ID extracted from 408 English GPs during the same time period | Age, sex, and practice | 10 |
| Cooper et al. (2015)[7] | UK | 8014 | 1416364 | All people without ID extracted from 314 Scottish GPs during the same time period | Age, sex, and deprivation score | 10 |
| Lunsky et al. (2017)[29] | Canada | 64008 | 2804662 | Random sample without ID identified using the Registered Persons Database during the same time period | Age and sex | 9 |
| McCarron et al. (2017)[12] | Ireland | 478 | 478 | A stratified sample of individuals aged 50 in Ireland (2010 TILDA) | Age, sex, and geographic location | 6 |
| Richdale et al. (2000)[24] | Australia | 52 | 25 | A convenience sample of children without ID | None | 2 |
| Segal et al. (2016)[26] | US | 672 | 43146 | Participants without ID from the same national survey | None | 6 |
| Slevin et al. (2014)[27] | Northern Ireland | 228 | 232 | Random sample without ID from mainstream schools recruited during the same time period | School and class level | 5 |
| Bhaumik et al. (2008)[2] | UK | 1119 | N/A | 1998 England Health Survey data | Standardised on age; stratified by sex | 5 |
| Cooper et al. (2018)[6] | UK | 721 | 764672 | The general population sourced from Scottish GPs in 2006/2007 | None | 7 |
| Finlayson et al. (2010)[8] | UK | 511 | 8148 | 2003 Scottish Health Survey data | Age | 8 |
| Melville et al. (2008)[14] | UK | 945 | N/A | 2003 Scottish Health Survey data | Stratified by age and sex | 6 |
| Moore et al. (2004)[37] | Australia | 93 | N/A | Two published statistics in Australia: one for Victoria in 1996 and one for Australia in 1995 | None | 3 |
| Morgan et al. (2003)[48] | UK | 1595 | N/A | 1996 Cardiff and Vale population data | Age and sex | 8 |
| Morin et al. (2012)[15] | Canada | 789 | N/A | The most recent health survey data and census data for residents of Quebec (2005) | Age | 3 |
| Patja et al. (2001)[30] | Finland | 195 | N/A | Finnish national statistics | Age, sex, and calendar year | 8 |
| Sullivan et al. (2004)[31] | Australia | 9409 | N/A | WA local statistics for the general population | Age and sex | 7 |
| Beange et al. (1995)[17] | Australia | 202 | 619 | 1989 Australian community survey data | None | 6 |
| Cuypers et al. (2021)[63] | Netherlands | 21203 | 267628 | People without ID from the same source as the ID cohort | Stratified by age and sex | 7 |
| Garcia et al. (2020)[64] | Spain | 1040 | 12172 | People without ID from the 2011/12 Spanish National Health Survey | Matched on age and sex | 2 |
| McMahon et al. (2021)[65] | Jersey | 217 | 2350 | The general population randomly sampled from the same adult population in Jersey | Age and sex | 6 |
| Xie et al. (2020)[68] | US | 699 | NR | Children without specified disabilities from the same survey | Age, sex, race/ethnicity, parental educational level, family  Income, and birth weight | 6 |

*Adapted Newcastle-Ottawa Scale (NOS) quality scores are for comparisons between people with ID and people without ID or the general population.

**Table S7. Characteristics of included studies that examined one or more physical disorders in people with genetic syndromes and a non-ID population**

| Author (year) | Location | | ID sample size | Non-ID sample size | Non-ID population source | Factors controlled for | Quality* |
| --- | --- | --- | --- | --- | --- | --- | --- |
| Down syndrome |  | |  |  |  |  |  |
| Austeng et al. (2013)[99] | | Norway | 52 | 57 | Healthy children without DS considered representative for the Norwegian population | Age | 5 |
| Austeng et al. (2013)[100] | | Norway | 49 | 57 | Volunteer healthy children without DS from a larger cohort and considered representative for the Norwegian population | Age | 5 |
| Bjørge et al. (2008)[89] | | Sweden and Norway | S:3201  N:2108 | S:3035027  N:2081777 | All live infants without birth defect identified from Norway and Swedish population-based birth registries during the same time period | Age, sex, and calendar period | 10 |
| Brodwall et al. (2018)[103] | | Norway | 1251 | 942226 | All infants without chromosomal abnormalities identified from Norway population-based birth registry during the same time period | Maternal age and birth year | 9 |
| Garg et al. (2018)[85] | | US | 11936 | 16813290 | All people without DS identified from the same 27 healthcare organisation registers during the same time period | Age, sex, race, and obesity | 9 |
| Kupferman et al. (2009)[105] | | US | 3832 | 3411833 | Children without DS born in New York State in 1992 - 2004 | None (all children) | 7 |
| Torfs et al. (1998)[104] | | US | 2894 | 2490437 | All live births without DS registered in the California counties during the same time period | None (all infants) | 9 |
| Boker et al. (2001)[87] | | Israel | 1864 | N/A | National cancer statistics | Age, sex, place of birth (for some), nationality and calendar period | 9 |
| Hasle et al. (2016)[88] | | Denmark | 3530 | N/A | Danish population statistics | Age, sex, and calendar period | 9 |
| Kapell et al. (1998)[71] | | US | 131 | N/A | 1993 National Health Interview Survey | Age | 6 |
| Patja et al. (2006)[90] | | Finland | 3581 | N/A | Finnish population statistics | Age, sex, and calendar period | 7 |
| Sullivan et al. (2007)[91] | | Australia | 1298 | N/A | WA population statistics | Age | 7 |
| Weijerman et al. (2010)[81] | | Netherlands | 482 | N/A | Dutch population statistics | None | 5 |
| Cho et al. (2020)[116] | | South Korea | 2077 | 10385 | Healthy controls with unclear source | Matched on age and sex | 8 |
| Ostermaier et al. (2020)[118] | | US | 45 | N/A | Published age- and sex-adjusted incidence for Olmsted County residents during 2000 to 2010 | None | 6 |
| Velocardiofacial syndrome and William Syndrome | | | |  |  |  |  |
| Zarchi et al. (2011)[130] | | Israel | 62 | 23 | Typically developing peers of similar age range; source not reported | None | 4 |
| Fragile X syndrome | | |  |  |  |  |  |
| Sund et al. (2009)[120] | | Finland | 302 | N/A | Finnish population statistics | Age, sex, and calendar period | 6 |
| Schultz-Pedersen et al. (2001)[119] | | Denmark | 223 | N/A | Danish population statistics | Age, sex, and calendar period | 9 |
| Bardet-Biedl syndrome | | | |  |  |  |  |
| Hjortshøj et al. (2007)[128] | | Denmark | 116 | N/A | Danish population statistics | Age, sex, and calendar period | 9 |
| Prader-Willi syndrome | | | |  |  |  |  |
| Patja et al. (2008)[124] | | Finland | 56 | N/A | Finnish population statistics | Age, sex, and calendar period | 7 |

*Adapted Newcastle-Ottawa Scale (NOS) quality scores are for comparisons between people with ID and people without ID or the general population.

References:

1. Ashman AF, Suttie J. The medical and health status of older people with mental retardation in Australia. *J Appl Gerontol*. 1996;15(1):57-72. doi: <http://dx.doi.org/10.1177/073346489601500104>.

2. Bhaumik S, Watson JM, Thorp CF, Tyrer F, McGrother CW. Body mass index in adults with intellectual disability: distribution, associations and service implications: a population-based prevalence study. *J Intellect Disabil Res*. 2008;52(Pt 4):287-98. doi: <https://dx.doi.org/10.1111/j.1365-2788.2007.01018.x>.

3. Boyle A, Melville CA, Morrison J, Allan L, Smiley E, Espie CA, et al. A cohort study of the prevalence of sleep problems in adults with intellectual disabilities. *J Sleep Res*. 2010;19(1 Pt 1):42-53. doi: <https://dx.doi.org/10.1111/j.1365-2869.2009.00788.x>.

4. Burke EA, McCallion P, Carroll R, Walsh JB, McCarron M. An exploration of the bone health of older adults with an intellectual disability in Ireland. *J Intellect Disabil Res*. 2017;61(2):99-114. doi: <https://dx.doi.org/10.1111/jir.12273>.

5. Carey IM, Shah SM, Hosking FJ, DeWilde S, Harris T, Beighton C, et al. Health characteristics and consultation patterns of people with intellectual disability: a cross-sectional database study in English general practice. *Br J Gen Pract*. 2016;66(645):e264-70. doi: <https://dx.doi.org/10.3399/bjgp16X684301>.

6. Cooper SA, Hughes-McCormack L, Greenlaw N, McConnachie A, Allan L, Baltzer M, et al. Management and prevalence of long-term conditions in primary health care for adults with intellectual disabilities compared with the general population: A population-based cohort study. *J Appl Res Intellect Disabil*. 2018;31 Suppl 1:68-81. doi: <https://dx.doi.org/10.1111/jar.12386>.

7. Cooper S-A, McLean G, Guthrie B, McConnachie A, Mercer S, Sullivan F, et al. Multiple physical and mental health comorbidity in adults with intellectual disabilities: population-based cross-sectional analysis. *BMC Fam*. 2015;16(1):110. doi: 10.1186/s12875-015-0329-3.

8. Finlayson J, Morrison J, Jackson A, Mantry D, Cooper SA. Injuries, falls and accidents among adults with intellectual disabilities. Prospective cohort study. *J Intellect Disabil Res*. 2010;54(11):966-80. doi: <https://dx.doi.org/10.1111/j.1365-2788.2010.01319.x>.

9. Folch-Mas A, Cortes-Ruiz MJ, Vicens Calderon P, Martinez-Leal R. Health profiles in people with intellectual developmental disorders. *Salud Publica Mex*. 2017;59(4):400-7. doi: <https://dx.doi.org/10.21149/8199>.

10. Haveman M, Perry J, Salvador-Carulla L, Walsh PN, Kerr M, Van Schrojenstein Lantman-de Valk H, et al. Ageing and health status in adults with intellectual disabilities: Results of the European POMONA II study. *J Intellect Dev Disabil*. 2011;36(1):49-60. doi: 10.3109/13668250.2010.549464.

11. Kinnear D, Morrison J, Allan L, Henderson A, Smiley E, Cooper SA. Prevalence of physical conditions and multimorbidity in a cohort of adults with intellectual disabilities with and without Down syndrome: cross-sectional study. *BMJ Open*. 2018;8(2):e018292. doi: <https://dx.doi.org/10.1136/bmjopen-2017-018292>.

12. McCarron M, Cleary E, McCallion P. Health and Health-Care Utilization of the Older Population of Ireland: Comparing the Intellectual Disability Population and the General Population. *Res Aging*. 2017;39(6):693-718. doi: <https://dx.doi.org/10.1177/0164027516684172>.

13. McCarron M, Swinburne J, Burke E, McGlinchey E, Carroll R, McCallion P. Patterns of multimorbidity in an older population of persons with an intellectual disability: results from the intellectual disability supplement to the Irish longitudinal study on aging (IDS-TILDA). *Res Dev Disabil*. 2013;34(1):521-7. doi: <https://dx.doi.org/10.1016/j.ridd.2012.07.029>.

14. Melville C, Cooper S, Morrison J, Allan L, Smiley E, Williamson A. The prevalence and determinants of obesity in adults with intellectual disabilities. *J Appl Res Intellect Disabil*. 2008;21(5):425-37. doi: <http://dx.doi.org/10.1111/j.1468-3148.2007.00412.x>.

15. Morin D, Merineau-Cote J, Ouellette-Kuntz H, Tasse MJ, Kerr M. A comparison of the prevalence of chronic disease among people with and without intellectual disability. *Am J Intellect Dev Disabil*. 2012;117(6):455-63. doi: <https://dx.doi.org/10.1352/1944-7558-117.6.455>.

16. Wee LE, Koh GC, Auyong LS, Cheong A, Myo TT, Lin J, et al. Screening for cardiovascular disease risk factors at baseline and post intervention among adults with intellectual disabilities in an urbanised Asian society. *J Intellect Disabil Res*. 2014;58(3):255-68. doi: <https://dx.doi.org/10.1111/jir.12006>.

17. Beange H, McElduff A, Baker W. Medical disorders of adults with mental retardation: a population study. *Am J Ment Retard*. 1995;99(6):595-604.

18. Atladottir HO, Schendel DE, Parner ET, Henriksen TB. A Descriptive Study on the Neonatal Morbidity Profile of Autism Spectrum Disorders, Including a Comparison with Other Neurodevelopmental Disorders. *J Autism Dev Disord*. 2015;45(8):2429-42. doi: <https://dx.doi.org/10.1007/s10803-015-2408-7>.

19. Christianson AL, Zwane ME, Manga P, Rosen E, Venter A, Downs D, et al. Children with intellectual disability in rural South Africa: prevalence and associated disability. *J Intellect Disabil Res*. 2002;46(Pt 2):179-86.

20. Wellesley DG, Hockey KA, Montgomery PD, Stanley FJ. Prevalence of intellectual handicap in Western Australia: a community study. *Med J Aust*. 1992;156(2):94-6, 100, 2.

21. Arvio M, Sillanpaa M. Prevalence, aetiology and comorbidity of severe and profound intellectual disability in Finland. *J Intellect Disabil Res*. 2003;47(Pt 2):108-12.

22. Hand JE, Reid PM. Older adults with lifelong intellectual handicap in New Zealand: prevalence, disabilities and implications for regional health authorities. *N Z Med J*. 1996;109(1019):118-21.

23. Lin JD, Yen CF, Loh CH, Hsu SW, Huang HC, Tang CC, et al. A cross-sectional study of the characteristics and determinants of emergency care utilization among people with intellectual disabilities in Taiwan. *Res Dev Disabil*. 2006;27(6):657-67. doi: <http://dx.doi.org/10.1016/j.ridd.2005.09.001>.

24. Richdale A, Francis A, Gavidia-Payne S, Cotton S. Stress, behaviour, and sleep problems in children with an intellectual disability. *J Intellect Dev Disabil*. 2000;25(2):147-61. doi: 10.1080/13269780050033562.

25. Gale L, Naqvi H, Russ L. Asthma, smoking and BMI in adults with intellectual disabilities: a community-based survey. *J Intellect Disabil Res*. 2009;53(9):787-96. doi: <https://dx.doi.org/10.1111/j.1365-2788.2009.01192.x>.

26. Segal M, Eliasziw M, Phillips S, Bandini L, Curtin C, Kral TV, et al. Intellectual disability is associated with increased risk for obesity in a nationally representative sample of U.S. children. *Disabil Health J*. 2016;9(3):392-8. doi: <https://dx.doi.org/10.1016/j.dhjo.2015.12.003>.

27. Slevin E, Truesdale-Kennedy M, McConkey R, Livingstone B, Fleming P. Obesity and overweight in intellectual and non-intellectually disabled children. *J Intellect Disabil Res*. 2014;58(3):211-20. doi: <http://dx.doi.org/10.1111/j.1365-2788.2012.01615.x>.

28. Stancliffe RJ, Lakin KC, Larson SA, Engler J, Taub S, Fortune J, et al. Demographic characteristics, health conditions, and residential service use in adults with Down syndrome in 25 U.S. states. *Intellect Dev Disabil*. 2012;50(2):92-108. doi: <https://dx.doi.org/10.1352/1934-9556-50.2.92>.

29. Lunsky Y, Durbin A, Brown HK, Bansal S, Heifetz M, Antoniou T. Health profiles and associated service use among adults with HIV and intellectual and developmental disabilities. *Aids*. 2017;31(5):697-705. doi: <https://dx.doi.org/10.1097/QAD.0000000000001361>.

30. Patja K, Eero P, Iivanainen M. Cancer incidence among people with intellectual disability. *J Intellect Disabil Res*. 2001;45(Pt 4):300-7.

31. Sullivan SG, Hussain R, Threlfall T, Bittles AH. The incidence of cancer in people with intellectual disabilities. *Cancer Causes Control*. 2004;15(10):1021-5.

32. Hove O. Weight survey on adult persons with mental retardation living in the community. *Res Dev Disabil*. 2004;25(1):9-17.

33. Simila S, Niskanen P. Underweight and overweight cases among the mentally retarded. *J Ment Defic Res*. 1991;35(Pt 2):160-4.

34. Mikulovic J, Marcellini A, Compte R, Duchateau G, Vanhelst J, Fardy PS, et al. Prevalence of overweight in adolescents with intellectual deficiency. Differences in socio-educative context, physical activity and dietary habits. *Appetite*. 2011;56(2):403-7. doi: <https://dx.doi.org/10.1016/j.appet.2010.12.006>.

35. Carter M, McCaughey E, Annaz D, Hill CM. Sleep problems in a Down syndrome population. *Arch Dis Child*. 2009;94(4):308-10. doi: <https://dx.doi.org/10.1136/adc.2008.146845>.

36. Lin J-D, Yen C-F, Li C-W, Wu J-L. Patterns of Obesity among Children and Adolescents with Intellectual Disabilities in Taiwan. *J Appl Res Intellect Disabil*. 2005;18(2):123-9. doi: <http://dx.doi.org/10.1111/j.1468-3148.2005.00241.x>.

37. Moore KA, McGillivray J, Illingworth K, Brookhouse P. An investigation into the incidence of obesity and underweight among adults with an intellectual disability in an Australian sample. *J Intellect Dev Disabil*. 2004;29(4):306-18. doi: <http://dx.doi.org/10.1080/13668250400014483>.

38. Stancliffe RJ, Lakin KC, Larson S, Engler J, Bershadsky J, Taub S, et al. Overweight and obesity among adults with intellectual disabilities who use intellectual disability/developmental disability services in 20 U.S. States. *Am J Intellect Dev Disabil*. 2011;116(6):401-18. doi: <https://dx.doi.org/10.1352/1944-7558-116.6.401>.

39. Yen CF, Lin JD, Li CW, Wu JL, Lee JT. Body mass index for adults with intellectual disabilities: A survey of caregivers in Taiwan. *J Med Sci*. 2005;25(3):131-7.

40. van de Louw J, Vorstenbosch R, Vinck L, Penning C, Evenhuis H. Prevalence of hypertension in adults with intellectual disability in the Netherlands. *J Intellect Disabil Res*. 2009;53(1):78-84. doi: <https://dx.doi.org/10.1111/j.1365-2788.2008.01130.x>.

41. Tyrer F, Ling S, Bhaumik S, Gangadharan SK, Khunti K, Gray LJ, et al. Diabetes in adults with intellectual disability: prevalence and associated demographic, lifestyle, independence and health factors. *J Intellect Disabil Res*. 2020;64(4):287-95. doi: 10.1111/jir.12718.

42. Benassi G, Guarino M, Cammarata S, Cristoni P, Fantini MP, Ancona A, et al. An epidemiological study on severe mental retardation among schoolchildren in Bologna, Italy. *Dev Med Child Neurol*. 1990;32(10):895-901.

43. Gustavson KH, Hagberg B, Hagberg G, Sars K. Severe mental retardation in a Swedish county I. Epidemiology, gestational age, birth weight and associated CNS handicaps in children born 1959–70. *Acta Pædiatrica*. 1977;66(3):373-9. doi: 10.1111/j.1651-2227.1977.tb07910.x.

44. McQueen PC, Spence MW, Garner JB, Pereira LH, Winsor EJ. Prevalence of major mental retardation and associated disabilities in the Canadian Maritime Provinces. *Am J Ment Defic*. 1987;91(5):460-6.

45. Janicki MP, Maceachron AE. Residential, health, and social service needs of elderly developmentally disabled persons. *The Gerontologist*. 1984;24(2):128. doi: 10.1093/geront/24.2.128.

46. Yen CF, Lin JD, Loh CH, Shi L, Hsu SW. Determinants of prescription drug use by adolescents with intellectual disabilities in Taiwan. *Res Dev Disabil*. 2009;30(6):1354-66. doi: <http://dx.doi.org/10.1016/j.ridd.2009.06.002>.

47. Matthews T, Weston N, Baxter H, Felce D, Kerr M. A general practice-based prevalence study of epilepsy among adults with intellectual disabilities and of its association with psychiatric disorder, behaviour disturbance and carer stress. *J Intellect Disabil Res*. 2008;52(Pt 2):163-73. doi: <https://dx.doi.org/10.1111/j.1365-2788.2007.01025.x>.

48. Morgan CL, Baxter H, Kerr MP. Prevalence of epilepsy and associated health service utilization and mortality among patients with intellectual disability. *Am J Ment Retard*. 2003;108(5):293-300.

49. Forsgren L, Edvinsson SO, Blomquist HK, Heijbel J, Sidenvall R. Epilepsy in a population of mentally retarded children and adults. *Epilepsy Res*. 1990;6(3):234-48.

50. McGrother CW, Bhaumik S, Thorp CF, Hauck A, Branford D, Watson JM. Epilepsy in adults with intellectual disabilities: prevalence, associations and service implications. *Seizure*. 2006;15(6):376-86.

51. Shepherd C, Hosking G. Epilepsy in school children with intellectual impairments in Sheffield: the size and nature of the problem and the implications for service provision. *J Ment Defic Res*. 1989;33(Pt 6):511-4.

52. Lin J-D, Yen C-F, Li C-W, Wu J-L, Chwo M-J, Loh C-H, et al. Epilepsy and health care utilization among people with intellectual disability. *Journal of Disability Research (Taiwan)*. 2003;1:65-77.

53. Lund J. Epilepsy and psychiatric disorder in the mentally retarded adult. *Acta Psychiatr Scand*. 1985;72(6):557-62. doi: <http://dx.doi.org/10.1111/j.1600-0447.1985.tb02654.x>.

54. McCarron M, O'Dwyer M, Burke E, McGlinchey E, McCallion P. Epidemiology of epilepsy in older adults with an intellectual disability in Ireland: associations and service implications. *Am J Intellect Dev Disabil*. 2014;119(3):253-60. doi: <https://dx.doi.org/10.1352/1944-7558-119.3.253>.

55. McGrother C, Hauck A, Bhaumik S, Thorp C, Taub N. Community care for adults with learning disability and their carers: Needs and outcomes from the Leicestershire register. *J Intellect Disabil Res*. 1996;40(2):183-90. doi: <http://dx.doi.org/10.1111/j.1365-2788.1996.tb00621.x>.

56. Warburg M. Visual impairment in adult people with moderate, severe, and profound intellectual disability. *Acta Ophthalmol Scand*. 2001;79(5):450-4. doi: <http://dx.doi.org/10.1034/j.1600-0420.2001.790504.x>.

57. van Splunder J, Stilma JS, Bernsen RM, Arentz TG, Evenhuis HM. Refractive errors and visual impairment in 900 adults with intellectual disabilities in the Netherlands. *Acta Ophthalmol Scand*. 2003;81(2):123-9.

58. Meuwese-Jongejeugd A, Vink M, van Zanten B, Verschuure H, Eichhorn E, Koopman D, et al. Prevalence of hearing loss in 1598 adults with an intellectual disability: cross-sectional population based study. *Int J Audiol*. 2006;45(11):660-9.

59. Pradhan A, Slade GD, Spencer AJ. Factors influencing caries experience among adults with physical and intellectual disabilities. *Community Dent Oral Epidemiol*. 2009;37(2):143-54. doi: <https://dx.doi.org/10.1111/j.1600-0528.2008.00455.x>.

60. Burke É, Carroll R, O’Dwyer M, Walsh JB, McCallion P, McCarron M. Quantitative examination of the bone health status of older adults with intellectual and developmental disability in Ireland: a cross-sectional nationwide study. *BMJ Open*. 2019;9(4):e026939. doi: 10.1136/bmjopen-2018-026939.

61. AlMutairi H, O'Dwyer M, Burke E, McCarron M, McCallion P, Henman MC. Laxative use among older adults with intellectual disability: a cross-sectional observational study. *Int J Clin Pharm*. 2020;42(1):89-99. doi: <http://dx.doi.org/10.1007/s11096-019-00942-z>.

62. Bishop L, McLean KJ, Rubenstein E. Epilepsy in adulthood: Prevalence, incidence, and associated antiepileptic drug use in autistic adults in a state Medicaid system. *Autism*. 2020. doi: <http://dx.doi.org/10.1177/1362361320942982>.

63. Cuypers M, Leijssen M, Bakker-van Gijssel EJ, Pouls KPM, Mastebroek MM, Naaldenberg J, et al. Patterns in the prevalence of diabetes and incidence of diabetic complications in people with and without an intellectual disability in Dutch primary care: Insights from a population-based data-linkage study. *Prim Care Diabetes*. 2021;15(2):372-7. doi: <http://dx.doi.org/10.1016/j.pcd.2020.11.012>.

64. Garcia-Dominguez L, Navas P, Verdugo MA, Arias VB. Chronic Health Conditions in Aging Individuals with Intellectual Disabilities. *Int J Environ Res Public Health*. 2020;17(9):30. doi: <https://dx.doi.org/10.3390/ijerph17093126>.

65. McMahon M, Hatton C. A comparison of the prevalence of health problems among adults with and without intellectual disability: A total administrative population study. *J Appl Res Intellect Disabil*. 2021;34(1):316-25. doi: <http://dx.doi.org/10.1111/jar.12785>.

66. Monaghan R, O'Dwyer M, Luus R, Mulryan N, McCallion P, McCarron M, et al. Antiepileptic drugs, occurrence of seizures and effect of co-administration of potential seizure threshold-lowering psychotropic drugs in adults with intellectual disability who have epilepsy. *J Appl Res Intellect Disabil*. 2021;34(3):818-29. doi: <http://dx.doi.org/10.1111/jar.12857>.

67. O'Brien F, McCallion P, Carroll R, O'Dwyer M, Burke E, McCarron M. The prevalence, awareness, treatment, and control of hypertension in older adults with an intellectual disability in Ireland: a cross sectional study. *Eur J Cardiovasc Nurs*. 2021;04. doi: <http://dx.doi.org/10.1093/eurjcn/zvaa019>.

68. Xie L, Gelfand A, Delclos GL, Atem FD, Kohl HW, 3rd, Messiah SE. Estimated Prevalence of Asthma in US Children With Developmental Disabilities. *JAMA Network Open*. 2020;3(6):e207728. doi: <https://dx.doi.org/10.1001/jamanetworkopen.2020.7728>.

69. Baccichetti C, Lenzini E, Pegoraro R. Down syndrome in the Belluno district (Veneto region, northeast Italy): age distribution and morbidity. *Am J Med Genet Suppl*. 1990;7:84-6.

70. Henderson A, Lynch SA, Wilkinson S, Hunter M. Adults with Down's sydrome: The prevalence of complications and health care in the community. *Br J Gen Pract*. 2007;57(534):50-5.

71. Kapell D, Nightingale B, Rodriguez A, Lee JH, Zigman WB, Schupf N. Prevalence of chronic medical conditions in adults with mental retardation: comparison with the general population. *Ment Retard*. 1998;36(4):269-79.

72. Leonard S, Bower C, Petterson B, Leonard H. Medical aspects of school‐aged children with Down syndrome. *Dev Med Child Neurol*. 1999;41(10):683-8. doi: 10.1111/j.1469-8749.1999.tb00523.x.

73. Määttä T, Määttä J, Tervo-Määttä T, Taanila A, Kaski M, Iivanainen M. Healthcare and guidelines: A population-based survey of recorded medical problems and health surveillance for people with Down syndrome. *J Intellect Dev Disabil*. 2011;36(2):118-26. doi: 10.1080/13668250.2011.570253.

74. McGrother CW, Marshall B. Recent trends in incidence, morbidity and survival in Down's syndrome. *J Ment Defic Res*. 1990;34(Pt 1):49-57.

75. Pikora TJ, Bourke J, Bathgate K, Foley K-R, Lennox N, Leonard H. Health conditions and their impact among adolescents and young adults with Down syndrome. *PLoS ONE*. 2014;9(5):e96868.

76. Prasher VP, Glenn S, Cunningham C, Arshad H, Glenholmes P, Kirby A. Health morbidity and access to services by young adults with Down syndrome. *Int J Dev Disabil*. 2014;60(1):26-34. doi: <http://dx.doi.org/10.1179/204738713X.13673354444083>.

77. Roizen NJ, Magyar CI, Kuschner ES, Sulkes SB, Druschel C, Van Wijngaarden E, et al. A community cross-sectional survey of medical problems in 440 children with down syndrome in New York state. *Journal of Pediatrics*. 2014;164(4):871-5. doi: <http://dx.doi.org/10.1016/j.jpeds.2013.11.032>.

78. Thomas K, Bourke J, Girdler S, Bebbington A, Jacoby P, Leonard H. Variation over time in medical conditions and health service utilization of children with Down syndrome. *J Pediatr*. 2011;158(2):194-200.e1. doi: <https://dx.doi.org/10.1016/j.jpeds.2010.08.045>.

79. Startin CM, D’Souza H, Ball G, Hamburg S, Hithersay R, Hughes KMO, et al. Health comorbidities and cognitive abilities across the lifespan in Down syndrome. *J Neurodev Disord*. 2020;12(1):4. doi: 10.1186/s11689-019-9306-9.

80. Arnell H, Fischler B. Population-based study of incidence and clinical outcome of neonatal cholestasis in patients with Down syndrome. *J Pediatr*. 2012;161(5):899-902. doi: <https://dx.doi.org/10.1016/j.jpeds.2012.04.037>.

81. Weijerman ME, van Furth AM, van der Mooren MD, van Weissenbruch MM, Rammeloo L, Broers CJ, et al. Prevalence of congenital heart defects and persistent pulmonary hypertension of the neonate with Down syndrome. *Eur J Pediatr*. 2010;169(10):1195-9. doi: <https://dx.doi.org/10.1007/s00431-010-1200-0>.

82. Fabia J, Drolette M. Malformations and leukemia in children with Down's syndrome. *Pediatrics*. 1970;45(1):60.

83. Hamilton J, Yaneza MM, Clement WA, Kubba H. The prevalence of airway problems in children with Down's syndrome. *Int J Pediatr Otorhinolaryngol*. 2016;81:1-4. doi: <https://dx.doi.org/10.1016/j.ijporl.2015.11.027>.

84. Jaruratanasirikul S, Limpitikul W, Dissaneevate P, Booncharoen P, Tantichantakarun P. Comorbidities in Down syndrome livebirths and health care intervention: an initial experience from the birth defects registry in Southern Thailand. *World J Pediatr*. 2017;13(2):152-7. doi: <https://dx.doi.org/10.1007/s12519-016-0093-z>.

85. Garg A, Strunk A, Midura M, Papagermanos V, Pomerantz H. Prevalence of hidradenitis suppurativa among patients with Down syndrome: a population-based cross-sectional analysis. *Br J Dermatol*. 2018;178(3):697-703. doi: <http://dx.doi.org/10.1111/bjd.15770>.

86. Haugen OH, Hovding G. Strabismus and binocular function in children with Down syndrome. A population-based, longitudinal study. *Acta Ophthalmol Scand*. 2001;79(2):133-9. doi: <http://dx.doi.org/10.1034/j.1600-0420.2001.079002133.x>.

87. Boker LK, Blumstein T, Sadetzki S, Luxenburg O, Litvak I, Akstein E, et al. Incidence of leukemia and other cancers in Down syndrome subjects in Israel. *Int J Cancer*. 2001;93(5):741-4.

88. Hasle H, Friedman JM, Olsen Jo H, Rasmussen SA. Low risk of solid tumors in persons with Down syndrome. *Genet Med*. 2016;18(11):1151-7. doi: <http://dx.doi.org/10.1038/gim.2016.23>.

89. Bjorge T, Cnattingius S, Lie RT, Tretli S, Engeland A. Cancer risk in children with birth defects and in their families: A population based cohort study of 5.2 million children from Norway and Sweden. *Cancer Epidemiol Biomarkers Prev*. 2008;17(3):500-6. doi: <http://dx.doi.org/10.1158/1055-9965.EPI-07-2630>.

90. Patja K, Pukkala E, Sund R, Iivanainen M, Kaski M. Cancer incidence of persons with Down syndrome in Finland: a population-based study. *Int J Cancer*. 2006;118(7):1769-72.

91. Sullivan SG, Hussain R, Glasson EJ, Bittles AH. The profile and incidence of cancer in Down syndrome. *J Intellect Disabil Res*. 2007;51(Pt 3):228-31.

92. Murphy J, Philip M, Macken S, Meehan J, Roche E, Mayne PD, et al. Thyroid dysfunction in Down's syndrome and screening for hypothyroidism in children and adolescents using capillary TSH measurement. *J Pediatr Endocrinol Metab*. 2008;21(2):155-63.

93. Melville CA, Cooper SA, McGrother CW, Thorp CF, Collacott R. Obesity in adults with Down syndrome: a case-control study. *J Intellect Disabil Res*. 2005;49(Pt 2):125-33.

94. Prasher VP. Overweight and obesity amongst Down's syndrome adults. *J Intellect Disabil Res*. 1995;39(5):437-41. doi: 10.1111/j.1365-2788.1995.tb00548.x.

95. Carfi A, Vetrano DL, Mascia D, Meloni E, Villani ER, Acampora N, et al. Adults with Down syndrome: a comprehensive approach to manage complexity. *J Intellect Disabil Res*. 2019;63(6):624-9. doi: 10.1111/jir.12588.

96. Johannsen P, Christensen JE, Goldstein H, Nielsen VK, Mai J. Epilepsy in Down syndrome--prevalence in three age groups. *Seizure*. 1996;5(2):121-5.

97. Barr E, Dungworth J, Hunter K, McFarlane M, Kubba H. The prevalence of ear, nose and throat disorders in preschool children with Down's syndrome in Glasgow. *Scott Med J*. 2011;56(2):98-103. doi: <https://dx.doi.org/10.1258/smj.2011.011036>.

98. Yaneza MM, Hunter K, Irwin S, Kubba H. Hearing in school-aged children with trisomy 21 - results of a longitudinal cohort study in children identified at birth. *Clin Otolaryngol*. 2016;41(6):711-7. doi: <https://dx.doi.org/10.1111/coa.12606>.

99. Austeng ME, Akre H, Overland B, Abdelnoor M, Falkenberg ES, Kvaerner KJ. Otitis media with effusion in children with in Down syndrome. *Int J Pediatr Otorhinolaryngol*. 2013;77(8):1329-32. doi: <https://dx.doi.org/10.1016/j.ijporl.2013.05.027>.

100. Austeng ME, Akre H, Falkenberg E-S, Overland B, Abdelnoor M, Kvaerner KJ. Hearing level in children with Down syndrome at the age of eight. *Res Dev Disabil*. 2013;34(7):2251-6. doi: <http://dx.doi.org/10.1016/j.ridd.2013.04.006>.

101. Park AH, Wilson MA, Stevens PT, Harward R, Hohler N. Identification of hearing loss in pediatric patients with Down syndrome. *Otolaryngol Head Neck Surg*. 2012;146(1):135-40. doi: <https://dx.doi.org/10.1177/0194599811425156>.

102. Jansson U, Johansson C. Down syndrome and celiac disease. *J Pediatr Gastroenterol Nutr*. 1995;21(4):443-5.

103. Brodwall K, Greve G, Leirgul E, Klungsoyr K, Holmstrom H, Vollset SE, et al. The five-year survival of children with Down syndrome in Norway 1994-2009 differed by associated congenital heart defects and extracardiac malformations. *Acta Paediatr*. 2018;107(5):845-53. doi: <http://dx.doi.org/10.1111/apa.14223>.

104. Torfs CP, Christianson RE. Anomalies in Down syndrome individuals in a large population-based registry. *Am J Med Genet*. 1998;77(5):431-8. doi: <http://dx.doi.org/10.1002/%28SICI%291096-8628%2819980605%2977:5%3C431::AID-AJMG15%3E3.0.CO;2-J>.

105. Kupferman JC, Druschel CM, Kupchik GS. Increased prevalence of renal and urinary tract anomalies in children with Down syndrome. *Pediatrics*. 2009;124(4):e615-21. doi: <https://dx.doi.org/10.1542/peds.2009-0181>.

106. Bergström S, Carr H, Petersson G, Stephansson O, Bonamy A-KE, Dahlström A, et al. Trends in Congenital Heart Defects in Infants With Down Syndrome. *Pediatrics*. 2016;138(1). doi: 10.1542/peds.2016-0123.

107. Freeman SB, Bean LH, Allen EG, Tinker SW, Locke AE, Druschel C, et al. Ethnicity, sex, and the incidence of congenital heart defects: a report from the National Down Syndrome Project. *Genet Med*. 2008;10(3):173-80. doi: <https://dx.doi.org/10.1097/GIM.0b013e3181634867>.

108. Freeman SB, Taft LF, Dooley KJ, Allran K, Sherman SL, Hassold TJ, et al. Population-based study of congenital heart defects in Down syndrome. *Am J Med Genet*. 1998;80(3):213. doi: 2-8.

109. Kim MA, Lee YS, Yee NH, Choi JS, Choi JY, Seo K. Prevalence of congenital heart defects associated with Down syndrome in Korea. *J Korean Med Sci*. 2014;29(11):1544-9. doi: <https://dx.doi.org/10.3346/jkms.2014.29.11.1544>.

110. Santoro M, Coi A, Spadoni I, Bianchi F, Pierini A. Sex differences for major congenital heart defects in Down Syndrome: A population based study. *Eur J Med Genet*. 2018;61(9):546-50. doi: <http://dx.doi.org/10.1016/j.ejmg.2018.05.013>.

111. Scott C, Thame M. The incidence of cardiac lesions among children with Down's syndrome in Jamaica - A prospective study. *West Indian Med J*. 2014;63(7):693-7. doi: <http://dx.doi.org/10.7727/wimj.2013.216>.

112. So SA, Urbano RC, Hodapp RM. Hospitalizations of infants and young children with Down syndrome: evidence from inpatient person-records from a statewide administrative database. *J Intellect Disabil Res*. 2007;51(Pt 12):1030-8.

113. Irving CA, Chaudhari MP. Cardiovascular abnormalities in Down's syndrome: spectrum, management and survival over 22 years. *Arch Dis Child*. 2012;97(4):326-30. doi: <https://dx.doi.org/10.1136/adc.2010.210534>.

114. Freeman SB, Torfs CP, Romitti PA, Royle MH, Druschel C, Hobbs CA, et al. Congenital gastrointestinal defects in Down syndrome: a report from the Atlanta and National Down Syndrome Projects. *Clinical genetics*. 2009;75(2):180-4. doi: 10.1111/j.1399-0004.2008.01110.x.

115. Tedeschi AS, Roizen NJ, Taylor HG, Murray G, Curtis CA, Parikh AS. The prevalence of congenital hearing loss in neonates with Down syndrome. *J Pediatr*. 2015;166(1):168-71. doi: <https://dx.doi.org/10.1016/j.jpeds.2014.09.005>.

116. Cho WK, Lee NY, Han K, Suh BK, Park YG. The population prevalence, associations of congenital heart defect and mortality risk for down's syndrome in South Korea based on national health insurance service (NHIS) data. *Clin Epidemiol*. 2020;12:519-25. doi: <http://dx.doi.org/10.2147/CLEP.S251637>.

117. Kristianslund O, Drolsum L. Prevalence of Keratoconus in Persons with down Syndrome in a National Registry in Norway. *JAMA Network Open*. 2021;4(3):e210814. doi: <http://dx.doi.org/10.1001/jamanetworkopen.2021.0814>.

118. Ostermaier KK, Weaver AL, Myers SM, Stoeckel RE, Katusic SK, Voigt RG. Incidence of Celiac Disease in Down Syndrome: A Longitudinal, Population-Based Birth Cohort Study. *Clin Pediatr*. 2020;59(12):1086-91. doi: <https://dx.doi.org/10.1177/0009922820941247>.

119. Schultz-Pedersen S, Hasle H, Olsen JH, Friedrich U. Evidence of decreased risk of cancer in individuals with fragile X. *Am J Med Genet*. 2001;103(3):226-30.

120. Sund R, Pukkala E, Patja K. Cancer incidence among persons with fragile X syndrome in Finland: a population-based study. *J Intellect Disabil Res*. 2009;53(1):85-90. doi: <https://dx.doi.org/10.1111/j.1365-2788.2008.01116.x>.

121. Sabaratnam M, Vroegop PG, Gangadharan SK. Epilepsy and EEG findings in 18 males with fragile X syndrome. *Seizure*. 2001;10(1):60-3.

122. Butler JV, Whittington JE, Holland AJ, Boer H, Clarke D, Webb T. Prevalence of, and risk factors for, physical ill-health in people with Prader-Willi syndrome: a population-based study. *Dev Med Child Neurol*. 2002;44(4):248-55.

123. Diene G, Mimoun E, Feigerlova E, Caula S, Molinas C, Grandjean H, et al. Endocrine disorders in children with Prader-Willi syndrome--data from 142 children of the French database. *Horm Res Paediatr*. 2010;74(2):121-8. doi: <https://dx.doi.org/10.1159/000313377>.

124. Patja K, Sund R, Kaski M, Pukkala E. Cancer incidence among persons with Prader-Willi syndrome in Finland. *Int J Disabil Hum Dev*. 2008;7(1):69-72. doi: <http://dx.doi.org/10.1515/IJDHD.2008.7.1.69>.

125. Downs J, Bebbington A, Woodhead H, Jacoby P, Jian L, Jefferson A, et al. Early determinants of fractures in Rett syndrome. *Pediatrics*. 2008;121(3):540-6. doi: <https://dx.doi.org/10.1542/peds.2007-1641>.

126. Freilinger M, Bohm M, Lanator I, Vergesslich-Rothschild K, Huber WD, Anderson A, et al. Prevalence, clinical investigation, and management of gallbladder disease in Rett syndrome. *Dev Med Child Neurol*. 2014;56(8):756-62. doi: <https://dx.doi.org/10.1111/dmcn.12358>.

127. Downs J, Torode I, Wong K, Ellaway C, Elliott EJ, Christodoulou J, et al. The Natural History of Scoliosis in Females With Rett Syndrome. *Spine*. 2016;41(10):856-63. doi: 10.1097/BRS.0000000000001399.

128. Hjortshoj TD, Gronskov K, Rosenberg T, Brondum-Nielsen K, Olsen JH. Risk for cancer in patients with Bardet-Biedl syndrome and their relatives. *Am J Med Genet A*. 2007;143A(15):1699-702.

129. Thomson AK, Glasson EJ, Bittles AH. A long-term population-based clinical and morbidity profile of Angelman syndrome in Western Australia: 1953-2003. *Disabil Rehabil*. 2006;28(5):299-305. doi: <http://dx.doi.org/10.1080/09638280500190631>.

130. Zarchi O, Attias J, Raveh E, Basel-Vanagaite L, Saporta L, Gothelf D. A comparative study of hearing loss in two microdeletion syndromes: velocardiofacial (22q11.2 deletion) and Williams (7q11.23 deletion) syndromes. *J Pediatr*. 2011;158(2):301-6. doi: <https://dx.doi.org/10.1016/j.jpeds.2010.07.056>.
