# Supplementary material for: Prevalence and incidence of physical health conditions in people with intellectual disability – a systematic review
Source: PLoS One. 2021 Aug 24;16(8):e0256294. doi: 10.1371/journal.pone.0256294 (PMC8384165; doi:10.1371/journal.pone.0256294)
Supplement: S3 File — (DOCX) [file pone.0256294.s003.docx]

Table S8. Study quality assessment scores

| Author (publication year) | Outcomes to which it applied | Representativeness | Non-ID selection* | Response rates/follow-up | ID ascertainment | No outcome at the start | Comparability 1 (morbidity) | Comparability 2 (relative risks) * | Outcome assessment | Statistical model* | Sum 1^ | Sum 2^ |
| --- | --- | --- | --- | --- | --- | --- | --- | --- | --- | --- | --- | --- |
| Arnell et al. (2012) | All outcomes | 2 |  | 2 | 1 |  | 1 |  | 1 |  | 7 | N/A |
| Arvio et al. (2003) | All outcomes | 1 |  | 2 | 1 |  | 0 |  | 1 |  | 5 | N/A |
| Ashman et al.(1996) | All outcomes | 2 |  | 1 | 0 |  | 1 |  | 0 |  | 4 | N/A |
| Atladottir et al. (2015) | All outcomes | 2 | 1 | 2 | 1 |  | 1 | 1 | 1 | 0 | 7 | 8 |
| Austeng et al. (2013) | All outcomes | 1 | 0 | 1 | 1 |  | 1 | 1 | 1 | 0 | 5 | 5 |
| Austeng et al. (2013) | All outcomes | 1 | 0 | 1 | 1 |  | 1 | 1 | 1 | 0 | 5 | 5 |
| Austeng et al. (2013) | Specific hearing loss | 1 |  | 1 | 1 |  | 1 |  | 1 |  | 5 | N/A |
| Baccichetti et al. (1990) | All outcomes | 2 |  | 1 | 1 |  | 0 |  | 0 |  | 4 | N/A |
| Murphy et al. (2008) | All outcomes | 2 |  | 0 | 0 |  | 1 |  | 1 |  | 4 | N/A |
| Barr et al. (2011) | All outcomes | 2 |  | 1 | 1 |  | 1 |  | 1 |  | 6 | N/A |
| Benassi et al. (1990) | All outcomes | 2 |  | 2 | 1 |  | 1 |  | 1 |  | 7 | N/A |
| Bergström et al. (2016) | All outcomes | 2 |  | 2 | 1 |  | 1 |  | 1 |  | 7 | N/A |
| Bhaumik et al. (2007) | Obesity | 1 | 0 | 1 | 0 |  | 1 | 1 | 1 | 1 | 4 | 5 |
| Bhaumik et al. (2007) | Epilepsy | 1 |  | 1 |  |  | 1 | 1 | 0 |  | 3 | N/A |
| Bhaumik et al. (2007) | Other outcomes | 1 |  | 1 |  |  | 1 | 1 | 1 |  | 4 | N/A |
| Bjørge et al. (2008) | All outcomes | 2 | 1 | 2 | 1 | 1 | 0 | 1 | 1 | 1 | 7 | 10 |
| Boker et al. (2001) | All outcomes | 2 | 0 | 2 | 1 | 1 | 1 | 1 | 1 | 1 | 8 | 9 |
| Boyle et al. (2010) | All outcomes | 2 |  | 2 | 1 |  | 0 |  | 1 |  | 6 | N/A |
| Brodwall et al. (2018) | All outcomes | 2 | 1 | 2 | 1 |  | 1 | 1 | 1 | 1 | 7 | 9 |
| Burke et al. (2017) | Osteoporosis | 2 |  | 0 | 0 |  | 2 |  | 0 |  | 4 | N/A |
| Burke et al. (2017) | Other outcomes | 2 |  | 0 | 0 |  | 1 |  | 0 |  | 3 | N/A |
| Butler et al. (2002) | All outcomes | 1 |  | 1 | 1 |  | 1 |  | 0 |  | 4 | N/A |
| Carey et al. (2016) | All outcomes | 2 | 1 | 2 | 1 |  | 1 | 2 | 1 | 1 | 7 | 10 |
| Carter et al. (2009) | All outcomes | 2 |  | 1 | 0 |  | 1 |  | 0 |  | 4 | N/A |
| Christianson et al. (2002) | All outcomes | 2 |  | 2 | 1 |  | 1 |  |  |  | 7 | N/A |
| Cooper et al. (2018) | All outcomes | 2 | 0 | 1 | 1 |  | 1 | 1 | 1 | 1 | 6 | 7 |
| Cooper et al. (2015) | All outcomes | 2 | 1 | 2 | 1 |  | 1 | 2 | 1 | 1 | 7 | 10 |
| Diene et al. (2010) | All outcomes | 2 |  | 2 | 1 |  | 1 |  | 1 |  | 7 | N/A |
| Downs et al. (2008) | All outcomes | 0 | 0 | 1 | 1 |  | 1 |  | 0 | 1 | 3 | N/A |
| Downs et al. (2016) | All outcomes | 0 |  | 0 | 1 | 1 | 1 |  | 0 |  | 3 | N/A |
| Fabia et al. (1970) | All outcomes | 2 |  | 2 | 1 |  | 1 |  | 1 |  | 7 | N/A |
| Finlayson et al. (2010) | Injuries (self-injury excluded) | 2 | 0 | 2 | 1 | 1 | 1 | 1 | 0 | 1 | 7 | 8 |
| Finlayson et al. (2010) | Other outcomes | 2 |  | 2 | 1 |  | 1 |  | 1 |  | 7 | N/A |
| Folch-Mas et al. (2017) | All outcomes | 2 |  | 0 | 0 |  | 1 |  | 0 |  | 3 | N/A |
| Forsgren et al., 1990 | All outcomes | 2 |  | 2 | 1 |  | 0 |  | 0 |  | 5 | N/A |
| Freeman et al. (2008) | All outcomes | 2 |  | 2 | 1 |  | 1 |  | 1 |  | 7 | N/A |
| Freeman et al. (1998) | All outcomes | 2 |  | 2 |  |  | 1 |  | 1 |  | 7 | N/A |
| Freeman et al. (2009) | All outcomes | 2 |  | 2 | 1 |  | 1 |  | 1 |  | 7 | N/A |
| Freilinger et al. (2014) | All outcomes | 2 |  | 0 | 0 | 0 | 0 |  | 0 |  | 2 | N/A |
| Gale et al. (2009) | Asthma | 2 |  | 2 | 1 |  | 1 |  | 1 |  | 7 | N/A |
| Gale et al. (2009) | Other outcomes | 2 |  | 0 | 1 |  | 1 |  | 1 |  | 5 | N/A |
| Garg et al. (2018) | Hidradenitis suppurativa | 1 | 1 | 2 | 1 |  | 1 | 2 | 1 | 1 | 6 | 9 |
| Garg et al. (2018) | Obesity | 1 |  | 2 | 1 |  | 1 |  | 1 |  | 6 | N/A |
| Gustavson et al. (1977) | All outcomes | 2 |  | 2 | 1 |  | 1 |  | 1 |  | 7 | N/A |
| Hamilton et al. (2016) | All outcomes | 2 |  | 2 | 1 |  | 1 |  | 1 |  | 7 | N/A |
| Hand et al. (1996) | All outcomes | 1 |  | 2 | 0 |  | 1 |  | 0 |  | 4 | N/A |
| Hasle et al. (2016) | All sites | 2 | 0 | 2 | 1 | 1 | 1 | 1 | 1 | 1 | 8 | 9 |
| Hasle et al. (2016) | All solid tumors | 2 | 0 | 2 | 1 | 1 | 1 | 1 | 1 | 1 | 8 | 9 |
| Hasle et al. (2016) | All lymphomas and leukemia | 2 | 0 | 2 | 1 | 1 | 1 | 1 | 1 | 1 | 8 | 9 |
| Hasle et al. (2016) | Other specific cancers | 2 | 0 | 2 | 1 | 1 | 0 | 1 | 1 | 1 | 7 | 9 |
| Haugen et al. (2001) | All outcomes | 1 |  | 0 | 1 | 0 | 1 |  | 1 |  | 4 | N/A |
| Haveman et al. (2011) | All outcomes | 1 |  | 0 | 0 |  | 1 |  | 0 |  | 2 | N/A |
| Henderson et al. (2007) | Congenital heart defects | 1 |  | 2 | 1 |  | 1 |  | 1 |  | 6 | N/A |
| Hjortshøj et al. (2007) | Cancers at all sites | 2 | 0 | 2 | 1 | 1 | 0 | 1 | 1 | 1 | 7 | 9 |
| Hove et al. (2004) | All outcomes (for ID) | 1 |  | 1 | 1 |  | 2 |  | 1 |  | 6 | N/A |
| Hove et al. (2004) | All outcomes (for DS) |  |  |  |  |  | 0 |  | 1 |  | 4 | N/A |
| Irving et al. (2012) | Cardiovascular anomaly | 2 |  | 2 | 0 |  | 1 |  | 1 |  | 6 | N/A |
| Janicki et al. (1984) | All outcomes | 1 |  | 2 | 0 |  | 2 |  | 0 |  | 5 | N/A |
| Jansson et al. (1995) | All outcomes | 2 |  | 1 | 0 |  | 1 |  | 1 |  | 5 | N/A |
| Jaruratanasirikul et al. (2017) | All outcomes | 2 |  | 2 | 1 |  | 1 |  | 1 |  | 7 | N/A |
| Johannsen et al. (1996) | All outcomes | 2 |  | 2 | 1 |  | 1 |  | 0 |  | 6 | N/A |
| Kapell et al. (1998) | All outcomes | 1 | 0 | 2 | 0 |  | 2 | 1 | 1 | 1 | 6 | 6 |
| Kim et al. (2014) | All outcomes | 2 |  | 2 | 1 |  | 1 |  | 1 |  | 7 | N/A |
| Kinnear et al. (2018) | All outcomes | 2 |  | 0 | 1 |  | 0 |  | 1 |  | 4 | N/A |
| Kupferman et al. (2009) | Renal and urinary tract anomalies | 2 | 1 | 2 | 1 |  | 0 | 0 | 1 | 0 | 6 | 7 |
| Kupferman et al. (2009) | Other outcomes |  |  |  |  |  |  |  |  |  | 6 | N/A |
| Leonard et al. (1999) | Congenital heart defects (4 outcomes involved) | 2 |  | 2 | 1 |  | 1 |  | 1 |  | 7 | N/A |
| Leonard et al. (1999) | Other outcomes |  |  |  |  |  |  |  | 0 |  | 6 | N/A |
| Lin et al. (2006) | All outcomes | 1 |  | 0 | 1 |  | 0 |  | 0 |  | 2 | N/A |
| Lin et al. (2005) | All outcomes | 1 |  | 0 | 1 |  | 1 |  | 0 |  | 3 | N/A |
| Lin et al. (2003) | All outcomes | 1 |  | 0 | 1 |  | 2 |  | 0 |  | 4 | N/A |
| Lund (1985) | All outcomes | 1 |  | 1 | 0 |  | 2 |  | 0 |  | 4 | N/A |
| Lunsky et al. (2017) | All outcomes | 2 | 1 | 2 | 1 |  | 1 | 1 | 1 | 1 | 7 | 9 |
| Määttä et al. (2011) | All outcomes | 1 |  | 2 | 1 |  | 0 |  | 1 |  | 5 | N/A |
| Matthews et al. (2008) | All outcomes | 2 |  | 1 | 1 |  | 1 |  | 1 |  | 6 | N/A |
| McCarron et al. (2017) | All outcomes | 2 | 1 | 1 | 0 |  | 1 | 2 | 0 | 0 | 4 | 6 |
| McCarron et al. (2014) | All outcomes | 2 |  | 0 | 0 |  | 2 |  | 0 |  | 4 | N/A |
| McCarron et al. (2013) | All outcomes | 2 |  | 1 | 0 |  | 1 |  | 0 |  | 4 | N/A |
| McGrother et al. (1996) | All outcomes | 2 |  | 1 |  |  | 1 |  | 0 |  | 4 | N/A |
| McGrother et al. (2006) | All outcomes | 2 |  | 2 | 0 |  | 1 |  | 0 |  | 5 | N/A |
| McGrother et al. (1990) | All outcomes | 2 |  | 2 | 1 |  | 1 |  | 1 |  | 7 | N/A |
| McQueen et al. (1987) | All outcomes | 2 |  | 2 | 1 |  | 1 |  | 0 |  | 6 | N/A |
| Melville et al. (2005) | All outcomes | 2 |  | 1 | 1 |  | 1 |  | 1 |  | 6 | N/A |
| Melville et al. (2008) | Obesity (unspecified) | 2 | 0 | 1 | 1 |  | 1 | 1 | 1 | 0 | 6 | 6 |
| Melville et al. (2008) | Other outcomes | 2 |  | 1 | 1 |  | 1 |  | 1 |  | 6 | N/A |
| Jongejeugd et al. (2006) | All outcomes | 1 |  | 0 | 1 |  | 2 |  | 1 |  | 5 | N/A |
| Mikulovic et al. (2011) | All outcomes | 1 |  | 1 | 1 |  | 1 |  | 1 |  | 5 | N/A |
| Moore et al. (2004) | All outcomes | 1 |  | 0 | 0 |  | 1 |  | 1 |  | 3 | 3 |
| Morgan et al. (2003) | All outcomes | 2 | 0 | 2 | 1 |  | 0 | 1 | 1 | 1 | 6 | 8 |
| Morin et al. (2012) | All outcomes | 1 | 0 | 0 | 0 |  | 2 |  | 0 | 0 | 3 | 3 |
| Park et al. (2012) | Hearing loss | 2 |  | 1 | 1 |  | 1 |  | 1 |  | 6 | N/A |
| Patja et al. (2001) | All outcomes | 2 | 0 | 2 | 1 | 0 | 1 | 1 | 1 | 1 | 7 | 8 |
| Patja et al. (2006) | Cancer (including unspecified leukemia) | 1 | 0 | 2 | 1 | 0 | 1 | 1 | 1 | 1 | 6 | 7 |
| Patja et al. (2006) | Specific cancers (excluding unspecified leukemia) | 1 | 0 | 2 | 1 | 0 | 0 | 1 | 1 | 1 | 5 | 7 |
| Patja et al. (2008) | All outcomes | 1 | 0 | 2 | 1 | 0 | 1 | 1 | 1 | 1 | 6 | 7 |
| Pikora et al. (2014) | All outcomes | 2 |  | 1 | 0 |  | 1 |  | 0 |  | 4 | N/A |
| Pradhan et al. (2009) | All outcomes | 2 |  | 1 | 0 |  | 1 |  | 0 |  | 4 | N/A |
| Prasher et al. (1995) | All outcomes | 1 |  | 0 | 1 |  | 1 |  | 1 |  | 4 | N/A |
| Prasher et al. (2014) | All outcomes | 2 |  | 1 | 1 |  | 1 |  | 0 |  | 5 | N/A |
| Richdale et al. (2000) | All outcomes | 1 | 0 | 0 | 0 |  | 1 |  | 0 | 0 | 2 | 2 |
| Roizen et al. (2014) | All outcomes | 2 |  | 0 | 1 |  | 1 |  | 0 |  | 4 | N/A |
| Sabaratnam et al. (2001) | All outcomes | 1 |  | 2 | 1 |  | 0 |  | 0 |  | 4 | N/A |
| Santoro et al. (2018) | All outcomes | 2 |  | 2 | 1 |  | 1 |  | 1 |  | 7 | N/A |
| Schultz-Pedersen et al. (2001) | All outcomes | 2 | 0 | 2 | 1 | 1 | 1 | 1 | 1 | 1 | 8 | 9 |
| Scott et al. (2014) | All outcomes | 2 |  | 2 | 1 |  | 1 |  | 1 |  | 7 | N/A |
| Segal et al. (2016) | Obesity | 2 | 1 | 0 | 0 |  | 1 | 2 | 0 | 1 | 3 | 6 |
| Segal et al. (2016) | All outcomes | 2 | 1 | 0 | 0 |  | 1 | 1 | 0 | 1 | 3 | 5 |
| Shepherd et al. (1989) | All outcomes | 1 |  | 2 | 0 |  | 1 |  | 1 |  | 5 | N/A |
| Simila et al. (1991) | All outcomes | 2 |  | 1 | 0 |  | 2 |  | 1 |  | 6 | N/A |
| Slevin et al. (2014) | Overweight / obese | 1 | 1 | 1 | 0 |  | 1 | 1 | 1 | 0 | 4 | 5 |
| Slevin et al. (2014) | Epilepsy | 1 |  | 0 | 0 |  | 1 |  | 0 | 0 | 2 | N/A |
| So et al. (2007) | All outcomes | 2 |  | 2 | 1 |  | 1 |  | 1 |  | 7 | N/A |
| Stancliffe et al. (2012) | All outcomes | 1 |  | 0 | 0 |  | 2 |  | 0 |  | 3 | N/A |
| Stancliffe et al. (2011) | All outcomes | 1 |  | 1 | 0 |  | 1 |  | 0 |  | 3 | N/A |
| Sullivan et al. (2007) | All outcomes | 1 | 0 | 2 | 1 | 0 | 1 | 1 | 1 | 1 | 6 | 7 |
| Sullivan et al. (2004) | All outcomes | 1 | 0 | 2 | 1 | 0 | 1 | 1 | 1 | 1 | 6 | 7 |
| Sund et al. (2009) | All outcomes | 1 | 0 | 2 | 0 | 0 | 1 | 1 | 1 | 1 | 5 | 6 |
| Tedeschi et al. (2015) | All outcomes | 1 |  | 1 | 1 |  | 1 |  | 1 |  | 5 | N/A |
| Thomas et al. (2011) | All outcomes | 1 |  | 1 | 1 |  | 1 |  | 0 |  | 4 | N/A |
| Thomson et al. (2006) | All outcomes | 1 |  | 2 | 1 |  | 0 |  | 1 |  | 5 | N/A |
| Torfs et al. (1998) | All outcomes | 2 | 1 | 2 | 1 |  | 1 | 1 | 1 | 1 | 7 | 9 |
| Van de Louw et al. (2009) | All outcomes | 1 |  | 0 | 1 |  | 1 |  | 1 |  | 4 | N/A |
| van Splunder et al. (2003) | Refractive error | 1 |  | 0 | 1 |  | 0 |  | 1 |  | 3 | N/A |
| van Splunder et al. (2003) | Visual impairment | 1 |  | 2 | 1 |  | 0 |  | 1 |  | 4 | N/A |
| Wee et al. (2014) | All outcomes | 1 |  | 1 | 0 |  | 1 |  | 1 |  | 4 | N/A |
| Weijerman et al. (2010) | Congenital heart defects | 2 | 0 | 0 | 1 |  | 1 | 1 | 1 | 0 | 5 | 5 |
| Weijerman et al. (2010) | Congenital heart defects (4 outcomes involved) | 2 |  | 0 | 1 |  | 1 |  | 1 |  | 5 | N/A |
| Weijerman et al. (2010) | Persistent pulmonary hypertension of the neonate | 2 | 0 | 0 | 1 |  | 1 | 1 | 1 | 0 | 5 | 5 |
| Wellesley et al. (1992) | Cerebral palsy and epilepsy | 2 |  | 2 | 1 |  | 2 |  | 1 |  | 8 | N/A |
| Wellesley et al. (1992) | Visual/hearing impairment | 2 |  | 2 | 1 |  | 1 |  | 1 |  | 7 | N/A |
| Yaneza et al. (2016) | All outcomes | 2 |  | 2 | 0 |  | 1 |  | 1 |  | 6 | N/A |
| Yen et al. (2005) | All outcomes | 2 |  | 0 | 0 |  | 1 |  | 0 |  | 3 | N/A |
| Yen et al. (2009) | All outcomes | 2 |  | 0 | 1 |  | 1 |  | 0 |  | 4 | N/A |
| Zarchi et al. (2011) | All outcomes | 2 | 0 | 0 | 1 |  | 0 | 0 | 1 | 0 | 4 | 4 |
| Beange et al. (1995) | All outcomes | 1 | 0 | 2 | 1 |  | 1 | 1 | 1 | 0 | 6 | 6 |
| Warburg (2001) | Eye disorders | 1 |  | 1 | 0 |  | 1 |  | 1 |  | 4 | N/A |
| Warburg (2001) | Visual impairment | 1 |  | 0 |  |  | 1 |  | 1 |  | 3 | N/A |
| Tyrer et al. (2020) | All outcomes | 2 |  | 1 | 0 |  | 1 |  | 0 |  | 4 | N/A |
| Burke et al. (2019) | All outcomes | 2 |  | 0 | 0 |  | 2 |  | 1 |  | 5 | N/A |
| Startin et al. (2020) | Cerebral palsy | 1 |  | 1 | 1 |  | 1 |  | 0 |  | 4 | N/A |
| Carfi et al. (2019) | Overweight | 1 |  | 0 | 1 |  | 1 |  | 0 |  | 3 | N/A |
| AlMutairi (2020) | Constipation | 2 |  | 0 | 0 |  | 1 |  | 0 |  | 3 | N/A |
| Bishop (2020) | Epilepsy | 2 |  | 2 | 1 |  | 1 |  | 1 |  | 7 | N/A |
| Bishop (2020) | Stroke and traumatic brain injury | 2 |  | 2 | 1 |  | 0 |  | 1 |  | 6 | N/A |
| Cho et al. (2020) | Congenital heart defects (unspecified) | 2 |  | 2 | 1 |  | 0 |  | 1 |  | 6 | N/A |
| Cho et al. (2020) | Congenital heart defects | 2 | 0 | 2 | 1 |  | 0 | 1 | 1 | 1 | 6 | 8 |
| Cuypers et al. (2021) | All outcomes | 1 | 1 | 2 | 1 |  | 1 | 1 | 1 | 0 | 6 | 7 |
| Garcia et al. (2020) | All outcomes | 1 | 0 | 0 | 0 |  | 1 | 1 | 0 | 0 | 2 | 2 |
| Kristianslund et al. (2021) | All outcomes | 2 |  | 2 | 1 |  | 0 |  | 1 |  | 6 | N/A |
| McMahon et al. (2021) | All outcomes | 1 | 1 | 1 | 0 |  | 1 | 1 | 1 | 1 | 4 | 6 |
| Monaghan et al. (2021) | All outcomes | 2 |  | 0 | 0 |  | 2 |  | 0 |  | 4 | N/A |
| O'Brien et al. (2020) | Hypertension and obesity | 2 |  | 0 | 0 |  | 1 |  | 1 |  | 4 | N/A |
| O'Brien et al. (2020) | Diabetes | 2 |  | 0 | 0 |  | 1 |  | 0 |  | 3 | N/A |
| Ostermaier et al. (2020) | Celiac disease | 2 | 0 | 1 | 1 | 1 | 0 |  | 1 | 0 | 6 | 6 |
| Tyrer et al. (2020) | Diabetes | 2 |  | 1 | 0 |  | 1 |  | 0 |  | 4 | N/A |
| Xie et al. (2020) | Asthma (active) | 2 | 1 | 0 | 0 |  | 1 | 2 | 0 | 1 | 3 | 6 |

Response rates/follow-up: includes two items, details see Table TS1/S2.

No outcome at the start: only applied to the longitudinal analysis.

* Only applied to studies comparing between people with and without intellectual disability.

^ Sum 1 refers to the NOS score for studies that estimated prevalence/incidence; Sum 2 refers to the NOS score for studies that estimated comparative results (e.g., relative risk).
