# Supplementary material for: Prevalence and incidence of physical health conditions in people with intellectual disability – a systematic review
Source: PLoS One. 2021 Aug 24;16(8):e0256294. doi: 10.1371/journal.pone.0256294 (PMC8384165; doi:10.1371/journal.pone.0256294)
Supplement: S4 File — (DOCX) [file pone.0256294.s004.docx]

**Results tables, including prevalence, incidence, and risk estimates**

## **Table S9. Estimates of prevalence of physical disorders in people with ID of any aetiology**

| Disorders (ICD 10 chapters) | Author (year) | Genetic syndromes | ID levels | ID age range | Outcome ascertainment | Prevalence type | ID prevalence estimate |
| --- | --- | --- | --- | --- | --- | --- | --- |
| Certain infectious and parasitic diseases (A00-B99) | | | |  |  |  |  |
| Viral hepatitis | Cooper et al. (2015)[1] | Not specified | NR | ≥18 | Medical record/ registry | Point | 0.09% |
| HIV | Lunsky et al. (2017)[2] | Not specified | NR | 18-64 | Medical record/ registry | Period | 0.17% * |
| Neoplasms (C00-D48) | |  |  |  |  |  |  |
| All cancers | Cooper et al. (2018)[3] | Not specified | All | 18-92 | Medical record/ registry | Period | 0.97% |
| All cancers | Carey et al. (2016)[4] | Not specified | NR | 18-84 | Medical record/ registry | Period | 1.61% |
| Leukaemia | Fabia et al. (1970)[5] | Down syndrome | NR | Young children | Medical record/ registry | Period | 1.06% |
| Leukaemia | Leonard et al. (1999)[6] | Down syndrome | NR | 5-17 | Self or informant-reported | Lifetime | 1.42% * |
| Leukaemia | McGrother et al. (1990)[7] | Down syndrome | NR | 1-10 | Medical record/ registry | Lifetime | 1.87% * |
| Acute myelogenous leukemia (early presenting) | Arnell et al. (2012)[8] | Down syndrome | NR | Infants | Medical record/ registry | Lifetime | 0.97% |
| Haemangioma/ lymphangioma | McGrother et al. (1990)[7] | Down syndrome | NR | 1-10 | Medical record/ registry | Lifetime | 0.93% * |
| Endocrine, nutritional and metabolic diseases (E00-E90) | | | |  |  |  |  |
| Thyrotoxicosis/  thyroid disorders | Cooper et al. (2015)[1] | Not specified | NR | ≥18 | Medical record/ registry | Point | 7.85% |
| Thyroid disorders | Beange et al. (1995)[9] | Not specified | All | 20-50 | Clinical examination | Point | 12.00% |
| Hypothyroidism | Cooper et al. (2018)[3] | Not specified | All | 18-92 | Medical record/ registry | Period | 5.27% |
| Thyroid disorders | Kapell et al. (1998)[10] | Down syndrome | All | ≥45 | Self or informant-reported | Age-adjusted point | 38.10% |
| Hypothyroidism | Määttä et al. (2011)[11] | Down syndrome | All | 0.6-59.2 | Medical record/ registry | Lifetime | 26.00% |
| Hypothyroidism  (early presenting) | Arnell et al. (2012)[8] | Down syndrome | NR | Infants | Medical record/ registry | Lifetime | 0.97% |
| Hypothyroidism  (congenital) | Jaruratanasirikul et al. (2017)[12] | Down syndrome | NR | Infants | Medical record/ registry | Period | 12.08% |
| Hypothyroidism | Leonard et al. (1999)[6] | Down syndrome | NR | 5-17 | Self or informant-reported | Lifetime | 9.00% * |
| Hypothyroidism | Prasher et al. (2014)[13] | Down syndrome | NR | 18-43 | Self or informant-reported | Point | 39.00% |
| Hypothyroidism | Henderson et al. (2007)[14] | Down syndrome | NR | 18-61 | Medical record/ registry | Point | 23.44% |
| Hypothyroidism | Diene et al. (2010)[15] | Prader-Willi syndrome | NR | ≤18 | Medical record/ registry | Point | 24.41% |
| Hyperthyroidism | Leonard et al. (1999)[6] | Down syndrome | NR | 5-17 | Self or informant-reported | Lifetime | 2.37% * |
| Diabetes | Carey et al. (2016)[4] | Not specified | NR | 18-84 | Medical record/ registry | Period | 6.89% |
| Diabetes | Cooper et al. (2018)[3] | Not specified | All | 18-92 | Medical record/ registry | Period | 6.38% |
| Diabetes | Cooper et al. (2015)[1] | Not specified | NR | ≥18 | Medical record/ registry | Point | 6.63% |
| Diabetes | Cuypers et al. (2021)[16] | Not specified | NR | All ages | Medical record/ registry | Period | 9.90% |
| Diabetes | Leonard et al. (1999)[6] | Down syndrome | NR | 5-17 | Self or informant-reported | Lifetime | 0.95% * |
| Diabetes | Prasher et al. (2014)[13] | Down syndrome | NR | 18-43 | Self or informant-reported | Point | 4.00% |
| Diabetes | Kapell et al. (1998)[10] | Down syndrome | All | ≥45 | Medical record/ registry | Age-adjusted point | 4.90% |
| Type 1 diabetes | Henderson et al. (2007)[14] | Down syndrome | NR | 18-61 | Medical record/ registry | Point | 4.69% |
| Type 2 diabetes | Diene et al. (2010)[15] | Prader-Willi syndrome | NR | ≤18 | Medical record/ registry | Point | 0.00% |
| Hypogonadism | Diene et al. (2010)[15] | Prader-Willi syndrome | NR | ≤18 | Medical record/ registry | Point | 93.10% |
| Obesity | Mikulovic et al. (2011)[17] | Not specified | NR | 11-20 | Clinical examination | Point | 3.90% |
| Obesity | Simila et al. (1991)[18] | Not specified | All | 20 | Medical record/ registry | Point | 9.80% |
| Obesity | Boyle et al. (2010)[19] | Not specified | All | ≥16 | Medical record/ registry/clinical examination | Point | 33.00% |
| Obesity | Gale et al. (2009)[20] | Not specified | NR | ≥16 | Medical record/ registry | Period | 33.14% |
| Obesity | Melville et al. (2008)[21] | Not specified | All | ≥16 | Clinical examination | Point | 31.53% * |
| Obesity (mild/moderate) | Melville et al. (2008) | Not specified | All | ≥16 | Clinical examination | Point | 26.67% |
| Obesity (severe/morbid) | Melville et al. (2008) | Not specified | All | ≥16 | Clinical examination | Point | 6.24% |
| Obesity | Hove et al. (2004)[22] | Not specified | All | ≥18 | Clinical examination/ medical record/ registry | Point | 19.10% |
| Obesity | Beange et al. (1995)[9] | Not specified | All | 20-50 | Clinical examination | Point | Male: 7.7%; Female: 8.5% |
| Obesity | Garg et al. (2018)[23] | Down syndrome | NR | ≥18 | Medical record/ registry | Period | 59.53% |
| Obesity | Melville et al. (2005)[24] | Down syndrome | NR | 20-69 | Clinical examination | Period | 25.51% * |
| Overweight | Gale et al. (2009)[20] | Not specified | NR | ≥16 | Medical record/ registry | Period | 26.89% |
| Overweight | Hove et al. (2004)[22] | Not specified | All | ≥18 | Clinical examination/ medical record/ registry | Point | 34.80% |
| Overweight | Melville et al. (2008)[21] | Not specified | All | ≥16 | Clinical examination | Point | 31.53% * |
| Overweight | Simila et al. (1991)[18] | Not specified | All | 20 | Medical record/ registry | Point | 33.20% |
| Overweight | Melville et al. (2005)[24] | Down syndrome | NR | 20-69 | Clinical examination | Period | 37.65% * |
| Overweight | Prasher et al. (2014)[13] | Down syndrome | NR | 18-43 | Self or informant-reported | Point | 49.00% |
| Overweight/obese | Mikulovic et al. (2011)[17] | Not specified | NR | 11-20 | Clinical examination | Point | 19.00% |
| Diseases of the respiratory system (J00-J99) | | |  |  |  |  |  |
| Tonsillitis | Leonard et al. (1999)[6] | Down syndrome | NR | 5-17 | Self or informant-reported | Period | 15.64% * |
| Cold or influenza | Leonard et al. (1999)[6] | Down syndrome | NR | 5-17 | Self or informant-reported | Period | 78.67% * |
| Pneumonia | Määttä et al. (2011)[11] | Down syndrome | All | 0.6-59.2 | Medical record/ registry | Lifetime | 27.00% |
| Pneumonia | Leonard et al. (1999)[6] | Down syndrome | NR | 5-17 | Self or informant-reported | Period | 11.37% * |
| Bronchitis | Leonard et al. (1999)[6] | Down syndrome | NR | 5-17 | Self or informant-reported | Period | 11.37% * |
| Chronic sinusitis | Cooper et al. (2015)[1] | Not specified | NR | ≥18 | Medical record/ registry | Point | 0.30% |
| Vocal cord paralysis | Hamilton et al. (2016)[25] | Down syndrome | NR | 0-20 | Medical record/ registry | Period | 0.42% |
| Tracheobronchomalacia | Hamilton et al. (2016)[25] | Down syndrome | NR | 0-20 | Medical record/ registry | Period | 7.11% |
| Chronic obstructive pulmonary disease | Carey et al. (2016)[4] | Not specified | NR | 18-84 | Medical record/ registry | Period | 1.08% |
| Chronic obstructive pulmonary disease | Cooper et al. (2018)[3] | Not specified | All | 18-92 | Medical record/ registry | Period | 1.25% |
| Chronic obstructive pulmonary disease | Cooper et al. (2015)[1] | Not specified | NR | ≥18 | Medical record/ registry | Point | 2.61% |
| Asthma | Carey et al. (2016)[4] | Not specified | NR | 18-84 | Medical record/ registry | Period | 8.19% |
| Asthma | Cooper et al. (2018)[3] | Not specified | All | 18-92 | Medical record/ registry | Period | 9.15% |
| Asthma | Beange et al. (1995)[9] | Not specified | All | 20-50 | Clinical examination | Point | 2.50% |
| Asthma | Gale et al. (2009) | Not specified | NR | ≥16 | Medical record/registry | Period | 12.03% |
| Asthma  (active) | Cooper et al. (2015)[1] | Not specified | NR | ≥18 | Medical record/ registry | Point | 7.17% |
| Asthma | Määttä et al. (2011)[11] | Down syndrome | All | 0.6-59.2 | Medical record/ registry | Lifetime | 2.00% |
| Asthma | Leonard et al. (1999)[6] | Down syndrome | NR | 5-17 | Self or informant-reported | Period | 8.53% * |
| Bronchiectasis | Cooper et al. (2015)[1] | Not specified | NR | ≥18 | Medical record/ registry | Point | 0.25% |
| Acquired subglottic stenosis | Hamilton et al. (2016)[25] | Down syndrome | NR | 0-20 | Medical record/ registry | Period | 4.60% |
| Diseases of the nervous system (G00-G99) | | |  |  |  |  |  |
| Parkinson’s disease and Parkinsonism | Cooper et al. (2015)[1] | Not specified | NR | ≥18 | Medical record/ registry | Point | 0.35% |
| Multiple sclerosis | Cooper et al. (2015)[1] | Not specified | NR | ≥18 | Medical record/ registry | Point | 0.11% |
| Epilepsy  (active) | Forsgren et al., (1990)[26] | Not specified | All | 0-79 | Self or informant-reported | Period | 20.22% |
| Epilepsy | Arvio et al. (2003)[27] | Not specified | Severe - profound | 1-72 | Medical record/ registry | Lifetime | 51.84% |
| Epilepsy | Benassi et al. (1990)[28] | Not specified | Severe - profound | 6-13 | Medical record/ registry | Lifetime | 30.00% |
| Epilepsy | Gustavson et al. (1977)[29] | Not specified | Severe - profound | 5-16 | Medical record/ registry | Lifetime | 29.51% |
| Epilepsy | McQueen et al. (1987)[30] | Not specified | Moderate - profound | 7-10 | Medical record/ registry/non-health administrative registry | Lifetime | 23.10% |
| Epilepsy | Wellesley et al. (1992)[31] | Not specified | All | 6-16 | Medical record/ registry | Lifetime | 13.00% |
| Epilepsy | Christianson et al. (2002)[32] | Not specified | Severe - profound | 2-9 | Clinical examination | Point | 25.58% |
| Epilepsy | Shepherd et al. (1989)[33] | Not specified | Mild - severe | 5-16 | Medical record/ registry | Point | 18.00% |
| Epilepsy | Matthews et al. (2008)[34] | Not specified | NR | 17-86 | Medical record/ registry | Lifetime | 18.24% |
| Epilepsy | Carey et al. (2016)[4] | Not specified | NR | 18-84 | Medical record/ registry | Period | 18.51% |
| Epilepsy | Cooper et al. (2018)[3] | Not specified | All | 18-92 | Medical record/ registry | Period | 28.16% |
| Epilepsy | Finlayson et al. (2010)[35] | Not specified | All | 16-79 | Medical record/ registry | Period | 32.40% |
| Epilepsy | Morgan et al. (2003)[36] | Not specified | NR | ≥15 | Medical record/ registry | Period | 16.10% |
| Epilepsy | Boyle et al. (2010)[19] | Not specified | All | ≥16 | Medical record/ registry/clinical examination | Point | 34.10% |
| Epilepsy | Cooper et al. (2015)[1] | Not specified | NR | ≥18 | Medical record/ registry | Point | 18.82% |
| Epilepsy | Janicki et al. (1984)[37] | Not specified | Mild - moderate | 73-99 | Self or informant-reported | Point | 13.00% |
| Epilepsy | Janicki et al. (1984)[37] | Not specified | Mild - moderate | 63-72 | Self or informant-reported | Point | 14.00% |
| Epilepsy | Janicki et al. (1984)[37] | Not specified | Mild - moderate | 53-62 | Self or informant-reported | Point | 14.00% |
| Epilepsy | Janicki et al. (1984)[37] | Not specified | Severe - profound | 73-99 | Self or informant-reported | Point | 9.00% |
| Epilepsy | Janicki et al. (1984)[37] | Not specified | Severe - profound | 63-72 | Self or informant-reported | Point | 14.00% |
| Epilepsy | Janicki et al. (1984)[37] | Not specified | Severe - profound | 53-62 | Self or informant-reported | Point | 19.00% |
| Epilepsy | McGrother et al. (2006)[38] | Not specified | NR | ≥20 | Self or informant-reported | Point | 25.91% |
| Epilepsy | Melville et al. (2008)[21] | Not specified | All | ≥16 | Clinical examination | Point | 32.80% * |
| Epilepsy | Bishop et al. (2020)[39] | Not specified | NR | ≥21 | Medical record/registry | Period | 27.94%* |
| Epilepsy | Beange et al. (1995)[9] | Not specified | All | 20-50 | Clinical examination | Point | 29.00% |
| Epilepsy | Määttä et al. (2011)[11] | Down syndrome | All | 0.6-59.2 | Medical record/ registry | Lifetime | 7.00% |
| Epilepsy | McGrother et al. (1990)[7] | Down syndrome | NR | 1-10 | Medical record/ registry | Lifetime | 0.93% * |
| Epilepsy | Johannsen et al. (1996)[40] | Down syndrome | NR | 14-60 | Self or informant-reported | Point | 16.67% |
| Migraine | Cooper et al. (2015)[1] | Not specified | NR | ≥18 | Medical record/ registry | Point | 0.74% |
| Cerebral palsy | Benassi et al. (1990)[28] | Not specified | Severe - profound | 6-13 | Medical record/ registry | Lifetime | 28.89% |
| Cerebral palsy | Gustavson et al. (1977)[29] | Not specified | Severe - profound | 5-16 | Medical record/ registry | Lifetime | 18.03% |
| Cerebral palsy | McQueen et al. (1987)[30] | Not specified | Moderate - profound | 7-10 | Medical record/ registry/non-health administrative registry | Lifetime | 15.40% |
| Cerebral palsy | Wellesley et al. (1992)[31] | Not specified | All | 6-16 | Medical record/ registry | Lifetime | 19.80% |
| Cerebral palsy | Christianson et al. (2002)[32] | Not specified | Severe - profound | 2-9 | Clinical examination | Point | 25.58% |
| Cerebral palsy | Janicki et al. (1984)[37] | Not specified | Mild - moderate | 73-99 | Self or informant-reported | Point | 1.00% |
| Cerebral palsy | Janicki et al. (1984)[37] | Not specified | Mild - moderate | 63-72 | Self or informant-reported | Point | 3.00% |
| Cerebral palsy | Janicki et al. (1984)[37] | Not specified | Mild - moderate | 53-62 | Self or informant-reported | Point | 6.00% |
| Cerebral palsy | Janicki et al. (1984)[37] | Not specified | Severe - profound | 73-99 | Self or informant-reported | Point | 4.00% |
| Cerebral palsy | Janicki et al. (1984)[37] | Not specified | Severe - profound | 63-72 | Self or informant-reported | Point | 4.00% |
| Cerebral palsy | Janicki et al. (1984)[37] | Not specified | Severe - profound | 53-62 | Self or informant-reported | Point | 6.00% |
| Dystonic tetraplegia | Arvio et al. (2003)[27] | Not specified | Severe - profound | 1-72 | Medical record/ registry | Lifetime | 8.46% |
| Spastic tetraplegia | Arvio et al. (2003)[27] | Not specified | Severe - profound | 1-72 | Medical record/ registry | Lifetime | 14.32% |
| Spastic diplegia | Arvio et al. (2003)[27] | Not specified | Severe - profound | 1-72 | Medical record/ registry | Lifetime | 9.33% |
| Spastic hemiplegia | Arvio et al. (2003)[27] | Not specified | Severe - profound | 1-72 | Medical record/ registry | Lifetime | 1.52% |
| Paraplegia | Arvio et al. (2003)[27] | Not specified | Severe - profound | 1-72 | Medical record/ registry | Lifetime | 1.08% |
| Hydrocephalus | Gustavson et al. (1977)[29] | Not specified | Severe - profound | 5-16 | Medical record/ registry | Lifetime | 5.74% * |
| Hydrocephaly | Christianson et al. (2002)[32] | Not specified | Severe - profound | 2-9 | Clinical examination | Point | 4.65% |
| Diseases of the eye and adnexa (H00-H59) | | |  |  |  |  |  |
| Corneal guttata | Henderson et al. (2007)[14] | Down syndrome | NR | 18-61 | Medical record/ registry | Point | 1.56% |
| Keratoconus | Henderson et al. (2007)[14] | Down syndrome | NR | 18-61 | Medical record/ registry | Point | 1.56% * |
| Keratoconus | Kristianslund et al. (2021)[41] | Down syndrome | NR | NR | Medical record/registry | Period | 5.50% |
| Retinitis pigmentosa | Henderson et al. (2007)[14] | Down syndrome | NR | 18-61 | Medical record/ registry | Point | 3.13% * |
| Retinopathy of prematurity | Atladottir et al. (2015)[42] | Not specified | NR | 0-16 | Medical record/ registry | Point | 15.60% |
| Glaucoma | Cooper et al. (2015)[1] | Not specified | NR | ≥18 | Medical record/ registry | Point | 0.90% |
| Glaucoma | Henderson et al. (2007)[14] | Down syndrome | NR | 18-61 | Medical record/ registry | Point | 3.13% * |
| Strabismus | Henderson et al. (2007)[14] | Down syndrome | NR | 18-61 | Medical record/ registry | Point | 6.25% * |
| Strabismus | McGrother et al. (1990)[7] | Down syndrome | NR | 1-10 | Medical record/ registry | Lifetime | 1.87% * |
| Astigmatism | Leonard et al. (1999)[6] | Down syndrome | NR | 5-17 | Self or informant-reported | Lifetime | 9.10% |
| Myopia | Leonard et al. (1999)[6] | Down syndrome | NR | 5-17 | Self or informant-reported | Lifetime | 29.70% |
| Hyperopia | Leonard et al. (1999)[6] | Down syndrome | NR | 5-17 | Self or informant-reported | Lifetime | 14.40% |
| Visual impairment | Boyle et al. (2010)[19] | Not specified | All | ≥16 | Medical record/ registry/clinical examination | Point | 47.00% |
| Visual impairment | Cooper et al. (2015)[1] | Not specified | NR | ≥18 | Medical record/ registry | Point | 3.22% |
| Visual impairment | Melville et al. (2008)[21] | Not specified | All | ≥16 | Clinical examination | Point | 46.88% * |
| Visual impairment | Finlayson et al. (2010)[35] | Not specified | All | 16-79 | Medical record/ registry | Period | 44.42% |
| Visual impairment | Wellesley et al. (1992)[31] | Not specified | All | 6-16 | Medical record/ registry | Lifetime | 30.80% * |
| Visual impairment | Kapell et al. (1998)[10] | Down syndrome | All | ≥45 | Self or informant-reported | Age-adjusted point | 34.90% |
| Blindness | Arvio et al. (2003)[27] | Not specified | Severe - profound | 1-72 | Medical record/ registry | Lifetime | 8.03% |
| Blindness | Wellesley et al. (1992)[31] | Not specified | All | 6-16 | Medical record/ registry | Lifetime | 4.99% |
| Blindness | Beange et al. (1995)[9] | Not specified | All | 20-50 | Clinical examination | Point | 4.40% |
| Blindness | Prasher et al. (2014)[13] | Down syndrome | NR | 18-43 | Self or informant-reported | Point | 0.80% * |
| Blindness | Henderson et al. (2007)[14] | Down syndrome | NR | 18-61 | Medical record/ registry | Point | 3.13% * |
| Nystagmus | Leonard et al. (1999)[6] | Down syndrome | NR | 5-17 | Self or informant-reported | Lifetime | 2.90% |
| Nystagmus | McGrother et al. (1990)[7] | Down syndrome | NR | 1-10 | Medical record/ registry | Lifetime | 0.93% * |
| Nystagmus | Henderson et al. (2007)[14] | Down syndrome | NR | 18-61 | Medical record/ registry | Point | 4.69% * |
| Diseases of the ear and mastoid process (H60-H95) | | | |  |  |  |  |
| Otitis media with effusion | Austeng et al. (2013)[43] | Down syndrome | NR | 8 | Clinical examination | Point | 38.00% |
| Otitis media with effusion | Barr et al. (2011)[44] | Down syndrome | NR | <1 | Medical record/ registry | Point | 83.00% |
| Otitis media with effusion | Barr et al. (2011)[44] | Down syndrome | NR | 1 | Medical record/ registry | Point | 93.00% |
| Otitis media with effusion | Barr et al. (2011)[44] | Down syndrome | NR | 2 | Medical record/ registry | Point | 90.00% |
| Otitis media with effusion | Barr et al. (2011)[44] | Down syndrome | NR | 3 | Medical record/ registry | Point | 76.00% |
| Otitis media with effusion | Barr et al. (2011)[44] | Down syndrome | NR | 4 | Medical record/ registry | Point | 77.00% |
| Otitis media with effusion | Yaneza et al. (2016)[45] | Down syndrome | NR | 5 | Medical record/ registry | Point | 48.10% |
| Otitis media with effusion | Yaneza et al. (2016)[45] | Down syndrome | NR | 6 | Medical record/ registry | Point | 35.30% |
| Otitis media with effusion | Yaneza et al. (2016)[45] | Down syndrome | NR | 7 | Medical record/ registry | Point | 51.70% |
| Otitis media with effusion | Yaneza et al. (2016)[45] | Down syndrome | NR | 8 | Medical record/ registry | Point | 44.40% |
| Otitis media with effusion | Yaneza et al. (2016)[45] | Down syndrome | NR | 9 | Medical record/ registry | Point | 30.00% |
| Otitis media with effusion | Yaneza et al. (2016)[45] | Down syndrome | NR | 10 | Medical record/ registry | Point | 44.10% |
| Otitis media with effusion | Yaneza et al. (2016)[45] | Down syndrome | NR | 11 | Medical record/ registry | Point | 45.00% |
| Otitis media with effusion | Yaneza et al. (2016)[45] | Down syndrome | NR | 12 | Medical record/ registry | Point | 38.50% |
| Middle ear infection | Määttä et al. (2011)[11] | Down syndrome | All | 0.6-59.2 | Medical record/ registry | Lifetime | 44.00% |
| Ear infection | Leonard et al. (1999)[6] | Down syndrome | NR | 5-17 | Self or informant-reported | Period | 30.81% * |
| Perforated ear drum | Leonard et al. (1999)[6] | Down syndrome | NR | 5-17 | Self or informant-reported | Lifetime | 6.20% |
| Hearing loss | Cooper et al. (2015)[1] | Not specified | NR | ≥18 | Medical record/ registry | Point | 8.20% |
| Hearing loss | Meuwese-Jongejeugd et al. (2006)[46] | Not specified | All | 20.19-88.73 | Medical record/ registry/clinical examination | Period | 34.90% |
| Hearing loss | Meuwese-Jongejeugd et al. (2006)[46] | Not specified | All | 20.19-88.73 | Medical record/ registry/clinical examination | Weighted | 30.30% |
| Hearing loss | Wellesley et al. (1992)[31] | Not specified | All | 6-16 | Medical record/ registry | Lifetime | 18.00% * |
| Hearing loss | Määttä et al. (2011)[11] | Down syndrome | All | 0.6-59.2 | Medical record/ registry | Lifetime | 27.00% |
| Hearing loss | Austeng et al. (2013)[47] | Down syndrome | NR | 8 | Clinical examination | Point | 35.00% |
| Hearing loss | Leonard et al. (1999)[6] | Down syndrome | NR | 5-17 | Self or informant-reported | Lifetime | 10.50% |
| Hearing loss | Park et al. (2012)[48] | Down syndrome | NR | Infants | Medical record/ registry | Period | 46.10% |
| Hearing loss | Meuwese-Jongejeugd et al. (2006)[46] | Down syndrome | All | 20.85-75.93 | Medical record/ registry/clinical examination | Weighted | 57.40% |
| Hearing loss (congenital) | Tedeschi et al. (2015)[49] | Down syndrome | NR | Infants | Medical record/ registry | Point | 15.00% |
| Conductive hearing loss | Austeng et al. (2013)[47] | Down syndrome | NR | 8 | Clinical examination | Point | 16.00% |
| Conductive hearing loss | Park et al. (2012)[48] | Down syndrome | NR | Infants | Medical record/ registry | Period | 40.60% |
| Conductive or mixed hearing loss | Austeng et al. (2013)[47] | Down syndrome | NR | 8 | Clinical examination | Point | 6.10% |
| Sensorineural hearing loss | Park et al. (2012)[48] | Down syndrome | NR | Infants | Medical record/ registry | Period | 1.86% |
| Mixed hearing loss | Park et al. (2012)[48] | Down syndrome | NR | Infants | Medical record/ registry | Period | 0.93% |
| Mixed hearing loss | Yaneza et al. (2016)[45] | Down syndrome | NR | 5-12 | Medical record/ registry | Period | 6.86% |
| Indeterminant hearing loss | Park et al. (2012)[48] | Down syndrome | NR | Infants | Medical record/ registry | Period | 2.80% |
| Deafness | Arvio et al. (2003)[27] | Not specified | Severe - profound | 1-72 | Medical record/ registry | Lifetime | 1.52% |
| Deafness | Beange et al. (1995)[9] | Not specified | All | 20-50 | Clinical examination | Point | 25.00% |
| Deafness | Wellesley et al. (1992)[31] | Not specified | All | 6-16 | Medical record/ registry | Lifetime | 1.37% * |
| Deafness | McGrother et al. (1990)[7] | Down syndrome | NR | 1-10 | Medical record/ registry | Lifetime | 0.93% * |
| Diseases of the circulatory system (I00-I99) | | |  |  |  |  |  |
| Hypertension | Carey et al. (2016)[4] | Not specified | NR | 18-84 | Medical record/ registry | Period | 10.73% |
| Hypertension | Cooper et al. (2018)[3] | Not specified | All | 18-92 | Medical record/ registry | Period | 12.76% |
| Hypertension | Cooper et al. (2015)[1] | Not specified | NR | ≥18 | Medical record/ registry | Point | 9.66% |
| Hypertension | Beange et al. (1995)[9] | Not specified | All | 20-50 | Clinical examination | Point | Male: 11.5%; Female: 8.6% |
| Hypertension | Kapell et al. (1998)[10] | Down syndrome | All | ≥45 | Self or informant-reported | Age-adjusted point | 2.60% |
| Hypertension | Prasher et al. (2014)[13] | Down syndrome | NR | 18-43 | Self or informant-reported | Point | 1.60% * |
| Coronary heart disease | Cooper et al. (2015)[1] | Not specified | NR | ≥18 | Medical record/ registry | Point | 2.00% |
| Coronary heart disease | Cooper et al. (2018)[3] | Not specified | All | 18-92 | Medical record/ registry | Period | 3.47% |
| Ischemic heart disease | Carey et al. (2016)[4] | Not specified | NR | 18-84 | Medical record/ registry | Period | 1.65% |
| Ischemic heart disease | Kapell et al. (1998)[10] | Down syndrome | All | ≥45 | Self or informant-reported | Age-adjusted point | 4.40% |
| Atrial fibrillation | Carey et al. (2016)[4] | Not specified | NR | 18-84 | Medical record/ registry | Period | 0.83% |
| Atrial fibrillation | Cooper et al. (2018)[3] | Not specified | All | 18-92 | Medical record/ registry | Period | 0.97% |
| Atrial fibrillation | Cooper et al. (2015)[1] | Not specified | NR | ≥18 | Medical record/ registry | Point | 0.89% |
| Heart failure | Carey et al. (2016)[4] | Not specified | NR | 18-84 | Medical record/ registry | Period | 0.82% |
| Heart failure | Cooper et al. (2018)[3] | Not specified | All | 18-92 | Medical record/ registry | Period | 2.50% |
| Heart failure | Cooper et al. (2015)[1] | Not specified | NR | ≥18 | Medical record/ registry | Point | 1.02% |
| Stroke | Cooper et al. (2018)[3] | Not specified | All | 18-92 | Medical record/ registry | Period | 1.80% |
| Stroke | Bishop et al. (2020)[39] | Not specified | All | ≥21 | Medical record/ registry | Period | 4.65%* |
| Stroke | Määttä et al. (2011)[11] | Down syndrome | All | 0.6-59.2 | Medical record/ registry | Lifetime | 2.00% |
| Stroke | Kapell et al. (1998)[10] | Down syndrome | All | ≥45 | Self or informant-reported | Age-adjusted point | 3.80% |
| Peripheral vascular disease | Carey et al. (2016)[4] | Not specified | NR | 18-84 | Medical record/ registry | Period | 0.41% |
| Peripheral vascular disease | Cooper et al. (2015)[1] | Not specified | NR | ≥18 | Medical record/ registry | Point | 0.57% |
| Diseases of the digestive system (K00-K93) | | |  |  |  |  |  |
| Inflammatory bowel disease | Cooper et al. (2015)[1] | Not specified | NR | ≥18 | Medical record/ registry | Point | 0.50% |
| Ischaemic colitis | McGrother et al. (1990)[7] | Down syndrome | NR | 1-10 | Medical record/ registry | Lifetime | 1.87% * |
| Diverticular disease | Cooper et al. (2015)[1] | Not specified | NR | ≥18 | Medical record/ registry | Point | 0.82% |
| Irritable bowel syndrome | Cooper et al. (2015)[1] | Not specified | NR | ≥18 | Medical record/ registry | Point | 3.09% |
| Constipation | Cooper et al. (2015)[1] | Not specified | NR | ≥18 | Medical record/ registry | Point | 13.95% |
| Constipation | Leonard et al. (1999)[6] | Down syndrome | NR | 5-17 | Self or informant-reported | Lifetime | 18.40% |
| Cholestasis (neonatal) | Arnell et al. (2012)[8] | Down syndrome | NR | Infants | Medical record/ registry | Lifetime | 3.90% |
| Celiac disease | Henderson et al. (2007)[14] | Down syndrome | NR | 18-61 | Medical record/ registry | Point | 10.94% |
| Celiac disease | Jansson et al. (1995)[50] | Down syndrome | NR | <18 | Clinical examination/ medical record/ registry (n=2) | Point | 16.90% |
| Celiac disease | Leonard et al. (1999)[6] | Down syndrome | NR | 5-17 | Self or informant-reported | Lifetime | 0.10% |
| Intestinal obstruction site not specified | Fabia et al. (1970) [5] | Down syndrome | NR | Young children | Medical record/ registry | Period | 0.37% * |
| Diseases of the skin and subcutaneous tissue (L00-L99) | | | |  |  |  |  |
| Dermatitis | Prasher et al. (2014)[13] | Down syndrome | NR | 18-43 | Self or informant-reported | Point | 14.00% |
| Eczema | Henderson et al. (2007)[14] | Down syndrome | NR | 18-61 | Medical record/ registry | Point | 23.44% |
| Psoriasis | Prasher et al. (2014)[13] | Down syndrome | NR | 18-43 | Self or informant-reported | Point | 13.00% |
| Hidradenitis suppurativa | Garg et al. (2018)[23] | Down syndrome | NR | ≥18 | Medical record/ registry | Period | 2.14% |
| Diseases of the musculoskeletal system and connective tissue (M00-M99) | | | |  |  |  |  |
| Arthritis (Inflammatory) | Cooper et al. (2015)[1] | Not specified | NR | ≥18 | Medical record/ registry | Point | 1.88% |
| Arthritis | Leonard et al. (1999)[6] | Down syndrome | NR | 5-17 | Self or informant-reported | Lifetime | 0.95% * |
| Rheumatoid arthritis | Carey et al. (2016)[4] | Not specified | NR | 18-84 | Medical record/ registry | Period | 0.49% |
| Osteoarthritis | Henderson et al. (2007)[14] | Down syndrome | NR | 18-61 | Medical record/ registry | Point | 14.06% |
| Scoliosis | Thomson et al. (2006)[51] | Angelman syndrome | All | 6.5-39.0 | Medical record/ registry | Lifetime | 33.00% |
| Osteoporosis | Carey et al. (2016)[4] | Not specified | NR | 18-84 | Medical record/ registry | Period | 1.67% |
| Osteoporosis | Burke et al. (2019)[52] | Not specified | All | ≥43 | Clinical examination | Period | 41.00% * |
| Perthe's disease | Leonard et al. (1999)[6] | Down syndrome | NR | 5-17 | Self or informant-reported | Lifetime | 0.95% * |
| Diseases of the genitourinary system (N00-N99) | | |  |  |  |  |  |
| Chronic kidney disease | Carey et al. (2016)[4] | Not specified | NR | 18-84 | Medical record/ registry | Period | 3.17% |
| Chronic kidney disease | Cooper et al. (2018)[3] | Not specified | All | 18-92 | Medical record/ registry | Period | 2.08% |
| Chronic kidney disease | Cooper et al. (2015)[1] | Not specified | NR | ≥18 | Medical record/ registry | Point | 1.68% |
| Prostate disease | Cooper et al. (2015)[1] | Not specified | NR | ≥18 | Medical record/ registry | Point | 0.51% |
| Certain conditions originating in the perinatal period (P00-P96) | | | |  |  |  |  |
| Persistent pulmonary hypertension of the neonate | Weijerman et al. (2010)[53] | Down syndrome | NR | Infants | Medical record/ registry | Point | 5.20% |
| Persistent pulmonary hypertension of the newborn | Atladottir et al. (2015)[42] | Not specified | NR | 0-16 | Medical record/ registry | Point | 0.49% |
| Neonatal sepsis | Atladottir et al. (2015)[42] | Not specified | NR | 0-16 | Medical record/ registry | Point | 3.88% |
| Neonatal hypoglycemia | Atladottir et al. (2015)[42] | Not specified | NR | 0-16 | Medical record/ registry | Point | 6.46% |
| Necrotizing enterocolitis | Arnell et al. (2012)[8] | Down syndrome | NR | Infants | Medical record/ registry | Lifetime | 0.97% |
| Congenital malformations, deformations and chromosomal abnormalities (Q00-Q99) | | | | |  |  |  |
| Anencephalus and spina bifida | Torfs et al. (1998)[54] | Down syndrome | NR | <1 | Medical record/ registry | Point | 0.00% |
| Encephalocele | Torfs et al. (1998)[54] | Down syndrome | NR | <1 | Medical record/ registry | Point | 0.04% |
| Microcephaly | Christianson et al. (2002)[32] | Not specified | Severe - profound | 2-9 | Clinical examination | Point | 20.93% |
| Congenital hydrocephalus | Torfs et al. (1998)[54] | Down syndrome | NR | <1 | Medical record/ registry | Point | 0.76% |
| Hydrocephalus | Fabia et al. (1970)[5] | Down syndrome | NR | Young children | Medical record/ registry | Period | 0.17% * |
| Holoprosencephaly | Torfs et al. (1998)[54] | Down syndrome | NR | <1 | Medical record/ registry | Point | 0.10% |
| Spina bifida | Christianson et al. (2002)[32] | Not specified | Severe - profound | 2-9 | Clinical examination | Point | 0.00% |
| Spina bifida | Fabia et al. (1970)[5] | Down syndrome | NR | Young children | Medical record/ registry | Period | 0.29% * |
| Congenital cataract | Fabia et al. (1970)[5] | Down syndrome | NR | Young children | Medical record/ registry | Period | 0.91% * |
| Congenital cataract | Torfs et al. (1998)[54] | Down syndrome | NR | <1 | Medical record/ registry | Point | 0.93% |
| Congenital cataract | McGrother et al. (1990)[7] | Down syndrome | NR | 1-10 | Medical record/ registry | Lifetime | 1.87% * |
| Congenital glaucoma | Fabia et al. (1970)[5] | Down syndrome | NR | Young children | Medical record/ registry | Period | 0.08% * |
| Congenital malformation of cardiac chambers and connections, not specified | Santoro et al. (2018)[55] | Down syndrome | NR | Infants | Medical record/ registry | Period | 0.43% * |
| Double outlet right ventricle | Jaruratanasirikul et al. (2017)[12] | Down syndrome | NR | Infants | Medical record/ registry | Period | 2.68% |
| Double outlet right ventricle | Brodwall et al. (2018)[56] | Down syndrome | NR | 0-5 | Medical record/ registry | Period | 0.16% |
| Double outlet right ventricle | Torfs et al. (1998)[54] | Down syndrome | NR | <1 | Medical record/ registry | Point | 0.17% |
| Double outlet right ventricle | Cho et al. (2020)[57] | Down syndrome | NR | All ages | Medical record/  registry | Period | 0.63% |
| Double outlet right ventricle | Kim et al. (2014)[58] | Down syndrome | NR | ≤1 | Medical record/ registry | Period | 0.51% |
| Other congenital malformations of cardiac chambers and connection | Santoro et al. (2018)[55] | Down syndrome | NR | Infants | Medical record/ registry | Period | 0.43% * |
| Transposition of great vessels | Santoro et al. (2018)[55] | Down syndrome | NR | Infants | Medical record/ registry | Period | 0.43% * |
| Transposition of great vessels | Torfs et al. (1998)[54] | Down syndrome | NR | <1 | Medical record/ registry | Point | 0.07% |
| Transposition of great arteries | Cho et al. (2020)[57] | Down syndrome | NR | All ages | Medical record/  registry | Period | 0.05% |
| Transposition of great arteries | Kim et al. (2014)[58] | Down syndrome | NR | ≤1 | Medical record/ registry | Period | 0.25% |
| Truncus arteriosus | Torfs et al. (1998)[54] | Down syndrome | NR | <1 | Medical record/ registry | Point | 0.04% |
| Single ventricle | Kim et al. (2014)[58] | Down syndrome | NR | ≤1 | Medical record/ registry | Period | 0.25% |
| Single ventricle | Cho et al. (2020)[57] | Down syndrome | NR | All ages | Medical record/  registry | Period | 0.24% |
| Transposition of great arteries | Kim et al. (2014)[58] | Down syndrome | NR | ≤1 | Medical record/ registry | Period | 0.25% |
| Congenital heart defects | Määttä et al. (2011)[11] | Down syndrome | All | 0.6-59.2 | Medical record/ registry | Lifetime | 26.00% |
| Congenital heart defects | Henderson et al. (2007)[14] | Down syndrome | NR | 18-61 | Medical record/ registry | Point | 14.06% |
| Congenital heart defects | Irving et al. (2012)[59] | Down syndrome | NR | Infants | Medical record/ registry | Period | 41.66% |
| Congenital heart defects | Jaruratanasirikul et al. (2017)[12] | Down syndrome | NR | Infants | Medical record/ registry | Period | 42.95% |
| Congenital heart defects | Kim et al. (2014)[58] | Down syndrome | NR | ≤1 | Medical record/ registry | Period | 56.85% |
| Congenital heart defects | Brodwall et al. (2018)[56] | Down syndrome | NR | 0-5 | Medical record/ registry | Period | 57.90% |
| Congenital heart defects | Fabia et al. (1970)[5] | Down syndrome | NR | Young children | Medical record/ registry | Period | 28.54% * |
| Congenital heart defects | Santoro et al. (2018)[55] | Down syndrome | NR | Infants | Medical record/ registry | Period | 43.90% |
| Congenital heart defects | Scott et al. (2014)[60] | Down syndrome | NR | Infants | Clinical examination | Period | 79.20% |
| Congenital heart defects | So et al. (2007)[61] | Down syndrome | NR | 0-3 | Medical record/ registry | Period | 54.00% |
| Congenital heart defects | Bergström et al. (2016)[62] | Down syndrome | NR | Infants | Medical record/ registry | Period | 53.60% |
| Congenital heart defects | Torfs et al. (1998)[54] | Down syndrome | NR | <1 | Medical record/ registry | Point | 55.98% |
| Congenital heart defects | Weijerman et al. (2010)[53] | Down syndrome | NR | Infants | Medical record/ registry | Point | 43.00% |
| Congenital heart defects | Freeman et al. (1998)[63] | Down syndrome | NR | Infants | Medical record/ registry | Point | 44.05% |
| Congenital heart defects | McGrother et al. (1990)[7] | Down syndrome | NR | 1-10 | Medical record/ registry | Lifetime | 35.00% * |
| Congenital heart defects | Arnell et al. (2012)[8] | Down syndrome | NR | Infants | Medical record/ registry | Lifetime | 47.00% |
| Congenital heart defects | Cho et al. (2020)[57] | Down syndrome | NR | All ages | Medical record/ registry | Period | 50.0% |
| Atrial septal defect | Freeman et al. (2008)[64] | Down syndrome | NR | Infants | Medical record/ registry | Point | 18.58% |
| Atrial septal defect | Bergström et al. (2016)[62] | Down syndrome | NR | Infants | Medical record/ registry | Period | 8.66% |
| Atrial septal defect | Jaruratanasirikul et al. (2017)[12] | Down syndrome | NR | Infants | Medical record/ registry | Period | 8.72% |
| Atrial septal defect | Kim et al. (2014)[58] | Down syndrome | NR | ≤1 | Medical record/ registry | Period | 9.90% |
| Atrial septal defect | Santoro et al. (2018)[55] | Down syndrome | NR | Infants | Medical record/ registry | Period | 15.22% |
| Atrial septal defect | Scott et al. (2014)[60] | Down syndrome | NR | Infants | Clinical examination | Period | 13.21% * |
| Atrial septal defect | Irving et al. (2012)[59] | Down syndrome | NR | Infants | Medical record/ registry | Period | 6.33% |
| Atrial septal defect | Leonard et al. (1999)[6] | Down syndrome | NR | 5-17 | Medical record/ registry | Lifetime | 8.20% |
| Atrial septal defect | McGrother et al. (1990)[7] | Down syndrome | NR | 1-10 | Medical record/  registry | Lifetime | 3.74% * |
| Atrial septal defect | Cho et al. (2020)[57] | Down syndrome | NR | All ages | Medical record/  registry | Period | 28.02% |
| Atrial septal defect (isolated) | Freeman et al. (1998)[63] | Down syndrome | NR | Infants | Medical record/ registry | Point | 3.52% |
| Atrial septal defect (isolated) | Arnell et al. (2012)[8] | Down syndrome | NR | Infants | Medical record/ registry | Lifetime | 17.48% |
| Atrial septal defect (isolated) | Brodwall et al. (2018)[56] | Down syndrome | NR | 0-5 | Medical record/ registry | Period | 10.79% |
| Atrial septal defects and patent foramen ovale | Torfs et al. (1998)[54] | Down syndrome | NR | <1 | Medical record/ registry | Point | 6.29% |
| Atrioventricular septal defect | Freeman et al. (2008)[64] | Down syndrome | NR | Infants | Medical record/ registry | Point | 17.15% |
| Atrioventricular septal defect | Freeman et al. (1998)[64] | Down syndrome | NR | Infants | Medical record/ registry | Point | 19.82% |
| Atrioventricular septal defect | Torfs et al. (1998)[54] | Down syndrome | NR | <1 | Medical record/ registry | Point | 17.14% |
| Atrioventricular septal defect | Bergström et al. (2016)[62] | Down syndrome | NR | Infants | Medical record/ registry | Period | 22.49% |
| Atrioventricular septal defect | Brodwall et al. (2018)[56] | Down syndrome | NR | 0-5 | Medical record/ registry | Period | 20.30% |
| Atrioventricular septal defect | Jaruratanasirikul et al. (2017)[12] | Down syndrome | NR | Infants | Medical record/ registry | Period | 8.72% |
| Atrioventricular septal defect | Kim et al. (2014)[58] | Down syndrome | NR | ≤1 | Medical record/ registry | Period | 7.36% |
| Atrioventricular septal defect | Santoro et al. (2018)[55] | Down syndrome | NR | Infants | Medical record/ registry | Period | 17.39% |
| Atrioventricular septal defect | Scott et al. (2014)[60] | Down syndrome | NR | Infants | Clinical examination | Period | 43.40% * |
| Atrioventricular septal defect | Arnell et al. (2012)[8] | Down syndrome | NR | Infants | Medical record/ registry | Lifetime | 13.11% |
| Atrioventricular septal defect | Cho et al. (2020)[57] | Down syndrome | NR | All ages | Medical record/  registry | Period | 9.82% |
| Atrioventricular septal defect (complete) | Irving et al. (2012)[59] | Down syndrome | NR | Infants | Medical record/ registry | Period | 15.23% |
| Atrioventricular septal defect (isolated) | Weijerman et al. (2010)[53] | Down syndrome | NR | Infants | Medical record/ registry | Point | 19.09% * |
| Atrioventricular septal defect (partial) | Irving et al. (2012)[59] | Down syndrome | NR | Infants | Medical record/ registry | Period | 2.68% |
| Congenital malformation of cardiac septum, not specified | Santoro et al. (2018)[55] | Down syndrome | NR | Infants | Medical record/ registry | Period | 0.43% * |
| Heart septal defects | Brodwall et al. (2018)[56] | Down syndrome | NR | 0-5 | Medical record/ registry | Period | 25.74% |
| Heart septal defects | Henderson et al. (2007)[14] | Down syndrome | NR | 18-61 | Medical record/ registry | Point | 7.81% * |
| Tetralogy of Fallot | Freeman et al. (2008)[64] | Down syndrome | NR | Infants | Medical record/ registry | Point | 2.65% |
| Tetralogy of Fallot | Arnell et al. (2012)[8] | Down syndrome | NR | Infants | Medical record/ registry | Lifetime | 1.46% |
| Tetralogy of Fallot | Brodwall et al. (2018)[56] | Down syndrome | NR | 0-5 | Medical record/ registry | Period | 1.12% |
| Tetralogy of Fallot | Irving et al. (2012)[59] | Down syndrome | NR | Infants | Medical record/ registry | Period | 1.95% |
| Tetralogy of Fallot | Jaruratanasirikul et al. (2017)[12] | Down syndrome | NR | Infants | Medical record/ registry | Period | 2.68% |
| Tetralogy of Fallot | Kim et al. (2014)[58] | Down syndrome | NR | ≤1 | Medical record/ registry | Period | 2.54% |
| Tetralogy of Fallot | Leonard et al. (1999)[6] | Down syndrome | NR | 5-17 | Medical record/ registry | Lifetime | 0.10% |
| Tetralogy of Fallot | Santoro et al. (2018)[55] | Down syndrome | NR | Infants | Medical record/ registry | Period | 1.74% |
| Tetralogy of Fallot | Scott et al. (2014)[60] | Down syndrome | NR | Infants | Clinical examination | Period | 11.32% * |
| Tetralogy of Fallot | Torfs et al. (1998)[54] | Down syndrome | NR | <1 | Medical record/ registry | Point | 2.52% |
| Tetralogy of Fallot | Weijerman et al. (2010)[53] | Down syndrome | NR | Infants | Medical record/ registry | Point | 2.30% * |
| Tetralogy of Fallot | Henderson et al. (2007)[14] | Down syndrome | NR | 18-61 | Medical record/ registry | Point | 3.13% * |
| Tetralogy of Fallot | Cho et al. (2020)[57] | Down syndrome | NR | All ages | Medical record/  registry | Period | 2.84% |
| Tetralogy of Fallot  (without AVSD) | Freeman et al. (1998)[63] | Down syndrome | NR | Infants | Medical record/ registry | Point | 1.76% |
| Ventricular septal defect | Freeman et al. (2008)[64] | Down syndrome | NR | Infants | Medical record/ registry | Point | 19.20% |
| Ventricular septal defect | Freeman et al. (1998)[63] | Down syndrome | NR | Infants | Medical record/ registry | Point | 15.42% |
| Ventricular septal defect | Torfs et al. (1998)[54] | Down syndrome | NR | <1 | Medical record/ registry | Point | 6.37% |
| Ventricular septal defect | Bergström et al. (2016)[62] | Down syndrome | NR | Infants | Medical record/ registry | Period | 11.86% |
| Ventricular septal defect | Irving et al. (2012)[59] | Down syndrome | NR | Infants | Medical record/ registry | Period | 12.91% |
| Ventricular septal defect | Jaruratanasirikul et al. (2017) [12] | Down syndrome | NR | Infants | Medical record/ registry | Period | 10.74% |
| Ventricular septal defect | Kim et al. (2014)[58] | Down syndrome | NR | ≤1 | Medical record/ registry | Period | 4.06% |
| Ventricular septal defect | Santoro et al. (2018)[55] | Down syndrome | NR | Infants | Medical record/ registry | Period | 20.00% |
| Ventricular septal defect | Scott et al. (2014)[60] | Down syndrome | NR | Infants | Clinical examination | Period | 15.09% * |
| Ventricular septal defect | Arnell et al. (2012)[8] | Down syndrome | NR | Infants | Medical record/ registry | Lifetime | 11.65% |
| Ventricular septal defect | Leonard et al. (1999)[6] | Down syndrome | NR | 5-17 | Medical record/ registry | Lifetime | 13.90% |
| Ventricular septal defect | McGrother et al. (1990)[7] | Down syndrome | NR | 1-10 | Medical record/ registry | Lifetime | 10.28% * |
| Ventricular septal defect | Cho et al. (2020)[57] | Down syndrome | NR | All ages | Medical record/  registry | Period | 19.88% |
| Ventricular septal defect (isolated) | Weijerman et al. (2010)[53] | Down syndrome | NR | Infants | Medical record/ registry | Point | 7.88% * |
| Ebstein's anomaly | Brodwall et al. (2018)[56] | Down syndrome | NR | 0-5 | Medical record/ registry | Period | 0.08% |
| Hypoplastic left ventricle | Torfs et al. (1998)[54] | Down syndrome | NR | <1 | Medical record/ registry | Point | 0.66% |
| Hypoplastic right heart | Torfs et al. (1998)[54] | Down syndrome | NR | <1 | Medical record/ registry | Point | 0.80% |
| Pulmonary valve atresia | Santoro et al. (2018) [55] | Down syndrome | NR | Infants | Medical record/ registry | Period | 0.43% * |
| Pulmonary valve atresia/stenosis | Kim et al. (2014)[58] | Down syndrome | NR | ≤1 | Medical record/ registry | Period | 0.00% |
| Pulmonary valve atresia/stenosis | Cho et al. (2020)[57] | Down syndrome | NR | All ages | Medical record/  registry | Period | 0.58% |
| Pulmonary valve defects | Torfs et al. (1998)[54] | Down syndrome | NR | <1 | Medical record/ registry | Point | 0.49% |
| Tricuspid atresia and stenosis | Santoro et al. (2018) [55] | Down syndrome | NR | Infants | Medical record/ registry | Period | 0.87% * |
| Tricuspid valve defects | Torfs et al. (1998)[54] | Down syndrome | NR | <1 | Medical record/ registry | Point | 11.44% |
| Ebstein's anomaly | Santoro et al. (2018)[55] | Down syndrome | NR | Infants | Medical record/ registry | Period | 0.43% * |
| Aortic valve defects | Torfs et al. (1998)[54] | Down syndrome | NR | <1 | Medical record/ registry | Point | 2.59% |
| Cleft atrioventricular valve | Scott et al. (2014)[60] | Down syndrome | NR | Infants | Clinical examination | Period | 3.77% * |
| Congenital insufficiency of aortic valve | Santoro et al. (2018)[55] | Down syndrome | NR | Infants | Medical record/ registry | Period | 0.43% * |
| Hypoplastic left heart syndrome | Torfs et al. (1998)[54] | Down syndrome | NR | <1 | Medical record/ registry | Point | 0.04% |
| Mitral valve anomalies | Santoro et al. (2018)[55] | Down syndrome | NR | Infants | Medical record/ registry | Period | 0.87% * |
| Mitral valve defects | Torfs et al. (1998)[54] | Down syndrome | NR | <1 | Medical record/ registry | Point | 4.87% |
| Aortic valve stenosis | Brodwall et al. (2018)[56] | Down syndrome | NR | 0-5 | Medical record/ registry | Period | 0.08% |
| Anomalies of the coronary artery or sinus | Torfs et al. (1998)[54] | Down syndrome | NR | <1 | Medical record/ registry | Point | 0.62% |
| Congenital valvular disease | Henderson et al. (2007)[14] | Down syndrome | NR | 18-61 | Medical record/ registry | Point | 3.13% * |
| Dextrocardia | Torfs et al. (1998)[54] | Down syndrome | NR | <1 | Medical record/ registry | Point | 0.07% |
| Anomalies of the pulmonary artery | Torfs et al. (1998)[54] | Down syndrome | NR | <1 | Medical record/ registry | Point | 2.42% |
| Aortic atresia/interrupted aortic arch | Santoro et al. (2018)[55] | Down syndrome | NR | Infants | Medical record/ registry | Period | 0.43% * |
| Aortic hypoplasia | Brodwall et al. (2018)[56] | Down syndrome | NR | 0-5 | Medical record/ registry | Period | 0.56% |
| Coarctation of aorta | Kim et al. (2014)[58] | Down syndrome | NR | ≤1 | Medical record/ registry | Period | 0.51% |
| Coarctation of aorta | Santoro et al. (2018)[55] | Down syndrome | NR | Infants | Medical record/ registry | Period | 1.30% * |
| Coarctation of aorta | Scott et al. (2014)[60] | Down syndrome | NR | Infants | Clinical examination | Period | 1.89% * |
| Coarctation of aorta | Brodwall et al. (2018)[56] | Down syndrome | NR | 0-5 | Medical record/ registry | Period | 0.80% |
| Coarctation of aorta | Torfs et al. (1998)[54] | Down syndrome | NR | <1 | Medical record/ registry | Point | 0.97% |
| Coarctation of aorta | Cho et al. (2020)[57] | Down syndrome | NR | All ages | Medical record/  registry | Period | 0.77% |
| Hypoplasia of the aorta | Torfs et al. (1998)[54] | Down syndrome | NR | <1 | Medical record/ registry | Point | 2.21% |
| Interrupted aortic arch | Torfs et al. (1998)[54] | Down syndrome | NR | <1 | Medical record/ registry | Point | 0.07% |
| Overriding aorta | Torfs et al. (1998)[54] | Down syndrome | NR | <1 | Medical record/ registry | Point | 0.35% |
| Patent ductal arteriosus | Leonard et al. (1999)[6] | Down syndrome | NR | 5-17 | Medical record/registry | Lifetime | 2.40% |
| Patent ductal arteriosus | McGrother et al. (1990)[7] | Down syndrome | NR | 1-10 | Medical record/ registry | Lifetime | 8.41% * |
| Patent ductus arteriosus | Arnell et al. (2012)[8] | Down syndrome | NR | Infants | Medical record/ registry | Lifetime | 4.85% |
| Patent ductal arteriosus | Jaruratanasirikul et al. (2017)[12] | Down syndrome | NR | Infants | Medical record/ registry | Period | 9.40% |
| Patent ductal arteriosus | Kim et al. (2014)[58] | Down syndrome | NR | ≤1 | Medical record/ registry | Period | 4.82% |
| Patent ductal arteriosus | Scott et al. (2014)[60] | Down syndrome | NR | Infants | Clinical examination | Period | 41.51% * |
| Patent ductus arteriosus | Bergström et al. (2016)[62] | Down syndrome | NR | Infants | Medical record/ registry | Period | 2.70% |
| Patent ductus arteriosus | Irving et al. (2012)[59] | Down syndrome | NR | Infants | Medical record/ registry | Period | 1.71% |
| Patent ductal arteriosus | Torfs et al. (1998)[54] | Down syndrome | NR | <1 | Medical record/ registry | Point | 24.40% |
| Patent ductal arteriosus (isolated) | Weijerman et al. (2010)[53] | Down syndrome | NR | Infants | Medical record/ registry | Point | 2.50% * |
| Patent ductus arteriosus (persistent) | Freeman et al. (1998)[63] | Down syndrome | NR | Infants | Medical record/ registry | Point | 3.08% |
| Patent ductus arteriosus | Henderson et al. (2007)[14] | Down syndrome | NR | 18-61 | Medical record/ registry | Point | 1.56% * |
| Persistent ductus arteriosus (isolated) | Brodwall et al. (2018)[56] | Down syndrome | NR | 0-5 | Medical record/ registry | Period | 4.80% |
| Persistent right aortic arch | Torfs et al. (1998)[54] | Down syndrome | NR | <1 | Medical record/ registry | Point | 0.48% |
| Right pulmonary artery stenosis | Scott et al. (2014)[60] | Down syndrome | NR | Infants | Clinical examination | Period | 1.89% * |
| Anomalies of the great veins | Torfs et al. (1998)[54] | Down syndrome | NR | <1 | Medical record/ registry | Point | 1.35% |
| Single umbilical artery | Torfs et al. (1998)[54] | Down syndrome | NR | <1 | Medical record/ registry | Point | 0.76% |
| Choanal atresia | Fabia et al. (1970)[5] | Down syndrome | NR | Young children | Medical record/ registry | Period | 0.17% * |
| Choanal atresia | Torfs et al. (1998)[54] | Down syndrome | NR | <1 | Medical record/ registry | Point | 0.07% |
| Congenital subglottic stenosis | Hamilton et al. (2016)[25] | Down syndrome | NR | 0-20 | Medical record/ registry | Period | 1.26% |
| Laryngomalacia | Hamilton et al. (2016)[25] | Down syndrome | NR | 0-20 | Medical record/ registry | Period | 0.84% |
| Anomalies of the larynx, trachea, or bronchus | Torfs et al. (1998)[54] | Down syndrome | NR | <1 | Medical record/ registry | Point | 1.42% |
| Tracheal stenosis | Fabia et al. (1970)[5] | Down syndrome | NR | Young children | Medical record/ registry | Period | 0.08% * |
| Anomalies of the lung | Torfs et al. (1998)[54] | Down syndrome | NR | <1 | Medical record/ registry | Point | 1.07% |
| Cleft lip/palate defect | McGrother et al. (1990)[7] | Down syndrome | NR | 1-10 | Medical record/ registry | Lifetime | 1.87% * |
| Cleft palate and/or lip | Fabia et al. (1970)[5] | Down syndrome | NR | Young children | Medical record/ registry | Period | 0.50% * |
| Cleft palate without cleft lip | Torfs et al. (1998)[54] | Down syndrome | NR | <1 | Medical record/ registry | Point | 0.31% |
| Cleft lip with or without cleft palate and without holoprosencephaly | Torfs et al. (1998)[54] | Down syndrome | NR | <1 | Medical record/ registry | Point | 0.04% |
| Congenital defects of digestive system | Torfs et al. (1998)[54] | Down syndrome | NR | <1 | Medical record/ registry | Point | 10.89% |
| Esophageal atresia ± tracheoesophageal | Freeman et al. (2009)[65] | Down syndrome | NR | Infants | Medical record/ registry | Point | 0.42% |
| Gastrointestinal defects | Freeman et al. (2009)[65] | Down syndrome | NR | Infants | Medical record/ registry | Point | 6.66% |
| Gastrointestinal defects | Jaruratanasirikul et al. (2017)[12] | Down syndrome | NR | Infants | Medical record/ registry | Period | 6.71% |
| Intestinal atresia/obstruction | McGrother et al. (1990)[7] | Down syndrome | NR | 1-10 | Medical record/ registry | Lifetime | 2.80% * |
| Abnormality of oesophagus | McGrother et al. (1990)[7] | Down syndrome | NR | 1-10 | Medical record/ registry | Lifetime | 0.93% * |
| Tracheo-esophageal fistula | Jaruratanasirikul et al. (2017)[12] | Down syndrome | NR | Infants | Medical record/ registry | Period | 0.67% |
| Tracheo-esophageal fistula or esophageal atresia | Fabia et al. (1970)[5] | Down syndrome | NR | Young children | Medical record/ registry | Period | 0.29% * |
| Tracheo-esophageal fistula, esophageal, atresia/stenosis | Torfs et al. (1998)[54] | Down syndrome | NR | <1 | Medical record/ registry | Point | 0.69% |
| Pyloric stenosis | Fabia et al. (1970)[5] | Down syndrome | NR | Young children | Medical record/ registry | Period | 0.17% * |
| Pyloric stenosis | Freeman et al. (2009)[65] | Down syndrome | NR | Infants | Medical record/ registry | Point | 0.32% |
| Pyloric stenosis | Torfs et al. (1998)[54] | Down syndrome | NR | <1 | Medical record/ registry | Point | 0.17% |
| Duodenal atresia | Arnell et al. (2012)[8] | Down syndrome | NR | Infants | Medical record/ registry | Lifetime | 1.94% |
| Duodenal atresia | Leonard et al. (1999)[6] | Down syndrome | NR | 5-17 | Self or informant-reported | Lifetime | 1.90% |
| Duodenal atresia | Jaruratanasirikul et al. (2017) [12] | Down syndrome | NR | Infants | Medical record/ registry | Period | 2.01% |
| Duodenal stenosis | Arnell et al. (2012)[8] | Down syndrome | NR | Infants | Medical record/ registry | Lifetime | 3.40% |
| Jejunal stenosis | Arnell et al. (2012)[8] | Down syndrome | NR | Infants | Medical record/ registry | Lifetime | 0.49% |
| Atresia and stenosis of small intestine | Torfs et al. (1998)[54] | Down syndrome | NR | <1 | Medical record/ registry | Point | 4.80% |
| Duodenal obstruction | Fabia et al. (1970)[5] | Down syndrome | NR | Young children | Medical record/ registry | Period | 2.60% * |
| Duodenal stenosis/atresia ± annular pancreas | Freeman et al. (2009)[65] | Down syndrome | NR | Infants | Medical record/ registry | Point | 3.91% |
| Ileal atresia | Fabia et al. (1970)[5] | Down syndrome | NR | Young children | Medical record/ registry | Period | 0.08% * |
| Anal atresia | Arnell et al. (2012)[8] | Down syndrome | NR | Infants | Medical record/ registry | Lifetime | 0.49% |
| Anal atresia/stenosis or missing or imperforate anus | Torfs et al. (1998)[54] | Down syndrome | NR | <1 | Medical record/ registry | Point | 1.38% |
| Anal stenosis/atresia | Freeman et al. (2009)[65] | Down syndrome | NR | Infants | Medical record/ registry | Point | 0.95% |
| Imperforate anus | Jaruratanasirikul et al. (2017)[12] | Down syndrome | NR | Infants | Medical record/ registry | Period | 2.68% |
| Imperforate anus | Fabia et al. (1970)[5] | Down syndrome | NR | Young children | Medical record/ registry | Period | 0.70% * |
| Imperforate anus | McGrother et al. (1990)[7] | Down syndrome | NR | 1-10 | Medical record/ registry | Lifetime | 0.93% * |
| Congenital megacolon | Fabia et al. (1970)[5] | Down syndrome | NR | Young children | Medical record/ registry | Period | 0.37% * |
| Ectopic anus | Torfs et al. (1998)[54] | Down syndrome | NR | <1 | Medical record/ registry | Point | 1.11% |
| Hirschsprung disease | McGrother et al. (1990)[7] | Down syndrome | NR | 1-10 | Medical record/ registry | Lifetime | 0.93% * |
| Hirschsprung disease | Arnell et al. (2012)[8] | Down syndrome | NR | Infants | Medical record/ registry | Lifetime | 0.97% |
| Hirschsprung disease | Leonard et al. (1999)[6] | Down syndrome | NR | 5-17 | Self or informant-reported | Lifetime | 0.10% |
| Hirschsprung disease | Jaruratanasirikul et al. (2017)[12] | Down syndrome | NR | Infants | Medical record/ registry | Period | 0.67% |
| Hirschsprung disease | Freeman et al. (2009)[65] | Down syndrome | NR | Infants | Medical record/ registry | Point | 0.85% |
| Hirschsprung disease | Torfs et al. (1998)[54] | Down syndrome | NR | <1 | Medical record/ registry | Point | 1.38% |
| Malrotation of the intestines | Torfs et al. (1998)[54] | Down syndrome | NR | <1 | Medical record/ registry | Point | 1.52% |
| Malrotation small and/or large bowel | Fabia et al. (1970)[5] | Down syndrome | NR | Young children | Medical record/ registry | Period | 0.21% * |
| Atresia bile duct | Fabia et al. (1970)[5] | Down syndrome | NR | Young children | Medical record/ registry | Period | 0.21% * |
| Bile duct atresia | Torfs et al. (1998)[54] | Down syndrome | NR | <1 | Medical record/ registry | Point | 0.07% |
| Congenital obstruction bladder | Fabia et al. (1970)[5] | Down syndrome | NR | Young children | Medical record/ registry | Period | 0.08% * |
| Annular pancreas | Torfs et al. (1998)[54] | Down syndrome | NR | <1 | Medical record/ registry | Point | 1.42% |
| Cryptorchidism | Diene et al. (2010)[15] | Prader-Willi syndrome | NR | ≤18 | Medical record/ registry | Point | 87.84% |
| Undescended testicles | Torfs et al. (1998)[54] | Down syndrome | NR | <1 | Medical record/ registry | Point | 7.22% |
| Rectovaginal fistula | Arnell et al. (2012)[8] | Down syndrome | NR | Infants | Medical record/ registry | Lifetime | 0.49% |
| Hypospadias | Kupferman et al. (2009)[66] | Down syndrome | NR | Infants | Medical record/ registry | Period | 0.81% * |
| Hypoplasia or absence- kidney | Fabia et al. (1970)[5] | Down syndrome | NR | Young children | Medical record/ registry | Period | 0.17% * |
| Renal agenesis | Kupferman et al. (2009)[66] | Down syndrome | NR | Infants | Medical record/ registry | Period | 0.23% * |
| Renal and urinary tract anomalies | Kupferman et al. (2009)[66] | Down syndrome | NR | Infants | Medical record/ registry | Period | 3.20% * |
| Cystic kidney | Fabia et al. (1970)[5] | Down syndrome | NR | Young children | Medical record/ registry | Period | 0.08% * |
| Hydroureters and hydronephrosis | Fabia et al. (1970)[5] | Down syndrome | NR | Young children | Medical record/ registry | Period | 0.17% * |
| Obstructive defects of renal pelvis | Torfs et al. (1998)[54] | Down syndrome | NR | <1 | Medical record/ registry | Point | 1.83% |
| Urinary tract obstructive defect | Kupferman et al. (2009)[66] | Down syndrome | NR | Infants | Medical record/ registry | Period | 2.06% * |
| Horseshoe kidney | Torfs et al. (1998)[54] | Down syndrome | NR | <1 | Medical record/ registry | Point | 0.07% |
| Absence of bladder or urethra | Torfs et al. (1998)[54] | Down syndrome | NR | <1 | Medical record/ registry | Point | 0.00% |
| Exstrophy of urinary bladder | Torfs et al. (1998)[54] | Down syndrome | NR | <1 | Medical record/ registry | Point | 0.00% |
| Congenital dislocation of hips | Fabia et al. (1970)[5] | Down syndrome | NR | Young children | Medical record/ registry | Period | 0.17% * |
| Chondro dystrophy | McGrother et al. (1990)[7] | Down syndrome | NR | 1-10 | Medical record/ registry | Lifetime | 0.93% * |
| Upper or lower limb reduction defects | Torfs et al. (1998)[54] | Down syndrome | NR | <1 | Medical record/ registry | Point | 1.31% |
| Clubfoot | Torfs et al. (1998)[54] | Down syndrome | NR | <1 | Medical record/ registry | Point | 0.93% |
| Clubfoot | Fabia et al. (1970)[5] | Down syndrome | NR | Young children | Medical record/ registry | Period | 1.12% * |
| Bifid thumb | Fabia et al. (1970)[5] | Down syndrome | NR | Young children | Medical record/ registry | Period | 0.21% * |
| Polydactyly | Fabia et al. (1970)[5] | Down syndrome | NR | Young children | Medical record/ registry | Period | 0.08% * |
| Polydactyly | Torfs et al. (1998)[54] | Down syndrome | NR | <1 | Medical record/ registry | Point | 0.93% |
| Syndactyly | Torfs et al. (1998)[54] | Down syndrome | NR | <1 | Medical record/ registry | Point | 2.45% |
| Syndactyly or webbed fingers and/or toes | Fabia et al. (1970)[5] | Down syndrome | NR | Young children | Medical record/ registry | Period | 0.62% * |
| Amputation forearms and/or hands | Fabia et al. (1970)[5] | Down syndrome | NR | Young children | Medical record/ registry | Period | 0.12% * |
| Partial or total adactyly | Fabia et al. (1970)[5] | Down syndrome | NR | Young children | Medical record/ registry | Period | 0.25% * |
| Artlirogryposis | Fabia et al. (1970)[5] | Down syndrome | NR | Young children | Medical record/ registry | Period | 0.08% * |
| Macrocephaly | Christianson et al. (2002)[32] | Not specified | Severe - profound | 2-9 | Clinical examination | Point | 0.00% |
| Diaphragmatic hernia | Arnell et al. (2012)[8] | Down syndrome | NR | Infants | Medical record/ registry | Lifetime | 0.49% |
| Diaphragmatic hernia | Fabia et al. (1970)[5] | Down syndrome | NR | Young children | Medical record/ registry | Period | 0.17% * |
| Gastroschisis | Torfs et al. (1998)[54] | Down syndrome | NR | <1 | Medical record/ registry | Point | 0.00% |
| Morgagni hernia | Torfs et al. (1998)[54] | Down syndrome | NR | <1 | Medical record/ registry | Point | 0.14% |
| Omphalocele | Arnell et al. (2012)[8] | Down syndrome | NR | Infants | Medical record/ registry | Lifetime | 1.46% |
| Omphalocele | Torfs et al. (1998)[54] | Down syndrome | NR | <1 | Medical record/ registry | Point | 0.03% |
| Other diaphragmatic hernias | Torfs et al. (1998)[54] | Down syndrome | NR | <1 | Medical record/ registry | Point | 0.07% |
| Tuberous sclerosis | Christianson et al. (2002)[32] | Not specified | Severe - profound | 2-9 | Clinical examination | Point | 2.33% |
| Injury, poisoning and certain other consequences of external causes (S00-T98) |  |  |  |  |  |  |  |
| Traumatic brain injury | Bishop (2020)[39] | Not specified | All | ≥21 | Medical record/ registry | Period | 1.38% |

* The estimates were calculated by the authors of the systematic review based on data reported in the paper.

Abbreviation: NR: not reported.

## **Table S10. Estimates of incidence of physical disorders (/10,000 person years for rate and percentage for incidence proportion) in people with ID**

| Disorders | Study | Genetic syndromes | ID levels | ID age range | Outcome ascertainment | Estimate in ID |
| --- | --- | --- | --- | --- | --- | --- |
| Neoplasms (C00-D48) | |  |  |  |  |  |
| All cancers | Patja et al. (2001)[67] | Not specified | Mild - moderate | 7-69 | Medical record/ registry | 33.12 |
| All cancers | Sullivan et al. (2004)[68] | Not specified | NR | 30.8 (16.3) (mean, sd) | Medical record/ registry | 12.76 |
| All cancers | Hasle et al. (2016)[69] | Down syndrome | NR | All ages | Medical record/ registry | 12.62 |
| All cancers | Patja et al. (2006)[70] | Down syndrome | NR | 0-79 | Medical record/ registry | 9.12 |
| All cancers | Boker et al. (2001)[71] | Down syndrome | NR | <17 | Medical record/ registry | 6.38 |
| All cancers | Sullivan et al. (2007)[72] | Down syndrome | NR | Baseline: 20.2 (13.9) (mean, sd) | Medical record/ registry | 11.02 |
| All cancers | Patja et al. (2008)[73] | Prader-Willi syndrome | NR | 0-79 | Medical record/ registry | 29 |
| All cancers | Hjortshøj et al. (2007)[74] | Bardet-Biedl syndrome | NR | All ages | Medical record/ registry | 8.31 |
| All cancers | Schultz-Pedersen et al. (2001)[75] | Fragile X syndrome | NR | All ages | Medical record/ registry | 1.35% |
| All solid tumours | Hasle et al. (2016)[69] | Down syndrome | NR | All ages | Medical record/ registry | 6.14 |
| All solid tumours | Sullivan et al. (2007)[72] | Down syndrome | NR | Baseline: 20.2 (13.9) (mean, sd) | Medical record/ registry | 4.2 |
| Gastrointestinal system | Patja et al. (2001)[67] | Not specified | Mild - moderate | 7-69 | Medical record/ registry | 10.15 |
| Digestive system | Hasle et al. (2016)[69] | Down syndrome | NR | All ages | Medical record/ registry | 0.89 |
| Buccal cavity | Hasle et al. (2016)[69] | Down syndrome | NR | All ages | Medical record/ registry | 0 |
| Otorhinolaryngeal | Patja et al. (2001)[67] | Not specified | Mild - moderate | 7-69 | Medical record/ registry | 1.34 |
| Stomach | Sullivan et al. (2004)[68] | Not specified | NR | 30.8 (16.3) (mean, sd) | Medical record/ registry | 0.51 |
| Colorectal | Sullivan et al. (2004)[68] | Not specified | NR | 30.8 (16.3) (mean, sd) | Medical record/ registry | 1.4 |
| Gallbladder | Patja et al. (2001)[67] | Not specified | Mild - moderate | 7-69 | Medical record/ registry | 1.34 |
| Pancreas | Schultz-Pedersen et al. (2001)[75] | Fragile X syndrome | NR | All ages | Medical record/ registry | 0.45% |
| Respiratory system | Patja et al. (2001)[67] | Not specified | Mild - moderate | 7-69 | Medical record/ registry | 3.83 |
| Respiratory system | Hasle et al. (2016)[69] | Down syndrome | NR | All ages | Medical record/ registry | 0.11 |
| Lung | Sullivan et al. (2004)[68] | Not specified | NR | 30.8 (16.3) (mean, sd) | Medical record/ registry | 0.45 |
| Bone | Patja et al. (2001)[67] | Not specified | Mild - moderate | 7-69 | Medical record/ registry | 0 |
| Bone and cartilage | Hasle et al. (2016)[69] | Down syndrome | NR | All ages | Medical record/ registry | 0 |
| Skin (melanoma) | Patja et al. (2001)[67] | Not specified | Mild - moderate | 7-69 | Medical record/ registry | 0.57 |
| Skin (melanoma) | Sullivan et al. (2004)[68] | Not specified | NR | 30.8 (16.3) (mean, sd) | Medical record/ registry | 1.47 |
| Skin (melanoma) | Hasle et al. (2016)[69] | Down syndrome | NR | All ages | Medical record/ registry | 0.22 |
| Skin (non-melanoma) | Hasle et al. (2016)[69] | Down syndrome | NR | All ages | Medical record/ registry | 0.67 |
| Mesothelioma and soft tissue | Hasle et al. (2016)[69] | Down syndrome | NR | All ages | Medical record/ registry | 0.33 |
| Connective tissue | Patja et al. (2001)[67] | Not specified | Mild - moderate | 7-69 | Medical record/ registry | 0.77 |
| Breast | Patja et al. (2001)[67] | Not specified | Mild - moderate | 7-69 | Medical record/ registry | 4.4 |
| Breast | Sullivan et al. (2004)[68] | Not specified | NR | 30.8 (16.3) (mean, sd) | Medical record/ registry | 1.34 |
| Breast | Hasle et al. (2016)[69] | Down syndrome | NR | All ages | Medical record/ registry | 0.33 |
| Female genital organs | Patja et al. (2001)[67] | Not specified | Mild - moderate | 7-69 | Medical record/ registry | 3.25 |
| Female genital organs | Hasle et al. (2016)[69] | Down syndrome | NR | All ages | Medical record/ registry | 0.56 |
| Cervix uteri | Sullivan et al. (2004)[68] | Not specified | NR | 30.8 (16.3) (mean,sd) | Medical record/ registry | 0.13 |
| Corpus uteri | Sullivan et al. (2004)[68] | Not specified | NR | 30.8 (16.3) (mean,sd) | Medical record/ registry | 0.51 |
| Corpus uterus | Schultz-Pedersen et al. (2001)[75] | Fragile X syndrome | NR | All ages | Medical record/ registry | 0.45% |
| Ovary | Sullivan et al. (2004)[68] | Not specified | NR | 30.8 (16.3) (mean,sd) | Medical record/ registry | 0.19 |
| Male genital organs | Patja et al. (2001)[67] | Not specified | Mild - moderate | 7-69 | Medical record/ registry | 1.15 |
| Prostate gland | Sullivan et al. (2004)[68] | Not specified | NR | 30.8 (16.3) (mean,sd) | Medical record/ registry | 0.13 |
| Testicular | Sullivan et al. (2004)[68] | Not specified | NR | 30.8 (16.3) (mean,sd) | Medical record/ registry | 0.45 |
| Testicular | Hasle et al. (2016)[69] | Down syndrome | NR | All ages | Medical record/ registry | 1.56 |
| Testicular (Sweden) | Bjørge et al. (2008)[76] | Down syndrome | NR | <40 | Medical record/ registry | 0.06% from 1973 to 2004 |
| Testicular (Norway) | Bjørge et al. (2008)[76] | Down syndrome | NR | <40 | Medical record/ registry | 0.24% from 1967 to 2004 |
| Urinary tract | Patja et al. (2001)[67] | Not specified | Mild - moderate | 7-69 | Medical record/ registry | 0.77 |
| Urinary tract | Hasle et al. (2016)[69] | Down syndrome | NR | All ages | Medical record/ registry | 0.56 |
| Urinary bladder | Schultz-Pedersen et al. (2001)[75] | Fragile X syndrome | NR | All ages | Medical record/ registry | 0.45% |
| Eye | Patja et al. (2001)[67] | Not specified | Mild - moderate | 7-69 | Medical record/ registry | 0 |
| Eye, brain and central nervous system | Hasle et al. (2016)[69] | Down syndrome | NR | All ages | Medical record/ registry | 0.56 |
| Nervous system | Patja et al. (2001)[67] | Not specified | Mild - moderate | 7-69 | Medical record/ registry | 1.53 |
| Brain | Sullivan et al. (2004)[68] | Not specified | NR | 30.8 (16.3) (mean,sd) | Medical record/ registry | 0.83 |
| Thyroid glands | Patja et al. (2001)[67] | Not specified | Mild - moderate | 7-69 | Medical record/ registry | 1.34 |
| Thyroid glands | Sullivan et al. (2004)[68] | Not specified | NR | 30.8 (16.3) (mean,sd) | Medical record/ registry | 0.32 |
| Endocrine organs | Hasle et al. (2016)[69] | Down syndrome | NR | All ages | Medical record/ registry | 0 |
| Lymphomas | Sullivan et al. (2004)[68] | Not specified | NR | 30.8 (16.3) (mean,sd) | Medical record/ registry | 0.32 |
| Hodgkin lymphoma | Hasle et al. (2016)[69] | Down syndrome | NR | All ages | Medical record/ registry | 0.11 |
| Leukaemia | Patja et al. (2001)[67] | Not specified | Mild - moderate | 7-69 | Medical record/ registry | 0.96 |
| Leukaemia | Sullivan et al. (2004)[68] | Not specified | NR | 30.8 (16.3) (mean,sd) | Medical record/ registry | 1.79 |
| Leukaemia | Patja et al. (2006)[70] | Down syndrome | NR | 0-79 | Medical record/ registry | 3.46 |
| Leukaemia (Norway) | Bjørge et al. (2008)[76] | Down syndrome | NR | <40 | Medical record/ registry | 1.99% from 1967 to 2004 |
| Leukaemia (Sweden) | Bjørge et al. (2008)[76] | Down syndrome | NR | <40 | Medical record/ registry | 2.06% from 1973 to 2004 |
| Leukaemia | Sullivan et al. (2007)[72] | Down syndrome | NR | Baseline: 20.2 (13.9) (mean,sd) | Medical record/ registry | 6.82 |
| Leukaemia | Sullivan et al. (2007)[72] | Down syndrome | NR | 0-4 | Medical record/ registry | 46.21 |
| Leukaemia | Boker et al. (2001)[71] | Down syndrome | NR | <17 | Medical record/ registry | 6.38 |
| Lymphomas and leukemia | Hasle et al. (2016)[69] | Down syndrome | NR | All ages | Medical record/ registry | 6.48 |
| Other leukemias | Hasle et al. (2016)[69] | Down syndrome | NR | All ages | Medical record/ registry | 0.56 |
| Non-Hodgkin lymphoma | Patja et al. (2001)[67] | Not specified | Mild - moderate | 7-69 | Medical record/ registry | 1.15 |
| Non-Hodgkin lymphoma | Hasle et al. (2016)[69] | Down syndrome | NR | All ages | Medical record/ registry | 0.22 |
| Myeloma | Patja et al. (2001)[67] | Not specified | Mild - moderate | 7-69 | Medical record/ registry | 0.19 |
| Myeloma | Hasle et al. (2016)[69] | Down syndrome | NR | All ages | Medical record/ registry | 0 |
| Plasma cell tumours | Sullivan et al. (2004)[68] | Not specified | NR | 30.8 (16.3) (mean,sd) | Medical record/ registry | 0.19 |
| Lymphoid leukemia | Hasle et al. (2016)[69] | Down syndrome | NR | All ages | Medical record/ registry | 3.35 |
| Acute lymphoblastic leukemia | Boker et al. (2001)[71] | Down syndrome | NR | <17 | Medical record/ registry | 0.91 |
| Acute lymphoid leukemia (Sweden) | Bjørge et al. (2008)[76] | Down syndrome | NR | <40 | Medical record/ registry | 0.78% from 1973 to 2004 |
| Acute lymphoid leukemia (Norway) | Bjørge et al. (2008)[76] | Down syndrome | NR | <40 | Medical record/ registry | 0.57% from 1967 to 2004 |
| Myeloid leukemia | Hasle et al. (2016)[69] | Down syndrome | NR | All ages | Medical record/ registry | 2.12 |
| Acute myeloid leukemia | Boker et al. (2001)[71] | Down syndrome | NR | <17 | Medical record/ registry | 2.74 |
| Acute myeloid leukemia (Sweden) | Bjørge et al. (2008)[76] | Down syndrome | NR | <40 | Medical record/ registry | 1.09% from 1973 to 2004 |
| Acute myeloid leukemia (Norway) | Bjørge et al. (2008)[76] | Down syndrome | NR | <40 | Medical record/ registry | 1.09% from 1967 to 2004 |
| Monocytoid leukemia | Hasle et al. (2016)[69] | Down syndrome | NR | All ages | Medical record/ registry | 0.11 |
| Hodgkin disease | Patja et al. (2001)[67] | Not specified | Mild - moderate | 7-69 | Medical record/ registry | 0 |
| Diseases of the digestive system (K00-K93) | | |  |  |  |  |
| Celiac disease | Ostermaier et al. (2020)[77] | Down syndrome | NR | All ages (median: 22.9, IQR = 16.6-29.9) | Clinical examination | 32.5 |
| Injury, poisoning and certain other consequences of external causes (S00-T98) | | | | |  |  |
| Injuries (self-injury excluded) | Finlayson et al. (2010)[35] | Not specified | All | 18-64 | Self or informant-reported | 20.40% in a year |

All estimates for cancer were calculated by the author using the data provided in the original studies.

Abbreviations:

NR: not reported

## **Table S11. Comparisons of prevalence between people with ID and people without ID or the general population**

| Disorders (ICD 10 chapter) | Location | Study | Genetic syndrome | Prevalence in ID | Prevalence in comparison group | Measurement | Effect size (95% CI) | Covariates |
| --- | --- | --- | --- | --- | --- | --- | --- | --- |
| Certain infectious and parasitic diseases (A00-B99) | | | | | | | | |
| Viral hepatitis | UK | Cooper et al. (2015)[1] | Not specified | 0.09% | 0.08% | OR | 0.82  (0.39-1.74) | Age, sex and deprivation score |
| HIV | Canada | Lunsky et al. (2017)[2] | Not specified | 0.17% | N/A | RR | 0.95 | Age and sex |
| Neoplasms (C00-D48) | | | | | | | | |
| All cancers (in the last 4 years | UK | Cooper et al. (2018)[3] | Not specified | 0.97% | 0.85% | Rate ratio | 1.14  (0.54-2.39) | None |
| All cancers | UK | Carey et al. (2016)[4] | Not specified | 1.61% | 2.42% | PR | 0.70  (0.61-0.80) | Age, sex and practice |
| Endocrine, nutritional and metabolic diseases (E00-E90) | | | | | | | | |
| Hypothyroidism | UK | Cooper et al. (2018)[3] | Not specified | 5.27% | 2.82% | Rate ratio | 1.87  (1.36-2.57) | None |
| Thyrotoxicosis/thyroid disorders | UK | Cooper et al. (2015)[1] | Not specified | 7.85% | 5.04% | OR | 2.36  (2.17-2.58) | Age, sex and deprivation score |
| Diabetes | UK | Carey et al. (2016)[4] | Not specified | 6.89% | 4.39% | PR | 1.64  (1.53-1.75) | Age, sex and practice |
| Diabetes | UK | Cooper et al. (2018)[3] | Not specified | 6.38% | 3.39% | Rate ratio | 1.88  (1.41-2.51) | None |
| Diabetes | UK | Cooper et al. (2015)[1] | Not specified | 6.63% | 5.25% | OR | 1.63  (1.49-1.79) | Age, sex and deprivation score |
| Diabetes | Netherlands | Cuypers et al. (2021)[16] | Not specified | 9.90% | 6.60% | None | P-value <0.05 | Stratified by sex |
| Diseases of the nervous system (G00-G99) | | | | | | | | |
| Parkinson’s disease and Parkinsonism | UK | Cooper et al. (2015)[1] | Not specified | 0.35% | 0.19% | OR | 2.83  (1.95-4.13) | Age, sex and deprivation score |
| Multiple sclerosis | UK | Cooper et al. (2015)[1] | Not specified | 0.11% | 0.27% | OR | 0.49  (0.25-0.96) | Age, sex and deprivation score |
| Epilepsy | UK | Carey et al. (2016)[4] | Not specified | 18.51% | 0.73% | PR | 25.33  (23.29-27.57) | Age, sex and practice |
| Epilepsy | UK | Cooper et al. (2018)[3] | Not specified | 28.16% | 0.82% | Rate ratio | 34.35  (29.93-39.41) | None |
| Epilepsy | UK | Morgan et al. (2003)[36] | Not specified | 16.10% | N/A | Standardised RR | 23.73  (21.28-28.95) | Age and sex |
| Epilepsy | UK | Cooper et al. (2015)[1] | Not specified | 18.82% | 0.77% | OR | 31.03  (29.23-32.92) | Age, sex and deprivation score |
| Migraine | UK | Cooper et al. (2015)[1] | Not specified | 0.74% | 0.65% | OR | 1.32  (1.02-1.71) | Age, sex and deprivation score |
| Diseases of the eye and adnexa (H00-H59) | | | | | | | | |
| Retinopathy of prematurity | Denmark | Atladottir et al. (2015)[42] | Not specified | 15.60% | 5.60% | NR | NR | None |
| Glaucoma | UK | Cooper et al. (2015)[1] | Not specified | 0.90% | 1.12% | OR | 1.17  (0.92-1.48) | Age, sex and deprivation score |
| Visual impairment | UK | Cooper et al. (2015)[1] | Not specified | 3.22% | 0.57% | OR | 7.81  (6.86-8.89) | Age, sex and deprivation score |
| Diseases of the ear and mastoid process (H60-H95) | | | | | | | | |
| Hearing loss | UK | Cooper et al. (2015)[1] | Not specified | 8.20% | 3.82% | OR | 2.81  (2.59-3.06) | Age, sex and deprivation score |
| Diseases of the circulatory system (I00-I99) | | | | | | | | |
| Hypertension | UK | Carey et al. (2016)[4] | Not specified | 10.73% | 12.08% | PR | 0.93  (0.89-0.98) | Age, sex and practice |
| Hypertension | UK | Cooper et al. (2018)[3] | Not specified | 12.76% | 12.33% | Rate ratio | 1.03  (0.84-1.27) | None |
| Hypertension | UK | Cooper et al. (2015)[1] | Not specified | 9.66% | 16.49% | OR | 0.72  (0.66-0.78) | Age, sex and deprivation score |
| Coronary heart disease | UK | Cooper et al. (2015)[1] | Not specified | 2.00% | 5.74% | OR | 0.43  (0.37-0.51) | Age, sex and deprivation score |
| Coronary heart disease | UK | Cooper et al. (2018)[3] | Not specified | 3.47% | 4.54% | Rate ratio | 0.76  (0.52-1.13) | None |
| Ischemic heart disease | UK | Carey et al. (2016)[4] | Not specified | 1.65% | 2.69% | PR | 0.65  (0.57-0.74) | Age, sex and practice |
| Atrial fibrillation | UK | Carey et al. (2016)[4] | Not specified | 0.83% | 0.95% | PR | 0.91  (0.75-1.09) | Age, sex and practice |
| Atrial fibrillation | UK | Cooper et al. (2018)[3] | Not specified | 0.97% | 1.30% | Rate ratio | 0.75  (0.36-1.56) | None |
| Atrial fibrillation | UK | Cooper et al. (2015)[1] | Not specified | 0.89% | 1.69% | OR | 0.83  (0.61-0.98) | Age, sex and deprivation score |
| Heart failure | UK | Carey et al. (2016)[4] | Not specified | 0.82% | 0.38% | PR | 2.26  (1.84-2.78) | Age, sex and practice |
| Heart failure | UK | Cooper et al. (2018)[3] | Not specified | 2.50% | 0.94% | Rate ratio | 2.67  (1.68-4.24) | None |
| Heart failure | UK | Cooper et al. (2015)[1] | Not specified | 1.02% | 1.33% | OR | 1.11  (0.89-1.43) | Age, sex and deprivation score |
| Stroke | UK | Cooper et al. (2018)[3] | Not specified | 1.80% | 1.96% | Rate ratio | 0.92  (0.53-1.58) | None |
| Peripheral vascular disease | UK | Carey et al. (2016)[4] | Not specified | 0.41% | 0.49% | PR | 0.90  (0.69-1.17) | Age, sex and practice |
| Peripheral vascular disease | UK | Cooper et al. (2015)[1] | Not specified | 0.57% | 1.64% | OR | 0.44  (0.33-0.60) | Age, sex and deprivation score |
| Diseases of the respiratory system (J00-J99) | | | | | | | | |
| Chronic sinusitis | UK | Cooper et al. (2015)[1] | Not specified | 0.30% | 0.65% | OR | 0.44  (0.26-0.62) | Age, sex and deprivation score |
| Chronic obstructive pulmonary disease | UK | Carey et al. (2016)[4] | Not specified | 1.08% | 1.37% | PR | 0.84  (0.71-0.99) | Age, sex and practice |
| Chronic obstructive pulmonary disease | UK | Cooper et al. (2018)[3] | Not specified | 1.25% | 2.20% | Rate ratio | 0.57  (0.29-1.09) | None |
| Chronic obstructive pulmonary disease | UK | Cooper et al. (2015)[1] | Not specified | 2.61% | 3.73% | OR | 0.84  (0.73-0.97) | Age, sex and deprivation score |
| Asthma | UK | Carey et al. (2016)[4] | Not specified | 8.19% | 6.63% | PR | 1.25  (1.18-1.33) | Age, sex and practice |
| Asthma | UK | Cooper et al. (2018)[3] | Not specified | 9.15% | 5.29% | Rate ratio | 1.73  (1.36-2.20) | None |
| Asthma (active) | UK | Cooper et al. (2015)[1] | Not specified | 7.17% | 5.93% | OR | 1.26  (1.16-1.38) | Age, sex and deprivation score |
| Bronchiectasis | UK | Cooper et al. (2015)[1] | Not specified | 0.25% | 0.20% | OR | 1.68  (1.08-2.61) | Age, sex and deprivation score |
| Diseases of the digestive system (K00-K93) | | | | | | | | |
| Inflammatory bowel disease | UK | Cooper et al. (2015)[1] | Not specified | 0.50% | 0.69% | OR | 0.82  (0.60-1.13) | Age, sex and deprivation score |
| Diverticular disease | UK | Cooper et al. (2015)[1] | Not specified | 0.82% | 2.38% | OR | 0.49  (0.39-0.63) | Age, sex and deprivation score |
| Irritable bowel syndrome | UK | Cooper et al. (2015)[1] | Not specified | 3.09% | 3.66% | OR | 0.97  (0.86-1.11) | Age, sex and deprivation score |
| Constipation | UK | Cooper et al. (2015)[1] | Not specified | 13.95% | 2.49% | OR | 11.19  (10.97-12.68) | Age, sex and deprivation score |
| Diseases of the skin and subcutaneous tissue (L00-L99) | | | | | | | | |
| Hidradenitis suppurativa | US | Garg et al. (2018)[23] | Down syndrome | 2.14% | 0.28% | OR | 5.24  (4.62-5.94) | Sex, age, race and obesity |
| Diseases of the musculoskeletal system and connective tissue (M00-M99) | | | | | | | | |
| Arthritis (Inflammatory) | UK | Cooper et al. (2015)[1] | Not specified | 1.88% | 4.08% | OR | 0.57  (0.48-0.67) | Age, sex and deprivation score |
| Rheumatoid arthritis | UK | Carey et al. (2016)[4] | Not specified | 0.49% | 0.64% | PR | 0.82  (0.65-1.05) | Age, sex and practice |
| Osteoporosis | UK | Carey et al. (2016)[4] | Not specified | 1.67% | 0.95% | PR | 1.84  (1.60-2.12) | Age, sex and practice |
| Diseases of the genitourinary system (N00-N99) | | | | | | | | |
| Chronic kidney disease | UK | Carey et al. (2016)[4] | Not specified | 3.17% | 2.03% | PR | 1.64  (1.49-1.82) | Age, sex and practice |
| Chronic kidney disease | UK | Cooper et al. (2018)[3] | Not specified | 2.08% | 1.57% | Rate ratio | 1.33  (0.80-2.20) | None |
| Chronic kidney disease | UK | Cooper et al. (2015)[1] | Not specified | 1.68% | 2.36% | OR | 1.11  (0.93-1.32) | Age, sex and deprivation score |
| Prostate disease | UK | Cooper et al. (2015)[1] | Not specified | 0.51% | 1.07% | OR | 0.60  (0.44-0.82) | Age, sex and deprivation score |
| Certain conditions originating in the perinatal period (P00-P96) | | | | | | | | |
| Persistent pulmonary hypertension of the newborn | Denmark | Atladottir et al. (2015)[42] | Not specified | 0.49% | 0.10% | NR | NR | None |
| Neonatal sepsis | Denmark | Atladottir et al. (2015)[42] | Not specified | 3.88% | 1.44% | NR | NR | None |
| Neonatal hypoglycemia | Denmark | Atladottir et al. (2015)[42] | Not specified | 6.46% | 1.76% | NR | NR | None |
| Congenital malformations, deformations and chromosomal abnormalities (Q00-Q99) | | | | | | | | |
| Anencephalus and spina bifida | US | Torfs et al. (1998)[54] | Down syndrome | 0.00% | 0.06% | RR | N/A | None |
| Encephalocele | US | Torfs et al. (1998)[54] | Down syndrome | 0.04% | 0.01% | RR | 3.7 | None |
| Congenital hydrocephalus | US | Torfs et al. (1998)[54] | Down syndrome | 0.76% | 0.08% | RR | 10.1* | None |
| Holoprosencephaly | US | Torfs et al. (1998)[54] | Down syndrome | 0.10% | 0.01% | RR | 10.1* | None |
| Congenital cataract | US | Torfs et al. (1998)[54] | Down syndrome | 0.93% | 0.02% | RR | 54.3* | None |
| Double outlet right ventricle | Norway | Brodwall et al. (2018)[56] | Down syndrome | 0.16% | 0.01% | PR | 26 (6.5-108) | Maternal age and birth year |
| Double outlet right ventricle | US | Torfs et al. (1998)[54] | Down syndrome | 0.17% | 0.01% | RR | 21.2* | None |
| Double outlet right ventricle | South Korea | Cho et al. (2020)[57] | Down syndrome | 0.63% | 0 | OR | n/a | Age and sex |
| Single ventricle | South Korea | Cho et al. (2020)[57] | Down syndrome | 0.24% | 0 | OR | n/a | Age and sex |
| Transposition of great vessels | US | Torfs et al. (1998)[54] | Down syndrome | 0.07% | 0.03% | RR | 2 | None |
| Transposition of great arteries | South Korea | Cho et al. (2020)[57] | Down syndrome | 0.05% | 0.02% | OR | 2.5 (0.2-27.6) | Age and sex |
| Truncus arteriosus | US | Torfs et al. (1998)[54] | Down syndrome | 0.04% | 0.01% | RR | 4.6 | None |
| Congenital heart defects | Norway | Brodwall et al. (2018)[56] | Down syndrome | 57.90% | 1.24% | PR | 47  (44-49) | Maternal age and birth year |
| Congenital heart defects | US | Torfs et al. (1998)[54] | Down syndrome | 55.98% | 0.52% | RR | 108 | None |
| Atrial septal defect | South Korea | Cho et al. (2020)[57] | Down syndrome | 28.02% | 0.89% | OR | 65.9  (48.1-99.1) | Age and sex |
| Atrial septal defect, isolated | Norway | Brodwall et al. (2018)[56] | Down syndrome | 10.79% | 0.13% | PR | 85  (72-101) | Maternal age and birth year |
| Atrial septal defects and patent foramen ovale | US | Torfs et al. (1998)[54] | Down syndrome | 6.29% | 0.09% | RR | 70.7* | None |
| Atrioventricular septal defect | Norway | Brodwall et al. (2018)[56] | Down syndrome | 20.30% | 0.02% | PR | 850  (717-1008) | Maternal age and birth year |
| Atrioventricular septal defect | US | Torfs et al. (1998)[54] | Down syndrome | 17.14% | 0.02% | RR | 1009.1* | None |
| Atrioventricular septal defect | South Korea | Cho et al. (2020)[57] | Down syndrome | 9.82% | 0.02% | OR | 510  (126.7-999) | Age and sex |
| Heart septal defects | Norway | Brodwall et al. (2018)[56] | Down syndrome | 25.74% | 0.61% | PR | 43  (39-47) | Maternal age and birth year |
| Tetralogy of Fallot | Norway | Brodwall et al. (2018)[56] | Down syndrome | 1.12% | 0.02% | PR | 48  (28-81) | Maternal age and birth year |
| Tetralogy of Fallot | US | Torfs et al. (1998)[54] | Down syndrome | 2.52% | 0.03% | RR | 77.4* | None |
| Tetralogy of Fallot | South Korea | Cho et al. (2020)[57] | Down syndrome | 2.84% | 0.07% | OR | 42.1  (19.3-92.3) | Age and sex |
| Ventricular septal defect | US | Torfs et al. (1998)[54] | Down syndrome | 6.37% | 0.07% | RR | 94.5* | None |
| Ventricular septal defect | South Korea | Cho et al. (2020)[57] | Down syndrome | 19.88% | 0.35% | OR | 88.1  (57.9-134.1) | Age and sex |
| Ebstein's anomaly | Norway | Brodwall et al. (2018)[56] | Down syndrome | 0.08% | 0.01% | PR | 14  (1.9-99) | Maternal age and birth year |
| Hypoplastic left ventricle | US | Torfs et al. (1998)[54] | Down syndrome | 0.66% | 0.01% | RR | 47.1* | None |
| Hypoplastic right heart | US | Torfs et al. (1998)[54] | Down syndrome | 0.80% | 0.02% | RR | 49* | None |
| Pulmonary valve defects | US | Torfs et al. (1998)[54] | Down syndrome | 0.49% | 0.04% | RR | 13.6* | None |
| Pulmonary valve atresia/stenosis | South Korea | Cho et al. (2020)[57] | Down syndrome | 0.58% | 0.06% | OR | 10 (3.8-26.6) | Age and sex |
| Triscuspid valve defects | US | Torfs et al. (1998)[54] | Down syndrome | 11.44% | 0.14% | RR | 83.7* | None |
| Aortic valve defects | US | Torfs et al. (1998)[54] | Down syndrome | 2.59% | 0.05% | RR | 55.6* | None |
| Hypoplastic left heart syndrome | US | Torfs et al. (1998)[54] | Down syndrome | 0.04% | 0.02% | RR | 1.8 | None |
| Mitral valve defects | US | Torfs et al. (1998)[54] | Down syndrome | 4.87% | 0.06% | RR | 81.7* | None |
| Aortic valve stenosis | Norway | Brodwall et al. (2018)[56] | Down syndrome | 0.08% | 0.03% | PR | 2.7  (0.4-19) | Maternal age and birth year |
| Anamalies of the coronary artery or sinus | US | Torfs et al. (1998)[54] | Down syndrome | 0.62% | 0.01% | RR | 57.6* | None |
| Dextrocardia | US | Torfs et al. (1998)[54] | Down syndrome | 0.07% | 0.02% | RR | 4 | None |
| Anomalies of the pulmonary artery | US | Torfs et al. (1998)[54] | Down syndrome | 2.42% | 0.04% | RR | 57.5* | None |
| Aortic hypoplasia | Norway | Brodwall et al. (2018)[56] | Down syndrome | 0.56% | 0.02% | PR | 32  (15-67) | Maternal age and birth year |
| Coarctation of aorta | Norway | Brodwall et al. (2018)[56] | Down syndrome | 0.80% | 0.03% | PR | 30  (16-56) | Maternal age and birth year |
| Coarctation of aorta | US | Torfs et al. (1998)[54] | Down syndrome | 0.97% | 0.04% | RR | 25.9* | None |
| Coarctation of aorta | South Korea | Cho et al. (2020)[57] | Down syndrome | 0.77% | 0.01% | OR | 80  (10.6-603.2) | Age and sex |
| Hypoplasia of the aorta | US | Torfs et al. (1998)[54] | Down syndrome | 2.21% | 0.03% | RR | 77* | None |
| Interrupted aortic arch | US | Torfs et al. (1998)[54] | Down syndrome | 0.07% | 0.01% | RR | 13.4* | None |
| Overriding aorta | US | Torfs et al. (1998)[54] | Down syndrome | 0.35% | 0.00% | RR | 200.1* | None |
| Patent ductal arteriosus | US | Torfs et al. (1998)[54] | Down syndrome | 24.40% | 0.16% | RR | 151.6* | None |
| Patent ductal arteriosus | South Korea | Cho et al. (2020)[57] | Down syndrome | 19.07% | 0.48% | OR | 56.9  (40.1-80.8) | Age and sex |
| Persistent ductus arteriosus (isolated) | Norway | Brodwall et al. (2018)[56] | Down syndrome | 4.80% | 0.24% | PR | 20  (15-25) | Maternal age and birth year |
| Persistent right aortic arch | US | Torfs et al. (1998)[54] | Down syndrome | 0.48% | 0.02% | RR | 33.2* | None |
| Anomalies of the great veins | US | Torfs et al. (1998)[54] | Down syndrome | 1.35% | 0.03% | RR | 40.8* | None |
| Single umbilical artery | US | Torfs et al. (1998)[54] | Down syndrome | 0.76% | 0.04% | RR | 18.6* | None |
| Choanal atresia | US | Torfs et al. (1998)[54] | Down syndrome | 0.07% | 0.02% | RR | 4.6 | None |
| Anomalies of the larynx, trachea, or bronchus | US | Torfs et al. (1998)[54] | Down syndrome | 1.42% | 0.05% | RR | 29.9* | None |
| Anomalies of the lung | US | Torfs et al. (1998)[54] | Down syndrome | 1.07% | 0.07% | RR | 15.8* | None |
| Cleft palate without cleft lip | US | Torfs et al. (1998)[54] | Down syndrome | 0.31% | 0.07% | RR | 6.3* | None |
| Cleft lip with or without cleft palate and without holoprosencephaly | US | Torfs et al. (1998)[54] | Down syndrome | 0.04% | 0.10% | RR | 0.3 | None |
| Congenital defects of digestive system | US | Torfs et al. (1998)[54] | Down syndrome | 10.89% | 0.56% | RR | 19.6* | None |
| Tracheo-esophageal fistula, esophageal, atresia/stenosis | US | Torfs et al. (1998)[54] | Down syndrome | 0.69% | 0.03% | RR | 26.4* | None |
| Pyloric stenosis | US | Torfs et al. (1998)[54] | Down syndrome | 0.17% | 0.17% | RR | 1 | None |
| Atresia and stenosis of small intestine | US | Torfs et al. (1998)[54] | Down syndrome | 4.80% | 0.03% | RR | 142.2* | None |
| Anal atresia/stenosis or missing or imperforate anus | US | Torfs et al. (1998)[54] | Down syndrome | 1.38% | 0.04% | RR | 33.5* | None |
| Ectopic anus | US | Torfs et al. (1998)[54] | Down syndrome | 1.11% | 0.02% | RR | 66.7* | None |
| Hirschsprung disease | US | Torfs et al. (1998)[54] | Down syndrome | 1.38% | 0.01% | RR | 101.8* | None |
| Malrotation of the intestines | US | Torfs et al. (1998)[54] | Down syndrome | 1.52% | 0.03% | RR | 44.7* | None |
| Bile duct atresia | US | Torfs et al. (1998)[54] | Down syndrome | 0.07% | 0.01% | RR | 7.9* | None |
| Annular pancreas | US | Torfs et al. (1998)[54] | Down syndrome | 1.42% | 0.00% | RR | 430.3* | None |
| Undescended testicles | US | Torfs et al. (1998)[54] | Down syndrome | 7.22% | 0.19% | RR | 37.7* | None |
| Renal and urinary tract anomalies | US | Kupferman et al. (2009)[66] | Down syndrome | 3.20% | 0.70% | OR | 4.5  (3.8-5.4) | None |
| Obstructive defects of renal pelvis | US | Torfs et al. (1998)[54] | Down syndrome | 1.83% | 0.13% | RR | 14.2* | None |
| Horseshoe kidney | US | Torfs et al. (1998)[54] | Down syndrome | 0.07% | 0.01% | RR | 11.7* | None |
| Absence of bladder or urethra | US | Torfs et al. (1998)[54] | Down syndrome | 0.00% | 0.00% | RR | N/A | None |
| Exstrophy of urinary bladder | US | Torfs et al. (1998)[54] | Down syndrome | 0.00% | 0.00% | RR | N/A | None |
| Upper or lower limb reduction defects | US | Torfs et al. (1998)[54] | Down syndrome | 1.31% | 0.05% | RR | 27.6* | None |
| Clubfoot | US | Torfs et al. (1998)[54] | Down syndrome | 0.93% | 0.12% | RR | 7.9* | None |
| Polydactyly | US | Torfs et al. (1998)[54] | Down syndrome | 0.93% | 0.10% | RR | 9.2* | None |
| Syndactyly | US | Torfs et al. (1998)[54] | Down syndrome | 2.45% | 0.09% | RR | 26.1* | None |
| Gastroschisis | US | Torfs et al. (1998)[54] | Down syndrome | 0.00% | 0.02% | RR | N/A | None |
| Morgagni hernia | US | Torfs et al. (1998)[54] | Down syndrome | 0.14% | 0.00% | RR | 245.9* | None |
| Omphalocele | US | Torfs et al. (1998)[54] | Down syndrome | 0.03% | 0.02% | RR | 2 | None |
| Other diaphragmatic hernias | US | Torfs et al. (1998)[54] | Down syndrome | 0.07% | 0.03% | RR | 2.6 | None |

Abbreviation: OR: odds ratio; PR: prevalence ratio; RR: relative risk; SMR: standardised morbidity ratio.

*P<0.05

## **Table S12. Comparisons of incidence rate (/10,000 person years) or incidence proportion between people with ID and people without ID or the general population**

| Disorders | Location | Study | Genetic syndrome | Incidence in ID | Incidence in comparison group | Measurement type | Effect size | Covariates |
| --- | --- | --- | --- | --- | --- | --- | --- | --- |
| Neoplasms(C00-D48) | |  |  |  |  |  |  |  |
| Buccal cavity | Denmark | Hasle et al. (2016)[69] | Down syndrome | 0 | N/A | SIR | 0.0  (0.00-1.15) | Age, sex and period |
| Otorhinolaryngeal | Finland | Patja et al. (2001)[67] | Not specified | 1.34 | N/A | SIR | 1.5  (0.6-3.0) | Age, sex and calendar year |
| All solid tumours | Denmark | Hasle et al. (2016)[69] | Down syndrome | 6.14 | N/A | SIR | 0.45  (0.34-0.59) | Age, sex and period |
| All cancers | Denmark | Hjortshøj et al. (2007)[74] | Bardet-Biedl syndrome | 8.31 | N/A | SIR | 0.46  (0.1-1.7) | Age, sex and calendar year |
| All cancers | Denmark | Hasle et al. (2016)[69] | Down syndrome | 12.62 | N/A | SIR | 0.84  (0.70-1.02) | Age, sex and period |
| All cancers | Denmark | Schultz-Pedersen et al. (2001)[75] | Fragile X syndrome | 1.35% | N/A | SIR | 0.28  (0.1-0.8) | Age, sex and calendar year |
| All cancers | Finland | Patja et al. (2001)[67] | Not specified | 33.12 | N/A | SIR | 0.9  (0.8-1.0) | Age, sex and calendar year |
| All cancers | Israel | Boker et al. (2001)[71] | Down syndrome | 6.38 | N/A | SIR | 4.67  (1.9-9.6) | age, sex, place of birth (for some), nationality and calendar year |
| Digestive system | Denmark | Hasle et al. (2016)[69] | Down syndrome | 0.89 | N/A | SIR | 0.52  (0.22-1.03) | Age, sex and period |
| Gastrointestinal system | Finland | Patja et al. (2001)[67] | Not specified | 10.15 | N/A | SIR | 1.2  (0.9-1.5) | Age, sex and calendar year |
| Gallbladder | Finland | Patja et al. (2001)[67] | Not specified | 1.34 | N/A | SIR | 2.8  (1.1-5.8) | Age, sex and calendar year |
| Pancreas | Denmark | Schultz-Pedersen et al. (2001)[75] | Fragile X syndrome | 0.45% | N/A | SIR | 3.94  (0.1-22) | Age, sex and calendar year |
| Respiratory system | Denmark | Hasle et al. (2016)[69] | Down syndrome | 0.11 | N/A | SIR | 0.09  (0.00-0.49) | Age, sex and period |
| Respiratory system | Finland | Patja et al. (2001)[67] | Not specified | 3.83 | N/A | SIR | 0.7  (0.4-1.0) | Age, sex and calendar year |
| Bone | Finland | Patja et al. (2001)[67] | Not specified | 0 | N/A | SIR | 0.0  (0.0-6.3) | Age, sex and calendar year |
| Bone and cartilage | Denmark | Hasle et al. (2016)[69] | Down syndrome | 0 | N/A | SIR | 0.0  (0.00-5.12) | Age, sex and period |
| Skin (melanoma) | Denmark | Hasle et al. (2016)[69] | Down syndrome | 0.22 | N/A | SIR | 0.25  (0.03-0.89) | Age, sex and period |
| Skin (melanoma) | Finland | Patja et al. (2001)[67] | Not specified | 0.57 | N/A | SIR | 0.6  (0.1-1.9) | Age, sex and calendar year |
| Skin (non-melanoma) | Denmark | Hasle et al. (2016)[69] | Down syndrome | 0.67 | N/A | SIR | 0.24  (0.09-0.53) | Age, sex and period |
| Mesothelioma and soft tissue | Denmark | Hasle et al. (2016)[69] | Down syndrome | 0.33 | N/A | SIR | 0.24  (0.09-0.53) | Age, sex and period |
| Connective tissue | Finland | Patja et al. (2001)[67] | Not specified | 0.77 | N/A | SIR | 3.0  (0.8-7.7) | Age, sex and calendar year |
| Breast | Denmark | Hasle et al. (2016)[69] | Down syndrome | 0.33 | N/A | SIR | 0.16  (0.03-0.47) | Age, sex and period |
| Breast | Finland | Patja et al. (2001)[67] | Not specified | 4.4 | N/A | SIR | 0.9  (0.6-1.3) | Age, sex and calendar year |
| Female genital organs | Denmark | Hasle et al. (2016)[69] | Down syndrome | 0.56 | N/A | SIR | 0.47  (0.15-1.10) | Age, sex and period |
| Female genital organs | Finland | Patja et al. (2001)[67] | Not specified | 3.25 | N/A | SIR | 1.1  (0.6-1.7) | Age, sex and calendar year |
| Corpus uterus | Denmark | Schultz-Pedersen et al. (2001)[75] | Fragile X syndrome | 0.45% | N/A | SIR | 3.70  (0.1-21) | Age, sex and calendar year |
| Male genital organs | Finland | Patja et al. (2001)[67] | Not specified | 1.15 | N/A | SIR | 0.4  (0.1-0.8) | Age, sex and calendar year |
| Testicular | Denmark | Hasle et al. (2016)[69] | Down syndrome | 1.56 | N/A | SIR | 1.62  (0.89-2.72) | Age, sex and period |
| Testicular | Norway | Bjørge et al. (2008)[76] | Down syndrome | 0.24% | N/A | SIR | 5.5  (1.8-13) | Age, sex and period |
| Urinary tract | Denmark | Hasle et al. (2016)[69] | Down syndrome | 0.56 | N/A | SIR | 0.74  (0.24-1.72) | Age, sex and period |
| Urinary tract | Finland | Patja et al. (2001)[67] | Not specified | 0.77 | N/A | SIR | 0.3  (0.1-0.7) | Age, sex and calendar year |
| Urinary bladder | Denmark | Schultz-Pedersen et al. (2001)[75] | Fragile X syndrome | 0.45% | N/A | SIR | 1.97  (0.0-11) | Age, sex and calendar year |
| Eye | Finland | Patja et al. (2001)[67] | Not specified | 0 | N/A | SIR | 0.0  (0.0-10.7) | Age, sex and calendar year |
| Eye, brain and CNS | Denmark | Hasle et al. (2016)[69] | Down syndrome | 0.56 | N/A | SIR | 0.55  (0.18-1.29) | Age, sex and period |
| Nervous system | Finland | Patja et al. (2001)[67] | Not specified | 1.53 | N/A | SIR | 1.2  (0.5-2.4) | Age, sex and calendar year |
| Thyroid | Finland | Patja et al. (2001)[67] | Not specified | 1.34 | N/A | SIR | 2.1  (1.0-4.8) | Age, sex and calendar year |
| Endocrine organs | Denmark | Hasle et al. (2016)[69] | Down syndrome | 0 | N/A | SIR | 0.0  (0.00-2.40) | Age, sex and period |
| Hodgkin lymphoma | Denmark | Hasle et al. (2016)[69] | Down syndrome | 0.11 | N/A | SIR | 0.54  (0.01-3.00) | Age, sex and period |
| Leukaemia | Norway | Bjørge et al. (2008)[76] | Down syndrome | 1.99% | N/A | SIR | 36  (26-48) | Age, sex and period |
| Leukaemia | Sweden | Bjørge et al. (2008)[76] | Down syndrome | 2.06% | N/A | SIR | 36  (28-64) | Age, sex and period |
| Leukaemia | Israel | Boker et al. (2001)[71] | Down syndrome | 6.38 | N/A | SIR | 25.18  (10.40-53.40) | Age, sex, place of birth (for some), nationality and calendar year |
| Leukaemia | Finland | Patja et al. (2001)[67] | Not specified | 0.96 | N/A | SIR | 1.1  (0.4-2.5) | Age, sex and calendar year |
| Lymphomas and leukemia | Denmark | Hasle et al. (2016)[69] | Down syndrome | 6.48 | N/A | SIR | 5.50  (4.17-7.11) | Age, sex and period |
| Other leukemias | Denmark | Hasle et al. (2016)[69] | Down syndrome | 0.56 | N/A | SIR | 23.2  (7.48-54.2) | Age, sex and period |
| Non-Hodgkin lymphoma | Denmark | Hasle et al. (2016)[69] | Down syndrome | 0.22 | N/A | SIR | 0.55  (0.06-2.00) | Age, sex and period |
| Non-Hodgkin lymphoma | Finland | Patja et al. (2001)[67] | Not specified | 1.15 | N/A | SIR | 1.5  (0.5-3.2) | Age, sex and calendar year |
| Myeloma | Denmark | Hasle et al. (2016)[69] | Down syndrome | 0 | N/A | SIR | 0.0  (0.00-4.29) | Age, sex and period |
| Myeloma | Finland | Patja et al. (2001)[67] | Not specified | 0.19 | N/A | SIR | 0.4  (0.0-2.3) | Age, sex and calendar year |
| Acute non-lymphoblastic leukemia | Israel | Boker et al. (2001)[71] | Down syndrome | 0.91 | N/A | SIR | 4.90  (0.06-27.27) | Age, sex, place of birth (for some), nationality and calendar year |
| Lymphoid leukemia | Denmark | Hasle et al. (2016)[69] | Down syndrome | 3.35 | N/A | SIR | 13.0  (8.74-18.5) | Age, sex and period |
| Acute lymphoblastic leukemia | Israel | Boker et al. (2001)[71] | Down syndrome | 2.74 | N/A | SIR | 60  (12.06-175.3) | Age, sex, place of birth (for some), nationality and calendar year |
| Acute lymphoid leukemia | Sweden | Bjørge et al. (2008)[76] | Down syndrome | 0.78% | N/A | SIR | 18  (11-26) | Age, sex and period |
| Acute lymphoid leukemia | Norway | Bjørge et al. (2008)[76] | Down syndrome | 0.57% | N/A | SIR | 15  (7.5-25) | Age, sex and period |
| Acute myeloid leukemia | Sweden | Bjørge et al. (2008)[76] | Down syndrome | 1.09% | N/A | SIR | 141  (98-196) | Age, sex and period |
| Acute myeloid leukemia | Norway | Bjørge et al. (2008)[76] | Down syndrome | 1.09% | N/A | SIR | 115  (73-173) | Age, sex and period |
| Myeloid leukemia | Denmark | Hasle et al. (2016)[69] | Down syndrome | 2.12 | N/A | SIR | 11.8  (7.11-18.5) | Age, sex and period |
| Monocytoid leukemia | Denmark | Hasle et al. (2016)[69] | Down syndrome | 0.11 | N/A | SIR | 21.9  (0.20-122) | Age, sex and period |
| Hodgkin disease | Finland | Patja et al. (2001)[67] | Not specified | 0 | N/A | SIR | 0.0  (0.0-2.4) | Age, sex and calendar year |
| Injury, poisoning and certain other consequences of external causes (S00-T98) | | | |  |  |  |  |  |
| Injuries (self-injury excluded) | UK | Finlayson et al. (2010)[35] | Not specified | 20.40% | 11.50% | SIR | 1.78 | Age |

Abbreviation: SIR: standardised incidence ratio; N/A: not applicable

References:

1. Cooper S-A, McLean G, Guthrie B, McConnachie A, Mercer S, Sullivan F, et al. Multiple physical and mental health comorbidity in adults with intellectual disabilities: population-based cross-sectional analysis. *BMC Fam*. 2015;16(1):110. doi: 10.1186/s12875-015-0329-3.

2. Lunsky Y, Durbin A, Brown HK, Bansal S, Heifetz M, Antoniou T. Health profiles and associated service use among adults with HIV and intellectual and developmental disabilities. *Aids*. 2017;31(5):697-705. doi: <https://dx.doi.org/10.1097/QAD.0000000000001361>.

3. Cooper SA, Hughes-McCormack L, Greenlaw N, McConnachie A, Allan L, Baltzer M, et al. Management and prevalence of long-term conditions in primary health care for adults with intellectual disabilities compared with the general population: A population-based cohort study. *J Appl Res Intellect Disabil*. 2018;31 Suppl 1:68-81. doi: <https://dx.doi.org/10.1111/jar.12386>.

4. Carey IM, Shah SM, Hosking FJ, DeWilde S, Harris T, Beighton C, et al. Health characteristics and consultation patterns of people with intellectual disability: a cross-sectional database study in English general practice. *Br J Gen Pract*. 2016;66(645):e264-70. doi: <https://dx.doi.org/10.3399/bjgp16X684301>.

5. Fabia J, Drolette M. Malformations and leukemia in children with Down's syndrome. *Pediatrics*. 1970;45(1):60.

6. Leonard S, Bower C, Petterson B, Leonard H. Medical aspects of school‐aged children with Down syndrome. *Dev Med Child Neurol*. 1999;41(10):683-8. doi: 10.1111/j.1469-8749.1999.tb00523.x.

7. McGrother CW, Marshall B. Recent trends in incidence, morbidity and survival in Down's syndrome. *J Ment Defic Res*. 1990;34(Pt 1):49-57.

8. Arnell H, Fischler B. Population-based study of incidence and clinical outcome of neonatal cholestasis in patients with Down syndrome. *J Pediatr*. 2012;161(5):899-902. doi: <https://dx.doi.org/10.1016/j.jpeds.2012.04.037>.

9. Beange H, McElduff A, Baker W. Medical disorders of adults with mental retardation: a population study. *Am J Ment Retard*. 1995;99(6):595-604.

10. Kapell D, Nightingale B, Rodriguez A, Lee JH, Zigman WB, Schupf N. Prevalence of chronic medical conditions in adults with mental retardation: comparison with the general population. *Ment Retard*. 1998;36(4):269-79.

11. Määttä T, Määttä J, Tervo-Määttä T, Taanila A, Kaski M, Iivanainen M. Healthcare and guidelines: A population-based survey of recorded medical problems and health surveillance for people with Down syndrome. *J Intellect Dev Disabil*. 2011;36(2):118-26. doi: 10.1080/13668250.2011.570253.

12. Jaruratanasirikul S, Limpitikul W, Dissaneevate P, Booncharoen P, Tantichantakarun P. Comorbidities in Down syndrome livebirths and health care intervention: an initial experience from the birth defects registry in Southern Thailand. *World J Pediatr*. 2017;13(2):152-7. doi: <https://dx.doi.org/10.1007/s12519-016-0093-z>.

13. Prasher VP, Glenn S, Cunningham C, Arshad H, Glenholmes P, Kirby A. Health morbidity and access to services by young adults with Down syndrome. *Int J Dev Disabil*. 2014;60(1):26-34. doi: <http://dx.doi.org/10.1179/204738713X.13673354444083>.

14. Henderson A, Lynch SA, Wilkinson S, Hunter M. Adults with Down's sydrome: The prevalence of complications and health care in the community. *Br J Gen Pract*. 2007;57(534):50-5.

15. Diene G, Mimoun E, Feigerlova E, Caula S, Molinas C, Grandjean H, et al. Endocrine disorders in children with Prader-Willi syndrome--data from 142 children of the French database. *Horm Res Paediatr*. 2010;74(2):121-8. doi: <https://dx.doi.org/10.1159/000313377>.

16. Cuypers M, Leijssen M, Bakker-van Gijssel EJ, Pouls KPM, Mastebroek MM, Naaldenberg J, et al. Patterns in the prevalence of diabetes and incidence of diabetic complications in people with and without an intellectual disability in Dutch primary care: Insights from a population-based data-linkage study. *Prim Care Diabetes*. 2021;15(2):372-7. doi: <http://dx.doi.org/10.1016/j.pcd.2020.11.012>.

17. Mikulovic J, Marcellini A, Compte R, Duchateau G, Vanhelst J, Fardy PS, et al. Prevalence of overweight in adolescents with intellectual deficiency. Differences in socio-educative context, physical activity and dietary habits. *Appetite*. 2011;56(2):403-7. doi: <https://dx.doi.org/10.1016/j.appet.2010.12.006>.

18. Simila S, Niskanen P. Underweight and overweight cases among the mentally retarded. *J Ment Defic Res*. 1991;35(Pt 2):160-4.

19. Boyle A, Melville CA, Morrison J, Allan L, Smiley E, Espie CA, et al. A cohort study of the prevalence of sleep problems in adults with intellectual disabilities. *J Sleep Res*. 2010;19(1 Pt 1):42-53. doi: <https://dx.doi.org/10.1111/j.1365-2869.2009.00788.x>.

20. Gale L, Naqvi H, Russ L. Asthma, smoking and BMI in adults with intellectual disabilities: a community-based survey. *J Intellect Disabil Res*. 2009;53(9):787-96. doi: <https://dx.doi.org/10.1111/j.1365-2788.2009.01192.x>.

21. Melville C, Cooper S, Morrison J, Allan L, Smiley E, Williamson A. The prevalence and determinants of obesity in adults with intellectual disabilities. *J Appl Res Intellect Disabil*. 2008;21(5):425-37. doi: <http://dx.doi.org/10.1111/j.1468-3148.2007.00412.x>.

22. Hove O. Weight survey on adult persons with mental retardation living in the community. *Res Dev Disabil*. 2004;25(1):9-17.

23. Garg A, Strunk A, Midura M, Papagermanos V, Pomerantz H. Prevalence of hidradenitis suppurativa among patients with Down syndrome: a population-based cross-sectional analysis. *Br J Dermatol*. 2018;178(3):697-703. doi: <http://dx.doi.org/10.1111/bjd.15770>.

24. Melville CA, Cooper SA, McGrother CW, Thorp CF, Collacott R. Obesity in adults with Down syndrome: a case-control study. *J Intellect Disabil Res*. 2005;49(Pt 2):125-33.

25. Hamilton J, Yaneza MM, Clement WA, Kubba H. The prevalence of airway problems in children with Down's syndrome. *Int J Pediatr Otorhinolaryngol*. 2016;81:1-4. doi: <https://dx.doi.org/10.1016/j.ijporl.2015.11.027>.

26. Forsgren L, Edvinsson SO, Blomquist HK, Heijbel J, Sidenvall R. Epilepsy in a population of mentally retarded children and adults. *Epilepsy Res*. 1990;6(3):234-48.

27. Arvio M, Sillanpaa M. Prevalence, aetiology and comorbidity of severe and profound intellectual disability in Finland. *J Intellect Disabil Res*. 2003;47(Pt 2):108-12.

28. Benassi G, Guarino M, Cammarata S, Cristoni P, Fantini MP, Ancona A, et al. An epidemiological study on severe mental retardation among schoolchildren in Bologna, Italy. *Dev Med Child Neurol*. 1990;32(10):895-901.

29. Gustavson KH, Hagberg B, Hagberg G, Sars K. Severe mental retardation in a Swedish county I. Epidemiology, gestational age, birth weight and associated CNS handicaps in children born 1959–70. *Acta Pædiatrica*. 1977;66(3):373-9. doi: 10.1111/j.1651-2227.1977.tb07910.x.

30. McQueen PC, Spence MW, Garner JB, Pereira LH, Winsor EJ. Prevalence of major mental retardation and associated disabilities in the Canadian Maritime Provinces. *Am J Ment Defic*. 1987;91(5):460-6.

31. Wellesley DG, Hockey KA, Montgomery PD, Stanley FJ. Prevalence of intellectual handicap in Western Australia: a community study. *Med J Aust*. 1992;156(2):94-6, 100, 2.

32. Christianson AL, Zwane ME, Manga P, Rosen E, Venter A, Downs D, et al. Children with intellectual disability in rural South Africa: prevalence and associated disability. *J Intellect Disabil Res*. 2002;46(Pt 2):179-86.

33. Shepherd C, Hosking G. Epilepsy in school children with intellectual impairments in Sheffield: the size and nature of the problem and the implications for service provision. *J Ment Defic Res*. 1989;33(Pt 6):511-4.

34. Matthews T, Weston N, Baxter H, Felce D, Kerr M. A general practice-based prevalence study of epilepsy among adults with intellectual disabilities and of its association with psychiatric disorder, behaviour disturbance and carer stress. *J Intellect Disabil Res*. 2008;52(Pt 2):163-73. doi: <https://dx.doi.org/10.1111/j.1365-2788.2007.01025.x>.

35. Finlayson J, Morrison J, Jackson A, Mantry D, Cooper SA. Injuries, falls and accidents among adults with intellectual disabilities. Prospective cohort study. *J Intellect Disabil Res*. 2010;54(11):966-80. doi: <https://dx.doi.org/10.1111/j.1365-2788.2010.01319.x>.

36. Morgan CL, Baxter H, Kerr MP. Prevalence of epilepsy and associated health service utilization and mortality among patients with intellectual disability. *Am J Ment Retard*. 2003;108(5):293-300.

37. Janicki MP, Maceachron AE. Residential, health, and social service needs of elderly developmentally disabled persons. *The Gerontologist*. 1984;24(2):128. doi: 10.1093/geront/24.2.128.

38. McGrother CW, Bhaumik S, Thorp CF, Hauck A, Branford D, Watson JM. Epilepsy in adults with intellectual disabilities: prevalence, associations and service implications. *Seizure*. 2006;15(6):376-86.

39. Bishop L, McLean KJ, Rubenstein E. Epilepsy in adulthood: Prevalence, incidence, and associated antiepileptic drug use in autistic adults in a state Medicaid system. *Autism*. 2020. doi: <http://dx.doi.org/10.1177/1362361320942982>.

40. Johannsen P, Christensen JE, Goldstein H, Nielsen VK, Mai J. Epilepsy in Down syndrome--prevalence in three age groups. *Seizure*. 1996;5(2):121-5.

41. Kristianslund O, Drolsum L. Prevalence of Keratoconus in Persons with down Syndrome in a National Registry in Norway. *JAMA Network Open*. 2021;4(3):e210814. doi: <http://dx.doi.org/10.1001/jamanetworkopen.2021.0814>.

42. Atladottir HO, Schendel DE, Parner ET, Henriksen TB. A Descriptive Study on the Neonatal Morbidity Profile of Autism Spectrum Disorders, Including a Comparison with Other Neurodevelopmental Disorders. *J Autism Dev Disord*. 2015;45(8):2429-42. doi: <https://dx.doi.org/10.1007/s10803-015-2408-7>.

43. Austeng ME, Akre H, Overland B, Abdelnoor M, Falkenberg ES, Kvaerner KJ. Otitis media with effusion in children with in Down syndrome. *Int J Pediatr Otorhinolaryngol*. 2013;77(8):1329-32. doi: <https://dx.doi.org/10.1016/j.ijporl.2013.05.027>.

44. Barr E, Dungworth J, Hunter K, McFarlane M, Kubba H. The prevalence of ear, nose and throat disorders in preschool children with Down's syndrome in Glasgow. *Scott Med J*. 2011;56(2):98-103. doi: <https://dx.doi.org/10.1258/smj.2011.011036>.

45. Yaneza MM, Hunter K, Irwin S, Kubba H. Hearing in school-aged children with trisomy 21 - results of a longitudinal cohort study in children identified at birth. *Clin Otolaryngol*. 2016;41(6):711-7. doi: <https://dx.doi.org/10.1111/coa.12606>.

46. Meuwese-Jongejeugd A, Vink M, van Zanten B, Verschuure H, Eichhorn E, Koopman D, et al. Prevalence of hearing loss in 1598 adults with an intellectual disability: cross-sectional population based study. *Int J Audiol*. 2006;45(11):660-9.

47. Austeng ME, Akre H, Falkenberg E-S, Overland B, Abdelnoor M, Kvaerner KJ. Hearing level in children with Down syndrome at the age of eight. *Res Dev Disabil*. 2013;34(7):2251-6. doi: <http://dx.doi.org/10.1016/j.ridd.2013.04.006>.

48. Park AH, Wilson MA, Stevens PT, Harward R, Hohler N. Identification of hearing loss in pediatric patients with Down syndrome. *Otolaryngol Head Neck Surg*. 2012;146(1):135-40. doi: <https://dx.doi.org/10.1177/0194599811425156>.

49. Tedeschi AS, Roizen NJ, Taylor HG, Murray G, Curtis CA, Parikh AS. The prevalence of congenital hearing loss in neonates with Down syndrome. *J Pediatr*. 2015;166(1):168-71. doi: <https://dx.doi.org/10.1016/j.jpeds.2014.09.005>.

50. Jansson U, Johansson C. Down syndrome and celiac disease. *J Pediatr Gastroenterol Nutr*. 1995;21(4):443-5.

51. Thomson AK, Glasson EJ, Bittles AH. A long-term population-based clinical and morbidity profile of Angelman syndrome in Western Australia: 1953-2003. *Disabil Rehabil*. 2006;28(5):299-305. doi: <http://dx.doi.org/10.1080/09638280500190631>.

52. Burke É, Carroll R, O’Dwyer M, Walsh JB, McCallion P, McCarron M. Quantitative examination of the bone health status of older adults with intellectual and developmental disability in Ireland: a cross-sectional nationwide study. *BMJ Open*. 2019;9(4):e026939. doi: 10.1136/bmjopen-2018-026939.

53. Weijerman ME, van Furth AM, van der Mooren MD, van Weissenbruch MM, Rammeloo L, Broers CJ, et al. Prevalence of congenital heart defects and persistent pulmonary hypertension of the neonate with Down syndrome. *Eur J Pediatr*. 2010;169(10):1195-9. doi: <https://dx.doi.org/10.1007/s00431-010-1200-0>.

54. Torfs CP, Christianson RE. Anomalies in Down syndrome individuals in a large population-based registry. *Am J Med Genet*. 1998;77(5):431-8. doi: <http://dx.doi.org/10.1002/%28SICI%291096-8628%2819980605%2977:5%3C431::AID-AJMG15%3E3.0.CO;2-J>.

55. Santoro M, Coi A, Spadoni I, Bianchi F, Pierini A. Sex differences for major congenital heart defects in Down Syndrome: A population based study. *Eur J Med Genet*. 2018;61(9):546-50. doi: <http://dx.doi.org/10.1016/j.ejmg.2018.05.013>.

56. Brodwall K, Greve G, Leirgul E, Klungsoyr K, Holmstrom H, Vollset SE, et al. The five-year survival of children with Down syndrome in Norway 1994-2009 differed by associated congenital heart defects and extracardiac malformations. *Acta Paediatr*. 2018;107(5):845-53. doi: <http://dx.doi.org/10.1111/apa.14223>.

57. Cho WK, Lee NY, Han K, Suh BK, Park YG. The population prevalence, associations of congenital heart defect and mortality risk for down's syndrome in South Korea based on national health insurance service (NHIS) data. *Clin Epidemiol*. 2020;12:519-25. doi: <http://dx.doi.org/10.2147/CLEP.S251637>.

58. Kim MA, Lee YS, Yee NH, Choi JS, Choi JY, Seo K. Prevalence of congenital heart defects associated with Down syndrome in Korea. *J Korean Med Sci*. 2014;29(11):1544-9. doi: <https://dx.doi.org/10.3346/jkms.2014.29.11.1544>.

59. Irving CA, Chaudhari MP. Cardiovascular abnormalities in Down's syndrome: spectrum, management and survival over 22 years. *Arch Dis Child*. 2012;97(4):326-30. doi: <https://dx.doi.org/10.1136/adc.2010.210534>.

60. Scott C, Thame M. The incidence of cardiac lesions among children with Down's syndrome in Jamaica - A prospective study. *West Indian Med J*. 2014;63(7):693-7. doi: <http://dx.doi.org/10.7727/wimj.2013.216>.

61. So SA, Urbano RC, Hodapp RM. Hospitalizations of infants and young children with Down syndrome: evidence from inpatient person-records from a statewide administrative database. *J Intellect Disabil Res*. 2007;51(Pt 12):1030-8.

62. Bergström S, Carr H, Petersson G, Stephansson O, Bonamy A-KE, Dahlström A, et al. Trends in Congenital Heart Defects in Infants With Down Syndrome. *Pediatrics*. 2016;138(1). doi: 10.1542/peds.2016-0123.

63. Freeman SB, Taft LF, Dooley KJ, Allran K, Sherman SL, Hassold TJ, et al. Population-based study of congenital heart defects in Down syndrome. *Am J Med Genet*. 1998;80(3):213. doi: 2-8.

64. Freeman SB, Bean LH, Allen EG, Tinker SW, Locke AE, Druschel C, et al. Ethnicity, sex, and the incidence of congenital heart defects: a report from the National Down Syndrome Project. *Genet Med*. 2008;10(3):173-80. doi: <https://dx.doi.org/10.1097/GIM.0b013e3181634867>.

65. Freeman SB, Torfs CP, Romitti PA, Royle MH, Druschel C, Hobbs CA, et al. Congenital gastrointestinal defects in Down syndrome: a report from the Atlanta and National Down Syndrome Projects. *Clin Genet*. 2009;75(2):180-4. doi: 10.1111/j.1399-0004.2008.01110.x.

66. Kupferman JC, Druschel CM, Kupchik GS. Increased prevalence of renal and urinary tract anomalies in children with Down syndrome. *Pediatrics*. 2009;124(4):e615-21. doi: <https://dx.doi.org/10.1542/peds.2009-0181>.

67. Patja K, Eero P, Iivanainen M. Cancer incidence among people with intellectual disability. *J Intellect Disabil Res*. 2001;45(Pt 4):300-7.

68. Sullivan SG, Hussain R, Threlfall T, Bittles AH. The incidence of cancer in people with intellectual disabilities. *Cancer Causes Control*. 2004;15(10):1021-5.

69. Hasle H, Friedman JM, Olsen Jo H, Rasmussen SA. Low risk of solid tumors in persons with Down syndrome. *Genet Med*. 2016;18(11):1151-7. doi: <http://dx.doi.org/10.1038/gim.2016.23>.

70. Patja K, Pukkala E, Sund R, Iivanainen M, Kaski M. Cancer incidence of persons with Down syndrome in Finland: a population-based study. *Int J Cancer*. 2006;118(7):1769-72.

71. Boker LK, Blumstein T, Sadetzki S, Luxenburg O, Litvak I, Akstein E, et al. Incidence of leukemia and other cancers in Down syndrome subjects in Israel. *Int J Cancer*. 2001;93(5):741-4.

72. Sullivan SG, Hussain R, Glasson EJ, Bittles AH. The profile and incidence of cancer in Down syndrome. *J Intellect Disabil Res*. 2007;51(Pt 3):228-31.

73. Patja K, Sund R, Kaski M, Pukkala E. Cancer incidence among persons with Prader-Willi syndrome in Finland. *Int J Disabil Hum Dev*. 2008;7(1):69-72. doi: <http://dx.doi.org/10.1515/IJDHD.2008.7.1.69>.

74. Hjortshoj TD, Gronskov K, Rosenberg T, Brondum-Nielsen K, Olsen JH. Risk for cancer in patients with Bardet-Biedl syndrome and their relatives. *Am J Med Genet A*. 2007;143A(15):1699-702.

75. Schultz-Pedersen S, Hasle H, Olsen JH, Friedrich U. Evidence of decreased risk of cancer in individuals with fragile X. *Am J Med Genet*. 2001;103(3):226-30.

76. Bjorge T, Cnattingius S, Lie RT, Tretli S, Engeland A. Cancer risk in children with birth defects and in their families: A population based cohort study of 5.2 million children from Norway and Sweden. *Cancer Epidemiol Biomarkers Prev*. 2008;17(3):500-6. doi: <http://dx.doi.org/10.1158/1055-9965.EPI-07-2630>.

77. Ostermaier KK, Weaver AL, Myers SM, Stoeckel RE, Katusic SK, Voigt RG. Incidence of Celiac Disease in Down Syndrome: A Longitudinal, Population-Based Birth Cohort Study. *Clin Pediatr*. 2020;59(12):1086-91. doi: <https://dx.doi.org/10.1177/0009922820941247>.
