# Supplementary material for: Prevalence and incidence of physical health conditions in people with intellectual disability – a systematic review
Source: PLoS One. 2021 Aug 24;16(8):e0256294. doi: 10.1371/journal.pone.0256294 (PMC8384165; doi:10.1371/journal.pone.0256294)
Supplement: S5 File — (DOCX) [file pone.0256294.s005.docx]

**Search terms and steps**

**MEDLINE**

**Search terms:**

1. **Intellectual disability**
2. Exp intellectual disability (including exp all subheading) and intellectual disabilit* as keyword, or
3. Mental retardation as keyword, or
4. learning disabilit* as keyword, or
5. mental handicap as keyword, or
6. intellectual developmental disorder* as keyword
7. **Comorbid physical health conditions**
8. Health condition* as keyword, or
9. Health problem* as keyword, or
10. Physical disorder* as keyword, or
11. Physical disease* as keyword, or
12. Physical condition* as keyword, or
13. Comorbidity (Mesh term) and keyword (comorbidit*), or
14. Comorbid, or
15. exp “bacterial infections and mycoses”/ or exp virus diseases/ or exp parasitic diseases/ or exp neoplasms/ or exp musculoskeletal disease/ or exp digestive system diseases/ or exp stomatognathic disease/ or exp respiratory tract diseases/ or exp otorhinolaryngologic diseases/ or exp nervous system diseases/ or exp eye diseases/ or exp male urogenital diseases/ or exp “female urogenital diseases and pregnancy complications”/ or exp cardiovascular diseases/ or exp “hemic and lymphatic diseases”/ or exp “congenital, hereditary, and neonatal diseases and abnormalities”/ or exp “skin and connective tissue diseases”/ or exp “nutritional and metabolic diseases”/ or exp endocrine system diseases/ or exp immune system diseases/ or exp “disorders of environmental origin”/ exp disease/
16. **Morbidity**
17. Prevalence (mesh term) and keyword in the title only, or
18. Incidence (mesh term) and keyword in the title only, or
19. Morbidity as keyword in the title only

Steps:

1 and 2 and 3

Limit:

- 1. English Language and Humans; and
  2. Publication type: journal article or observational or comparative study; and
  3. Not systematic review.

**PSYCINFO**

**Search terms:**

1. **Intellectual disability**
2. Exp intellectual developmental disorder (including exp crying cat syndrome/exp down’s syndrome/exp fetal alcohol syndrome/exp fragile x syndrome/exp prader willi syndrome/exp Williams syndrome) and intellectual developmental disorder* as keyword, or
3. Intellectual disabilit* as keyword, or
4. Mental retardation as keyword, or
5. learning disabilit* as keyword, or
6. mental handicap as keyword
7. **Comorbid health conditions**
8. Health condition* as keyword, or
9. Health problem* as keyword, or
10. Physical disorder* as keyword, or
11. Physical disease* as keyword, or
12. Exp Physical disorder as mesh term (including exp “blood and lymphatic disorders”/exp cachexia/exp cardiovascular disorders/exp chronically ill children/exp digestive system disorders/exp endocrine disorders/exp genetic disorders/exp health impairments/exp immunologic disorders/exp infectious disorders/exp metabolism disorders/exp musculoskeletal disorders/exp neonatal disorders/exp neoplasms/exp nervous system disorders/exp nutritional deficiencies/exp respiratory tract disorders/exp sense organ disorders/exp sensory system disorders/exp skin disorders/exp toxic disorders/exp urogenital disorders/exp vision disorders/), or
13. Physical condition* as keyword, or
14. Comorbidity (Mesh term) and as keyword in the title, or
15. Comorbid
16. **Morbidity**
17. Prevalence keyword, or
18. Incidence keyword, or
19. Morbidity keyword

Steps:

1 and 2 and 3

Limit:

1) English and human; and

2) Journal articles; and

3) Not review.

**EMBASE**

**Search terms:**

1. **Intellectual disability**
2. intellectual impairment and intellectual disabilit* as keyword, or
3. Mental retardation as keyword, or
4. Down syndrome/ WAGR syndrome/ X linked mental retardation/mental retardation malformation syndrome/, or
5. learning disabilit* as keyword, or
6. mental handicap as keyword, or
7. intellectual developmental disorder* as keyword
8. **Comorbid health conditions**
9. Health condition* as keyword, or
10. Health problem* as keyword, or
11. Physical disease exp or Physical disorder* as keyword, or
12. Physical condition* as keyword, or
13. Comorbidity exp and comorbidit* keyword, or
14. Comorbid, or
15. Physical disease* se keyword
16. **Morbidity**
17. Prevalence (mesh term) and keyword, or
18. Incidence (mesh term) and keyword, or
19. Prevalence or incidence as keyword in the title only, or
20. Morbidity in the title only

Steps:

1 and 2 and 3

Limit:

1) Human; and

2) English language; and

3) Non review - article or article in press.
